# Supplementary material for: Detection of intracranial hypertension in children using optical coherence tomography: a systematic review
Source: BMJ Open. 2021 Aug 11;11(8):e046935. doi: 10.1136/bmjopen-2020-046935 (PMC8359522; doi:10.1136/bmjopen-2020-046935)
Supplement: Supplementary data [file bmjopen-2020-046935supp001.pdf]

Supplemental Data

Contents

Appendix 1: Search terms 2
Appendix 2: Screening questions 3
Appendix 3: Data extraction tool, adapted from the Cochrane Collaboration 4
Appendix 4: NIH Quality Assessment Tool for Observational Cohort and Cross-Sectional Studies ... 7
Supplemental Table 1: Excluded studies with reasons 8

Appendix 1: Search terms

Intracranial pressure, intracranial hypertension, papilloedema, optical coherence tomography

1. Intracranial pressure
2. ICP
3. Intracerebral pressure
4. Intracranial hypertension
5. Cerebral swelling
6. Cerebral oedema
7. Cerebral edema
8. Brain swelling
9. Brain oedema
10. Brain edema
11. Papilloedema
12. Papilledema
13. Optical coherence tomography
14. OCT

Search combination:

1 OR 2 OR 3 OR 4 OR 5 OR 6 OR 7 OR 8 OR 9 OR 10 OR 11 OR 12

AND

13 or 14

Appendix 2: Screening questions

Instructions for screeners: Tick the appropriate box per screening question. If "yes" at Stage 1, proceed to Stage 2; if "yes" at Stage 2, include. If "no" at any stage, exclude. If "unclear", proceed to next stage. If still "unclear" after Stage 2, then submit to third arbitrator for verdict.

Stage 1: Title Screening

Does the study represent Level 4 evidence or above, i.e. case series, case-control studies, cohort studies, randomised controlled trials (RCTs) and systematic reviews?

Yes	
No	
Unclear	

Does the study pertain to the use of optical coherence tomography (OCT) to detect raised intracranial pressure (ICP) in children, defined as 0 to 18 years of age?

Yes	
No	
Unclear	

Stage 2: Abstract screening

Does the study represent Level IV evidence or above, i.e. case series, cohort studies, case-control studies, randomised controlled trials (RCTs) and systematic reviews?

Yes	
No	
Unclear	

Does the study pertain to the use of optical coherence tomography (OCT) to detect raised intracranial pressure (ICP) in children, defined as 0 to 18 years of age?

Yes	
No	
Unclear	

Stage 3: Full paper screening

Does the study pertain to the use of optical coherence tomography (OCT) to detect raised intracranial pressure (ICP) in children, defined as 0 to 18 years of age?

Yes	
No	
Unclear	

Rufai SR, *et al. BMJ Open* 2021; 11:e046935. doi: 10.1136/bmjopen-2020-046935

Appendix 3: Data extraction tool, adapted from the Cochrane Collaboration

General Information

Study title:
Author(s):
Source:
Date of Extraction:

Methods

Aim of study	
Study design	
Inclusion criteria	
Exclusion criteria	
Method of recruitment	
Method of randomisation (if applicable)	
Blinding (if applicable)	
Study duration	
Ethics approval	

Participants

Population description	
Setting	
Total number randomised	
Mean age	
Male/Female %	

Intervention 1

Group name	
Number in group	
Description	
Intervention parameter 1	
Intervention parameter 2 (if applicable)	
Intervention parameter 3 (if applicable)	
Intervention parameter 4 (if applicable)	
Intervention parameter 5 (if applicable)	
Intervention parameter 6 (if applicable)	
Intervention parameter 7 (if applicable)	
Intervention parameter 8 (if applicable)	
Drop-outs from group	

Outcome 1

Outcome group name	
Time points measured	

Outcome definition	
Outcome parameter 1	
Outcome parameter 2 (if applicable)	
Outcome parameter 3 (if applicable)	
Outcome parameter 4 (if applicable)	
Outcome parameter 5 (if applicable)	
Outcome parameter 6 (if applicable)	
Outcome parameter 7 (if applicable)	
Outcome parameter 8 (if applicable)	
Person measuring	
Imputation of missing data	

Intervention 2 (if applicable)

Intervention group name	
Number in group	
Description	
Intervention parameter 1	
Intervention parameter 2 (if applicable)	
Intervention parameter 3 (if applicable)	
Intervention parameter 4 (if applicable)	
Intervention parameter 5 (if applicable)	
Intervention parameter 6 (if applicable)	
Intervention parameter 7 (if applicable)	
Intervention parameter 8 (if applicable)	
Drop-outs from group	

Outcome 2 (if applicable)

Outcome group name	
Time points measured	
Outcome definition	
Outcome parameter 1	
Outcome parameter 2 (if applicable)	
Outcome parameter 3 (if applicable)	
Outcome parameter 4 (if applicable)	
Outcome parameter 5 (if applicable)	
Outcome parameter 6 (if applicable)	
Outcome parameter 7 (if applicable)	
Outcome parameter 8 (if applicable)	
Person measuring	
Imputation of missing data	

Intervention 3 (if applicable)

Intervention group name	
Number in group	
Description	
Intervention parameter 1	
Intervention parameter 2 (if applicable)	
Intervention parameter 3 (if applicable)	
Intervention parameter 4 (if applicable)	
Intervention parameter 5 (if applicable)	
Intervention parameter 6 (if applicable)	
Intervention parameter 7 (if applicable)	
Intervention parameter 8 (if applicable)	
Drop-outs from group	

Outcome 3 (if applicable)

Outcome group name	
Time points measured	
Outcome definition	
Outcome parameter 1	
Outcome parameter 2 (if applicable)	
Outcome parameter 3 (if applicable)	
Outcome parameter 4 (if applicable)	
Outcome parameter 5 (if applicable)	
Outcome parameter 6 (if applicable)	
Outcome parameter 7 (if applicable)	
Outcome parameter 8 (if applicable)	
Person measuring	
Imputation of missing data	

Appendix 4: NIH Quality Assessment Tool for Observational Cohort and Cross-Sectional Studies

**Criteria**	**Yes**	**No**	**Other (CD, NR, NA)***
1. Was the research question or objective in this paper clearly stated?			
2. Was the study population clearly specified and defined?			
3. Was the participation rate of eligible persons at least 50%?			
4. Were all the subjects selected or recruited from the same or similar populations (including the same time period)? Were inclusion and exclusion criteria for being in the study prespecified and applied uniformly to all participants?			
5. Was a sample size justification, power description, or variance and effect estimates provided?			
6. For the analyses in this paper, were the exposure(s) of interest measured prior to the outcome(s) being measured?			
7. Was the timeframe sufficient so that one could reasonably expect to see an association between exposure and outcome if it existed?			
8. For exposures that can vary in amount or level, did the study examine different levels of the exposure as related to the outcome (e.g., categories of exposure, or exposure measured as continuous variable)?			
9. Were the exposure measures (independent variables) clearly defined, valid, reliable, and implemented consistently across all study participants?			
10. Was the exposure(s) assessed more than once over time?			
11. Were the outcome measures (dependent variables) clearly defined, valid, reliable, and implemented consistently across all study participants?			
12. Were the outcome assessors blinded to the exposure status of participants?			
13. Was loss to follow-up after baseline 20% or less?			
14. Were key potential confounding variables measured and adjusted statistically for their impact on the relationship between exposure(s) and outcome(s)?			

*CD, cannot determine; NA, not applicable; NR, not reported

**Quality Rating (Good, Fair, or Poor)**	
**Rater #1 initials:**	
**Rater #2 initials:**	
**Additional Comments (If POOR, please state why):**	

Rufai SR, *et al. BMJ Open* 2021; 11:e046935. doi: 10.1136/bmjopen-2020-046935

Supplemental Table 1: Excluded studies with reasons

Key: *Reasons for exclusion were as follows: A: adult studies (n=98); B: mixed adults and children without breakdown (n=27); C: conference abstracts (n=86); D: case reports (n=6); E: correspondence (n=4).

Number	Reference	Reason for exclusion*
1	Afonso, C.L., et al., Relationship between pattern electroretinogram, frequency-domain OCT, and automated perimetry in chronic papilledema from pseudotumor cerebri syndrome. Investigative Ophthalmology and Visual Science, 2015. 56(6): p. 3656-3665.	A
2	Afonso, J.M., et al., Spectral Domain-Optical Coherence Tomography As a New Diagnostic Marker for Idiopathic Normal Pressure Hydrocephalus. Frontiers in Neurology, 2017. 8: p. 10.	A
3	Aghsaei Fard, M., et al., Optic Nerve Head and Macular Optical Coherence Tomography Measurements in Papilledema Compared With Pseudopapilledema. Journal of neuro-ophthalmology : the official journal of the North American Neuro-Ophthalmology Society, 2019. 39(1): p. 28-34.	A
4	Albrecht, P., et al., Optical coherence tomography for the diagnosis and monitoring of idiopathic intracranial hypertension. Journal of neurology, 2017. 264(7): p. 1370-1380.	A
5	Alessi, G., et al., Evolution en tomographie par coherence optique de l'oedme papillaire dans l'hypertension intracranienne idiopathique traitee par mise en place de stent endosinusien du sinus lateralOptical coherence tomography in following up papilledema in idiopathic intracranial hypertension treated with lateral sinus stent placement. Journal Francais d'Ophtalmologie, 2010. 33(9): p. 637-648.	A
6	Anand, A., et al., Optical coherence tomography of the optic nerve head detects acute changes in intracranial pressure. Journal of Clinical Neuroscience, 2016. 29: p. 73-76.	A
7	Aojula, A., et al., Segmentation error in spectral domain optical coherence tomography measures of the retinal nerve fibre layer thickness in idiopathic intracranial hypertension. BMC ophthalmology, 2018. 17(1): p. 257.	A
8	Asian, F. and B. Ozkal, Optical coherence tomography measurements of the optic nerve head and retina in newly diagnosed idiopathic intracranial hypertension without loss of vision. Ceska a Slovenska Neurologie a Neurochirurgie, 2019. 82(3): p. 309-315.	A
9	Athappilly, G., et al., Ganglion Cell Complex Analysis as a Potential Indicator of Early Neuronal Loss in Idiopathic Intracranial Hypertension. Neuro-ophthalmology (Aeolus Press), 2019. 43(1): p. 10-17.	A
10	Auinger, P., et al., Baseline OCT Measurements in the Idiopathic Intracranial Hypertension Treatment Trial, Part II: Correlations and Relationship to Clinical Features. Investigative Ophthalmology & Visual Science, 2014. 55(12): p. 8173-8179.	A

Rufai SR, *et al. BMJ Open* 2021; 11:e046935. doi: 10.1136/bmjopen-2020-046935

11	Auinger, P., et al., Papilledema Outcomes from the Optical Coherence Tomography Substudy of the Idiopathic Intracranial Hypertension Treatment Trial. Ophthalmology, 2015. 122(9): p. 1939-1945.	A
12	Bassi, S.T. and K.P. Mohana, Optical coherence tomography in papilledema and pseudopapilledema with and without optic nerve head drusen. Indian journal of ophthalmology, 2014. 62(12): p. 1146-51.	A
13	Bilen, F.T. and H. Atilla, Peripapillary Vessel Density Measured by Optical Coherence Tomography Angiography in Idiopathic Intracranial Hypertension. Journal of Neuro-Ophthalmology, 2019. 39(3): p. 319-323.	A
14	Carey AR, Bosley TM, Miller NR, McCulley TJ, Henderson AD. Use of En Face Optical Coherence Tomography to Monitor Papilledema in Idiopathic Intracranial Hypertension: A Pilot Study [published online ahead of print, 2020 Mar 24]. J Neuroophthalmol. 2020;10.1097/WNO.0000000000000940. doi:10.1097/WNO.0000000000000940	A
15	Carta, A., et al., Optical coherence tomography is a useful tool in the differentiation between true edema and pseudoedema of the optic disc. PLoS ONE, 2018. 13(11).	A
16	Chen, J.J., et al., Optic disc edema in glial fibrillary acidic protein autoantibody-positive meningoencephalitis. Journal of Neuro-Ophthalmology, 2018. 38(3): p. 276-281.	A
17	Choi, S.S., et al., Changes in cellular structures revealed by ultra-high resolution retinal imaging in optic neuropathies. Investigative ophthalmology & visual science, 2008. 49(5): p. 2103-19.	A
18	Dreesbach, M., et al., Optic nerve head volumetry by optical coherence tomography in papilledema related to idiopathic intracranial hypertension. Translational Vision Science and Technology, 2020. 9(3).	A
19	Eren, Y., et al., Evaluation of optic nerve head changes with optic coherence tomography in patients with idiopathic intracranial hypertension. Acta Neurologica Belgica, 2019. 119(3): p. 351-357.	A
20	Fard, M.A., et al., Optic Nerve Head and Macular Optical Coherence Tomography Measurements in Papilledema Compared With Pseudopapilledema. Journal of Neuro-Ophthalmology, 2019. 39(1): p. 28-34.	A
21	Fard, M.A., et al., Optical Coherence Tomography Angiography in Optic Disc Swelling. American journal of ophthalmology, 2018. 191: p. 116-123.	A
22	Fard, M.A., et al., Optical Coherence Tomography Angiography in Papilledema Compared With Pseudopapilledema. Investigative ophthalmology & visual science, 2019. 60(1): p. 168-175.	A
23	Fard, M.A., et al., Quantification of peripapillary total retinal volume in pseudopapilledema and mild papilledema using spectral-domain optical coherence tomography. American journal of ophthalmology, 2014. 158(1): p. 136-43.	A
24	Flores-Rodríguez, P., P. Gili, and M.D. Martín-Ríos, Sensitivity and specificity of time-domain and spectral-domain optical coherence tomography in differentiating optic nerve head drusen and optic disc oedema. Ophthalmic Physiol Opt, 2012. 32(3): p. 213-21.	A
25	Gampa, A., et al., Quantitative Association Between Peripapillary Bruch's Membrane Shape and Intracranial Pressure. Investigative ophthalmology & visual science, 2017. 58(5): p. 2739-2745.	A

26	García-Montesinos, J., et al., Relationship between lamina cribrosa displacement and trans-laminar pressure difference in papilledema. Graefes Arch Clin Exp Ophthalmol, 2017. 255(6): p. 1237-1243.	A
27	Geddie, B.E., U.E. Altiparmak, and E.R. Eggenberger, Cup-to-disc ratio in patients with idiopathic intracranial hypertension is smaller than that in normal subjects. Journal of neuro-ophthalmology : the official journal of the North American Neuro-Ophthalmology Society, 2010. 30(3): p. 231-4.	A
28	Gozzi, F., et al., Optical coherence tomography in the differential diagnosis of true edema versus pseudoedema of the optic disc. Acta Ophthalmologica, 2017. 95.	A
29	Group, O.C.T.S.-S.C.f.N.I.I.H.S., et al., Baseline OCT measurements in the idiopathic intracranial hypertension treatment trial, part II: correlations and relationship to clinical features. Investigative ophthalmology & visual science, 2014. 55(12): p. 8173-9.	A
30	Hamill, E., et al., Cup-to-Disc Ratio in Idiopathic Intracranial Hypertension without Papilloedema. Neuro-ophthalmology (Aeolus Press), 2014. 38(2): p. 69-73.	A
31	Hata, M. and K. Miyamoto, Causes and Prognosis of Unilateral and Bilateral Optic Disc Swelling. Neuro-Ophthalmology, 2017. 41(4): p. 187-191.	A
32	Hata, M., et al., Measurement of retinal nerve fiber layer thickness in eyes with optic disc swelling by using scanning laser polarimetry and optical coherence tomography. Clinical ophthalmology (Auckland, N.Z.), 2014. 8: p. 105-11.	A
33	Hoye, V.J., 3rd, et al., Optical coherence tomography demonstrates subretinal macular edema from papilledema. Archives of ophthalmology (Chicago, Ill. : 1960), 2001. 119(9): p. 1287-90.	A
34	Huang-Link, Y., et al., Optical coherence tomography represents a sensitive and reliable tool for routine monitoring of idiopathic intracranial hypertension with and without papilledema. European journal of neurology, 2019. 26(5): p. 808-e57.	A
35	Huang-Link, Y.M., et al., OCT measurements of optic nerve head changes in idiopathic intracranial hypertension. Clinical Neurology and Neurosurgery, 2015. 130: p. 122-127.	A
36	Igarashi, N., et al., Optic disc cupping characteristics of normal pressure hydrocephalus patients with normal-tension glaucoma. Scientific reports, 2019. 9(1): p. 3108.	A
37	Intracranial, N.I., Baseline OCT Measurements in the Idiopathic Intracranial Hypertension Treatment Trial, Part I: Quality Control, Comparisons, and Variability (vol 55, pg 8180, 2014). Investigative Ophthalmology & Visual Science, 2016. 57(15): p. 6909-6909.	A
38	Johnson, L.N., et al., Differentiating optic disc edema from optic nerve head drusen on optical coherence tomography. Archives of ophthalmology (Chicago, Ill. : 1960), 2009. 127(1): p. 45-9.	A
39	Kamil, M.A.S., et al., Association between papilledema and guillian - Barre syndrome. Indian Journal of Public Health Research and Development, 2018. 9(12): p. 549-555.	A
40	Kaufhold, F., et al., Optic nerve head quantification in idiopathic intracranial hypertension by spectral domain OCT. PloS one, 2012. 7(5): p. e36965.	A

41	Kim, B.H. and E.J. Lee, Optic Disc Swelling After Intraocular Pressure Lowering Treatment in Acute Primary Angle Closure. Journal of glaucoma, 2017. 26(2): p. e87-e89.	A
42	Kim, M.J. and S.P. Kelly, Optical Coherence Tomography Imaging of Paton Folds in Papilledema With Retinopathy From Systemic Hypertension. JAMA ophthalmology, 2016. 134(10): p. e162121.	A
43	Koraysha, N.A., et al., Evaluating optic nerve diameter as a possible biomarker for disability in patients with multiple sclerosis. Neuropsychiatric Disease and Treatment, 2019. 15: p. 2571-2578.	A
44	Kupersmith, M., Baseline optical coherence tomography (OCT) measurements in the idiopathic intracranial hypertension treatment trial: Correlations and relationship to clinical features. Neurology, 2014. 82(10).	A
45	Kupersmith, M., Effects of intervention on optical imaging of papilledema in the idiopathic intracranial hypertension treatment trial. Neurology, 2015. 84.	A
46	Kupersmith, M., et al., Papilledema Outcomes from the Optical Coherence Tomography Substudy of the Idiopathic Intracranial Hypertension Treatment Trial. Ophthalmology, 2015. 122(9): p. 1939-1945.	A
47	Kupersmith, M.J. and N.I.S. Grp, Baseline Optical Coherence Tomography (OCT) of Participants in the Idiopathic Intracranial Hypertension Treatment Trial: Correlations and Relationships to Clinical Features. Investigative Ophthalmology & Visual Science, 2014. 55(13): p. 2.	A
48	Kupersmith, M.J., Baseline OCT measurements in the idiopathic intracranial hypertension treatment trial, part I: Quality control, comparisons, and variability. Investigative Ophthalmology and Visual Science, 2014. 55(12): p. 8180-8188.	A
49	Kupersmith, M.J., et al., Optical coherence tomography of the swollen optic nerve head: Deformation of the peripapillary retinal pigment epithelium layer in papilledema. Investigative Ophthalmology and Visual Science, 2011. 52(9): p. 6558-6564.	A
50	Kupersmith, M.J., et al., Papilledema Outcomes from the Optical Coherence Tomography Substudy of the Idiopathic Intracranial Hypertension Treatment Trial. Ophthalmology, 2015. 122(9): p. 1939-+.	A
51	Kupersmith, M.J., et al., Scanning laser polarimetry reveals status of RNFL integrity in eyes with optic nerve head swelling by OCT. Investigative ophthalmology & visual science, 2012. 53(4): p. 1962-70.	A
52	Kupersmith, M.J., Papilledema outcome evaluation in the OCT substudy of the idiopathic intracranial hypertension treatment trial. Investigative Ophthalmology and Visual Science, 2015. 56(7): p. 2235.	A
53	Labib, D.M. and D.H.A. Raouf, Diagnostic value of optical coherence tomography in patients with idiopathic intracranial hypertension. Egyptian Journal of Neurology Psychiatry and Neurosurgery, 2015. 52(4): p. 249-253.	A
54	Laemmer, R., et al., Detection of nerve fiber atrophy in apparently effectively treated papilledema in idiopathic intracranial hypertension. Graefes Archive for Clinical and Experimental Ophthalmology, 2010. 248(12): p. 1787-1793.	A
55	Lee, K.M., S.J. Woo, and J.-M. Hwang, Differentiation of optic nerve head drusen and optic disc edema with spectral-domain optical coherence tomography. Ophthalmology, 2011. 118(5): p. 971-7.	A

56	Liu, K.C., et al., Venous sinus stenting for reduction of intracranial pressure in IIH: A prospective pilot study. Journal of Neurosurgery, 2017. 127(5): p. 1126-1133.	A
57	Malhotra, K., et al., Association Between Peripapillary Bruch's Membrane Shape and Intracranial Pressure: Effect of Image Acquisition Pattern and Image Analysis Method, a Preliminary Study. Frontiers in neurology, 2018. 9: p. 1137.	A
58	Margolin, E., Diagnosis and grading of papilledema in patients with raised intracranial pressure using optical coherence tomography versus clinical expert assessment using a clinical staging scale. Evidence-Based Ophthalmology, 2011. 12(1): p. 42-43.	A
59	Marzoli, S.B., et al., Quantitative analysis of optic nerve damage in idiopathic intracranial hypertension (IIH) at diagnosis. Neurological sciences : official journal of the Italian Neurological Society and of the Italian Society of Clinical Neurophysiology, 2013. 34 Suppl 1: p. S143-5.	A
60	Monteiro, M.L. and C.L. Afonso, Macular thickness measurements with frequency domain-OCT for quantification of axonal loss in chronic papilledema from pseudotumor cerebri syndrome. Eye (Lond), 2014. 28(4): p. 390-8.	A
61	Oner, A., A. Agadayi, and N. Sinim, Optik Disk Odemi ve Optik Disk Druzeni Ayriminda Spektral-Domain Optik Kohorens Tomografi KullanilmasiDifferentiating optic disc edema from optic nerve head drusen with spectral domain optical coherence tomography. Retina-Vitreus, 2015. 23(2): p. 141-144.	A
62	Optical Coherence Tomography Substudy Committee; NORDIC Idiopathic Intracranial Hypertension Study Group. Papilledema Outcomes from the Optical Coherence Tomography Substudy of the Idiopathic Intracranial Hypertension Treatment Trial. Ophthalmology, 2015. 122(9): p. 1939-45.e2.	A
63	Optical Coherence Tomography Substudy, C. and N.I.I.H.S. Group, Papilledema Outcomes from the Optical Coherence Tomography Substudy of the Idiopathic Intracranial Hypertension Treatment Trial. Ophthalmology, 2015. 122(9): p. 1939-45.e2.	A
64	Pardon LP, Cheng H, Tang RA, Saenz R, Frishman LJ, Patel NB. Custom Optical Coherence Tomography Parameters for Distinguishing Papilledema from Pseudopapilledema. Optom Vis Sci. 2019;96(8):599-608. doi:10.1097/OPX.0000000000001408	A
65	Pardon, L.P., et al., Custom Optical Coherence Tomography Parameters for Distinguishing Papilledema from Pseudopapilledema. Optometry and vision science : official publication of the American Academy of Optometry, 2019. 96(8): p. 599-608.	A
66	Pasaoglu, I., et al., Lamina cribrosa surface position in idiopathic intracranial hypertension with swept-source optical coherence tomography. Indian journal of ophthalmology, 2019. 67(7): p. 1085-1088.	A
67	Patel, M.D., et al., Methods for Quantifying Optic Disc Volume and Peripapillary Deflection Volume Using Radial Optical Coherence Tomography Scans and Association With Intracranial Pressure. Frontiers in neurology, 2019. 10: p. 798.	A
68	Pilat, A.V., et al., Macular morphology in patients with optic nerve head drusen and optic disc edema. Ophthalmology, 2014. 121(2): p. 552-7.	A

69	Pilat, A.V., et al., Morphology of retinal vessels in patients with optic nerve head drusen and optic disc edema. Investigative ophthalmology & visual science, 2014. 55(6): p. 3484-90.	A
70	Rebolleda, G. and F.J. Muñoz-Negrete, Follow-up of mild papilledema in idiopathic intracranial hypertension with optical coherence tomography. Invest Ophthalmol Vis Sci, 2009. 50(11): p. 5197-200.	A
71	Rodriguez Torres Y, Lee P, Mihlstin M, Tomsak RL. Correlation Between Optic Disc Peripapillary Capillary Network and Papilledema Grading in Patients With Idiopathic Intracranial Hypertension: A Study of Optical Coherence Tomography Angiography [published online ahead of print, 2020 Jan 16]. J Neuroophthalmol. 2020;10.1097/WNO.0000000000000877. doi:10.1097/WNO.0000000000000877	A
72	Saenz, R., et al., Use of A-scan Ultrasound and Optical Coherence Tomography to Differentiate Papilledema From Pseudopapilledema. Optometry and vision science : official publication of the American Academy of Optometry, 2017. 94(12): p. 1081-1089.	A
73	Sarac, O., et al., Differentiation of optic disc edema from optic nerve head drusen with spectral-domain optical coherence tomography. Journal of neuro-ophthalmology : the official journal of the North American Neuro-Ophthalmology Society, 2012. 32(3): p. 207-11.	A
74	Savini, G., et al., Detection and quantification of retinal nerve fiber layer thickness in optic disc edema using stratus OCT. Archives of Ophthalmology, 2006. 124(8): p. 1111-1117.	A
75	Scott, C.J., et al., Diagnosis and grading of papilledema in patients with raised intracranial pressure using optical coherence tomography vs clinical expert assessment using a clinical staging scale. Archives of Ophthalmology, 2010. 128(6): p. 705-711.	A
76	Sheils, C.R., et al., The Relationship Between Optic Disc Volume, Area, and Frisen Score in Patients With Idiopathic Intracranial Hypertension. American journal of ophthalmology, 2018. 195: p. 101-109.	A
77	Sibony, P., et al., Optical coherence tomography shape analysis of the peripapillary retinal pigment epithelium layer in presumed optic nerve sheath meningiomas. Journal of neuro-ophthalmology : the official journal of the North American Neuro-Ophthalmology Society, 2014. 34(2): p. 130-6.	A
78	Sibony, P., M.J. Kupersmith, and F.J. Rohlf, Shape analysis of the peripapillary RPE layer in papilledema and ischemic optic neuropathy. Investigative ophthalmology & visual science, 2011. 52(11): p. 7987-95.	A
79	Sibony, P.A., et al., Retinal and Choroidal Folds in Papilledema. Investigative ophthalmology & visual science, 2015. 56(10): p. 5670-80.	A
80	Sibony, P.A., Gaze Evoked Deformations of the Peripapillary Retina in Papilledema and Ischemic Optic Neuropathy. Investigative ophthalmology & visual science, 2016. 57(11): p. 4979-4987.	A
81	Sinclair, A.J., et al., Low energy diet and intracranial pressure in women with idiopathic intracranial hypertension: prospective cohort study. BMJ (Clinical research ed.), 2010. 341: p. c2701.	A
82	Skau, M., et al., Disease activity in idiopathic intracranial hypertension: A 3-month follow-up study. Journal of Neurology, 2011. 258(2): p. 277-283.	A

83	Skau, M., et al., OCT for optic disc evaluation in idiopathic intracranial hypertension. Graefe's Archive for Clinical and Experimental Ophthalmology, 2011. 249(5): p. 723-730.	A
84	Smith, K.A., et al., A case series of dural venous sinus stenting in idiopathic intracranial hypertension: association of outcomes with optical coherence tomography¹. International Journal of Neuroscience, 2017. 127(2): p. 145-153.	A
85	Smith, S.V. and D.I. Friedman, The Idiopathic Intracranial Hypertension Treatment Trial: a Review of the Outcomes. Headache, 2017. (no pagination).	A
86	Starks, V., et al., Effect of optic nerve sheath fenestration for idiopathic intracranial hypertension on retinal nerve fiber layer thickness. Orbit (Amsterdam, Netherlands), 2016. 35(2): p. 87-90.	A
87	Steinegger, K., C. Bergin, and Y. Guex-Crosier, Malignant hypertension: clinical manifestations of 7 cases. Klinische Monatsblatter fur Augenheilkunde, 2015. 232(4): p. 590-2.	A
88	Tatar, I.T., et al., Morphological assessment of lamina cribrosa in idiopathic intracranial hypertension. Indian journal of ophthalmology, 2020. 68(1): p. 164-167.	A
89	Teismann, N., et al., Point-of-care ocular ultrasound to detect optic disc swelling. Academic Emergency Medicine, 2013. 20(9): p. 920-925.	A
90	Tirakotai, W., et al., Endoscopic Optic Nerve Sheath Fenestration for Treatment of Papilledema Secondary to Intracranial Venous Hypertension: Report of Two Cases. Journal of the Medical Association of Thailand = Chotmaihet thangphaet, 2016. 99 Suppl 3: p. S141-6.	A
91	Tsikata, E., et al., Volumetric Measurement of Optic Nerve Head Drusen Using Swept-Source Optical Coherence Tomography. Journal of Glaucoma, 2017. 26(9): p. 798-804.	A
92	Verma, R., et al., Ophthalmological manifestation in patients of tuberculous meningitis. QJM : monthly journal of the Association of Physicians, 2019. 112(6): p. 409-419.	A
93	Waisbourd, M., et al., OCT assessment of morphological changes of the optic nerve head and macula in idiopathic intracranial hypertension. Clinical neurology and neurosurgery, 2011. 113(10): p. 839-43.	A
94	Wall, M., et al., Threshold Static Automated Perimetry of the Full Visual Field in Idiopathic Intracranial Hypertension. Investigative Ophthalmology & Visual Science, 2019. 60(6): p. 1898-1905.	A
95	Wang, J.K., et al., Peripapillary retinal pigment epithelium layer shape changes from acetazolamide treatment in the idiopathic intracranial hypertension treatment trial. Investigative Ophthalmology and Visual Science, 2017. 58(5): p. 2554-2565.	A
96	Wang, J.K., et al., Three-dimensional bruch's membrane shape change over time with acetazolamide treatment in the idiopathic intracranial hypertension treatment trial (IIHTT). Investigative Ophthalmology and Visual Science, 2017. 58(8).	A
97	Wartak, A., et al., Investigating spontaneous retinal venous pulsation using Doppler optical coherence tomography. Scientific Reports, 2019. 9: p. 11.	A
98	Yri, H.M., et al., The course of headache in idiopathic intracranial hypertension: a 12-month prospective follow-up study. Eur J Neurol, 2014. 21(12): p. 1458-64.	A

99	Bahnasy, W.S., et al., Neuro-ophthalmological biomarkers of visual outcome in newly diagnosed idiopathic intracranial hypertension. Egyptian Journal of Neurology Psychiatry and Neurosurgery, 2019. 55(1): p. 8.	B
100	Casas, P., et al., Retinal nerve fibre layer evaluation using optical coherence tomography in patients with obstructive sleep apnea syndrome (OSAS). Neuro-Ophthalmology, 2011. 35.	B
101	Chang, Y.C., et al., Relationship Between Optic Nerve Protrusion Measured by OCT and MRI and Papilledema Severity. Invest Ophthalmol Vis Sci, 2015. 56(4): p. 2297-302.	B
102	Chen, J.J., et al., Causes and Prognosis of Visual Acuity Loss at the Time of Initial Presentation in Idiopathic Intracranial Hypertension. Investigative Ophthalmology & Visual Science, 2015. 56(6): p. 3850-3859.	B
103	Chen, Q., et al., Pseudotumour Cerebri Syndrome in China: A Cohort Study. Scientific reports, 2020. 10(1): p. 1222.	B
104	Figus, M., et al., Optical Coherence Tomography in Patients with Chiari I Malformation. Biomed Research International, 2015: p. 7.	B
105	Ghasemi Falavarjani K, Tian JJ, Akil H, Garcia GA, Sadda SR, Sadun AA. SWEPT-SOURCE OPTICAL COHERENCE TOMOGRAPHY ANGIOGRAPHY OF THE OPTIC DISK IN OPTIC NEUROPATHY. Retina. 2016;36 Suppl 1:S168-S177. doi:10.1097/IAE.0000000000001259	B
106	Goldhagen BE, Bhatti MT, Srinivasan PP, Chiu SJ, Farsiu S, El-Dairi MA. Retinal atrophy in eyes with resolved papilledema detected by optical coherence tomography. J Neuroophthalmol. 2015;35(2):122-126. doi:10.1097/WNO.0000000000000210	B
107	Goldhagen, B.E., et al., Retinal atrophy in eyes with resolved papilledema detected by optical coherence tomography. Journal of Neuro-Ophthalmology, 2015. 35(2): p. 122-123.	B
108	Ju, D.G., et al., Clinical Significance of Tumor-Related Edema of Optic Tract Affecting Visual Function in Patients with Sellar and Suprasellar Tumors. World Neurosurg, 2019. 132: p. e862-e868.	B
109	Karam, E.Z. and T.R. Hedges, Optical coherence tomography of the retinal nerve fibre layer in mild papilloedema and pseudopapilloedema. The British journal of ophthalmology, 2005. 89(3): p. 294-8.	B
110	Kasl, Z., et al., [The Current Diagnostic Possibilities and Cooperation of Oftalmologist and Neurologist Concerning in Patients with Idiopatic Intracranial Hypertension]. Soucasne moznosti oftalmologicke diagnostiky a spoluprace oftalmologa s neurologem u pacientu s idiopatickou intrakranialni hypertenzi., 2016. 72(2): p. 32-8.	B
111	Kim, M.S., et al., Morphologic Features of Buried Optic Disc Drusen on En Face Optical Coherence Tomography and Optical Coherence Tomography Angiography. American Journal of Ophthalmology, 2020. 213: p. 125-133.	B
112	Koktekir, E., et al., Resolution of papilledema after endoscopic third ventriculostomy versus cerebrospinal fluid shunting in hydrocephalus: A comparative study: Clinical article. Journal of Neurosurgery, 2014. 120(6): p. 1465-1470.	B
113	Kulkarni, K.M., et al., Differentiating mild papilledema and buried optic nerve head drusen using spectral domain optical coherence tomography. Ophthalmology, 2014. 121(4): p. 959-63.	B

114	Martinez, M.R. and A. Ophir, Optical coherence tomography as an adjunctive tool for diagnosing papilledema in young patients. Journal of pediatric ophthalmology and strabismus, 2011. 48(3): p. 174-81.	B
115	Merticariu, C.I., et al., Optical coherence tomography assessment of structural changes in the optic nerve head and peripapillary retina in idiopathic intracranial hypertension. Archives of the Balkan Medical Union, 2019. 54(2): p. 267-273.	B
116	Rizzo, J.L., et al., Perimetry, retinal nerve fiber layer thickness and papilledema grade after cerebrospinal fluid shunting in patients with idiopathic intracranial hypertension. Journal of neuro-ophthalmology : the official journal of the North American Neuro-Ophthalmology Society, 2015. 35(1): p. 22-5.	B
117	Sajjadi, F., et al., New predictive value of optical coherence tomography analysis in the diagnosis of idiopathic intracranial hypertension. Journal of Contemporary Medical Sciences, 2017. 3(10): p. 197-207.	B
118	Santos, M.A.K., et al., [Optic disc changes by optical coherence tomography in optic disc edema in Lomé]. J Fr Ophtalmol, 2017. 40(4): p. 314-318.	B
119	Sibony, P., et al., Effects of lowering cerebrospinal fluid pressure on the shape of the peripapillary retina in intracranial hypertension. Investigative Ophthalmology and Visual Science, 2014. 55(12): p. 8223-8231.	B
120	Tang, L., et al., Quantitative evaluation of papilledema from stereoscopic color fundus photographs. Investigative Ophthalmology and Visual Science, 2012. 53(8): p. 4490-4497.	B
121	Tang, Y., et al., Assessment of visual function by optical coherence tomography and visual field for craniopharyngioma patients. International Eye Science, 2015. 15(9): p. 1621-1624.	B
122	Tüntaş Bilen, F. and H. Atilla, Peripapillary Vessel Density Measured by Optical Coherence Tomography Angiography in Idiopathic Intracranial Hypertension. J Neuroophthalmol, 2019. 39(3): p. 319-323.	B
123	Vartin C, V., et al., Detection of mild papilloedema using spectral domain optical coherence tomography. The British journal of ophthalmology, 2012. 96(3): p. 375-9.	B
124	Wang, J.K., et al., Automated quantification of volumetric optic disc swelling in papilledema using spectral-domain optical coherence tomography. Investigative Ophthalmology and Visual Science, 2012. 53(7): p. 4069-4075.	B
125	Zhang, L., C. Sun, and X. Sun, [The clinical features and value of macular ganglion cell complex thickness patterns in patients with optic chiasma lesion]. Zhonghua Yan Ke Za Zhi, 2016. 52(5): p. 335-42.	B
126	Afonso, C., et al., Evaluation of inner retinal thicknesses in the macula of patients with chronic papilledema from pseudotumor cerebri syndrome using frequency domain OCT. Investigative Ophthalmology and Visual Science, 2013. 54(15).	C
127	Agne, J., et al., Automatic Detection of Folds and Wrinkles Due to Swelling of the Optic Disc, in Fetal, Infant and Ophthalmic Medical Image Analysis, M.J. Cardoso and T. Arbel, Editors. 2017, Springer International Publishing Ag: Cham. p. 235-242.	C
128	Agne, J., et al., Determining Degree of Optic Nerve Edema from Color Fundus Photography, in Medical Imaging 2015: Computer-Aided	C

	Diagnosis, L.M. Hadjiiski and G.D. Tourassi, Editors. 2015, Spie-Int Soc Optical Engineering: Bellingham.	
129	Agne, J., et al., Monitoring Intracranial Pressure By a Semi-automated Determination of Bruch's Membrane Deformation From Routine SD-OCT Volume Scans of the Optic Disc. Investigative Ophthalmology & Visual Science, 2016. 57(12): p. 3.	C
130	Chang, L., et al., Optical coherence tomography in the evaluation of neurofibromatosis type 1 subjects with optic pathway gliomas. Journal of AAPOS, 2010. 14(1).	C
131	Chang, L., et al., Optical coherence tomography in the evaluation of neurofibromatosis type 1 subjects with optic pathway gliomas. Journal of AAPOS, 2010. 14(1).	C
132	Chang, M., et al., Accuracy of ultrasonography, fundus photography, autofluorescence (AF), fluorescein angiography (FA), and optical coherence tomography (OCT) in differentiating pseudopapilledema from true optic disk edema (ODE) in children. Journal of AAPOS, 2017. 21(4).	C
133	Christodoulou, L., et al., Review of the pathway for children with '? Swollen' optic discs; A service evaluation and quality improvement study. Developmental Medicine and Child Neurology, 2019. 61: p. 92.	C
134	Cohen, Y., et al., OCT imaging of papilledema in pediatric idiopathic intracranial hypertension. Investigative Ophthalmology and Visual Science, 2016. 57(12): p. 4546.	C
135	Dahlmann-Noor, A.H., et al., Ultrasonography, OCT and OCT-angiography in the diagnostic workup of children with suspected papilledema. Investigative Ophthalmology and Visual Science, 2017. 58(8).	C
136	D'Antona, L., et al., Spontaneous retinal venous pulsation: Towards non-invasive assessment of ICP. Fluids and Barriers of the CNS, 2018. 15.	C
137	D'Antona, L., et al., Spontaneous retinal venous pulsation: Towards noninvasive assessment of intracranial pressure. British Journal of Neurosurgery, 2018. 32(3): p. 336.	C
138	Duman, R., et al., Evaluation of ganglion cell layer and nerve fibre layer changes in patients with idiopathic intracranial hypertension. Neuro-Ophthalmology, 2015. 39.	C
139	Falavarjani, K.G., et al., Swept-source optical coherence tomography angiography of the optic disk in optic neuropathy. Retina, 2016. 36.	C
140	Fell, D., et al., Perfused large vessel and capillary densities in various grades of papilledema using OCTA custom software. Investigative Ophthalmology and Visual Science, 2017. 58(8).	C
141	Gelener, P., et al., Optic coherence tomography for optic disc and clinical evaluation in idiopathic intracranial hypertension. Journal of the Neurological Sciences, 2013. 333.	C
142	Gobuty, M., et al., Optical coherence tomography (OCT) use in assessing hydrocephalus. Investigative Ophthalmology and Visual Science, 2014. 55(13): p. 3362.	C
143	Gronlund, M.A., L. Mybeck, and S. Andersson, Imaging of the retina and optic nerve using optical coherence tomography in adolescents with surgically treated hydrocephalus. European Journal of Ophthalmology, 2017. 27(5).	C
144	Gupta, A., et al., First Peak fractal analysis of optical coherence tomography angiography in eyes with papilledema. Investigative Ophthalmology and Visual Science, 2018. 59(9).	C

145	Hacker, M., et al., Segmentation of the surfaces of the retinal layer from OCT images. Medical image computing and computer-assisted intervention : MICCAI ... International Conference on Medical Image Computing and Computer-Assisted Intervention, 2006. 9: p. 800-807.	C
146	Hajjar, D., P. Pujol, and G. Julio, Optical coherence tomography (OCT) utility in diagnosis and follow-up of papilloedema. Neuro-Ophthalmology, 2011. 35.	C
147	Homma, T.K., et al., Optical coherence tomography (OCT) as a tool to identify idiopathic intracranial hypertension in pediatric patients under GH therapy. Endocrine Reviews, 2014. 35.	C
148	Ioyleva, E. and E. Kabanova, OCT- Angiography: Perspectives of differential diagnostics of pathology optic nerve. Acta Ophthalmologica, 2018. 96: p. 55.	C
149	Ioyleva, E., E. Kabanova, and M. Krivosheeva, Measurement of macular ganglion cell-innerplexiform layer with spectral-domain optical coherence tomography in patients with optic nerve head drusen and papilledema. Acta Ophthalmologica, 2018. 96: p. 55.	C
150	Islam, M.S., et al., Deep learning based retinal blood vessel segmentation of multiple optical coherence tomography en-face images in cases of optic disc swelling. Investigative Ophthalmology and Visual Science, 2019. 60(9).	C
151	Iwase, T., et al., Serum level of vascular endothelial growth factor and opening lumbar pressure significantly correlated with optic disc edema in patients with POEMS syndrome. Investigative Ophthalmology and Visual Science, 2015. 56(7): p. 3880.	C
152	Jenkins, K.S., C.J. Layton, and M.K.M. Adams, Ophthalmoscopic and video OCT methods to detect spontaneous venous pulsation in individuals with apparently normal intracranial pressure: The rebirth of the SVP? Acta Ophthalmologica, 2017. 95.	C
153	Jensen, R. and M. Skau, Disease activity in idiopathic intracranial hypertension: A 3-month follow-up study. Journal of Headache and Pain, 2010. 11.	C
154	Jiramongkolchai, K., et al., Longitudinal Follow-Up of Papilledema by Optical Coherence Tomography Macular Segmentation. Investigative Ophthalmology & Visual Science, 2016. 57(12): p. 2.	C
155	Kapila, A., V. Lal, and A. Gupta, Clinical and neuro-ophthalmologic predictors of visual outcome in idiopathic intracranial hypertension (IIH). Journal of the Neurological Sciences, 2015. 357.	C
156	Katz, S.E., A.M. Mahmoud, and C.J. Roberts, Objective quantification of papilledema resolution after optic nerve sheath fenestration. Investigative Ophthalmology and Visual Science, 2017. 58(8).	C
157	Kedar, S., et al., Effects of acute intracranial pressure change on human and pig optic nerve head using optical coherence tomography. Investigative Ophthalmology & Visual Science, 2017. 58(8): p. 3.	C
158	Kee, H.J., S.J. Woo, and J.M. Hwang, Longitudinal change of buried optic disc drusen in children. Journal of AAPOS, 2019. 23(4).	C
159	Krauss, H.R., et al., The clinical utility of ocular coherence tomography in evaluation and management of skull base disorders. Journal of Neurological Surgery Part B: Skull Base, 2017. 78.	C

160	Krivosheeva, M., E. Ioyleva, and E. Kabanova, Measurement of macula thickness and macular ganglion cell with SS-OCT in patients with mild papille. Ophthalmic Research, 2018. 60: p. 14.	C
161	Kupersmith, M., R. Kardon, and M. Durbin, Retinal nerve fiber layer alteration differences between OCT and scanning laser polarimetry with optic nerve head swelling. Neurology, 2012. 78(1).	C
162	Lascaratos, G. and S. Madill, Patients With Idiopathic Intracranial Hypertension Have Significantly Larger Optic Nerve Heads Than Controls. Investigative Ophthalmology & Visual Science, 2010. 51(13): p. 2.	C
163	Lenaghan, P., et al., Point-of-care ocular sonography to detect optic disc swelling. Annals of Emergency Medicine, 2011. 58(4).	C
164	Malem, A., S. West, and G. De Salvo, Multicolor imaging in the assessment and diagnosis of optic disc swelling. Investigative Ophthalmology and Visual Science, 2015. 56(7): p. 2238.	C
165	Martinez-Alvarez, L., et al., Peripapillary choroidal neovascularization in papilloedema: Report of two cases and review of the literature. Neuro-Ophthalmology, 2015. 39.	C
166	McHugh, J., et al., Use of the heidelberg spectralis oct device to assess spontaneous venous pulsation in patients with headaches. Neuro-Ophthalmology, 2017. 41.	C
167	McNabb, R.P., et al., Widefield whole eye ssoct measurement of retinal curvature for screening of elevated intracranial pressure. Investigative Ophthalmology and Visual Science, 2018. 59(9).	C
168	Miller, E.R., C.O. Jordan, and D.L. Rogers, Optic nerve optical coherence tomography versus humphrey visual field analysis in pediatric intracranial hypertension. Journal of AAPOS, 2014. 18(4).	C
169	Miller, J.W., et al., Differentiation between papilledema and nonarteritic anterior ischemic optic neuropathy using retinal layer shape and regional volume features in spectral-domain optical coherence tomography. Investigative Ophthalmology and Visual Science, 2018. 59(9).	C
170	Monroy, J.E.G., et al., Optic nerve volumetric measurements in pediatric papilledema with spectral domain OCT. Journal of AAPOS, 2014. 18(4).	C
171	Morrow, M., F. Villanueva, and F. Abukhalil, Evolution of macular findings on optical coherence tomography (OCT) in papilledema. Neurology, 2014. 82(10).	C
172	Morrow, M., The relationship between optical coherence tomographic (OCT) and perimetric findings in patients with papilledema. Neurology, 2014. 82(10).	C
173	Morrow, M., The relationship between optical coherence tomographic and perimetric findings in patients with papilledema. Journal of the Neurological Sciences, 2013. 333.	C
174	Moss, H., et al., Optic nerve head geometry as a function of chronic intracranial pressure. Investigative Ophthalmology and Visual Science, 2017. 58(8).	C
175	Moss, H., J.J. McAnany, and J.C. Park, Structure function relationships in idiopathic intracranial hypertension. Investigative Ophthalmology and Visual Science, 2015. 56(7): p. 2230.	C
176	Nayfach, J., et al., Integrating funduscopy, optical coherence tomography (OCT), and ultrasound to differentiate optic disc edema (ODE) from pseudo optic disc edema (PODE). Investigative Ophthalmology and Visual Science, 2016. 57(12): p. 4541.	C

177	Pak, K., et al., Anatomical and functional changes of optic disc and retinal nerve fiber layer after bariatric surgery in morbidly obese Korean patients with lower BMI. Investigative Ophthalmology and Visual Science, 2019. 60(9).	C
178	Parhiz, A.T., P. Sibony, and M. Kupersmith, SD-OCT in monitoring intracranial pressure in patients with CSF shunts. Investigative Ophthalmology and Visual Science, 2013. 54(15).	C
179	Pascoe, E., et al., A case series of intracranial hypertension in patients with Turner syndrome, with and without growth hormone therapy. International Journal of Pediatric Endocrinology, 2015. 2015.	C
180	Pathmanathan, N., et al., Audit of intracranial pressure monitoring in traumatic brain injury. Intensive care medicine, 2013. 39: p. S259-.	C
181	Pemp, B., et al., Changes of choroidal thickness as measured with spectral domain OCT in patients with idiopathic intracranial hypertension after lowering of intracranial pressure. Investigative Ophthalmology and Visual Science, 2013. 54(15).	C
182	Pemp, B., et al., Measurements by spectral domain oct in papilledema are associated with reduced retinal nerve fiber layer thickness after resolution of optic Disc swelling. Investigative Ophthalmology and Visual Science, 2014. 55(13): p. 5779.	C
183	Pemp, B., et al., The course of divergence paresis and papilloedema in patients with increased intracranial pressure. Neuro-Ophthalmology, 2015. 39.	C
184	Permeswaran, Y.M., et al., An en-face OCT registration approach for locating the center of bruch's membrane opening in longitudinal papilledema cases. Investigative Ophthalmology and Visual Science, 2019. 60(9).	C
185	Pilat, A., et al., Macula structure in patients with optic disc oedema and optic nerve head drusen. Investigative Ophthalmology and Visual Science, 2013. 54(15).	C
186	Reitner, A., B. Pemp, and K. Kircher, Retinal nerve fibre layer thickness measurement using spectral domain OCT in various optic nerve lesions. Neuro-Ophthalmology, 2011. 35.	C
187	Sarrafpour, S., et al., Fractal analysis of peripapillary vasculature in eyes with papilledema using optical coherence tomography angiography. Investigative Ophthalmology and Visual Science, 2017. 58(8).	C
188	Scott, L.L., et al., Correlation of opening pressure and Frisen grade of papilledema in pediatric patients with intracranial hypertension. Investigative Ophthalmology and Visual Science, 2015. 56(7): p. 2231.	C
189	Skau, M., et al., Diagnostic value of optical coherence tomography for intracranial pressure in idiopathic intracranial hypertension. Journal of Headache and Pain, 2013. 14(1).	C
190	Stark, R.M., et al., Assessment of intracranial pressure during venous sinus stenting. Journal of Neurological Surgery, Part B: Skull Base, 2016. 77.	C
191	Starke, R.M., et al., A prospective pilot study of intraparenchymal & intravenous cerebral pressure assessment during venous sinus stenting. Journal of Neurosurgery, 2016. 124(4).	C
192	Stroet, A., et al., Optical coherence tomography in idiopathic intracranial hypertension-first experiences. Neuropediatrics, 2012. 43(2).	C
193	Su, Q.Y., et al., Spectral-Domain Optical Coherence Tomography Optic-Nerve-Head and Macular En-Face Image Registration in Cases of	C

	Papilledema. Investigative Ophthalmology & Visual Science, 2017. 58(8): p. 3.	
194	Swinkin, E., A. Sundaram, and J.M. Pejman, Unilateral papilledema in idiopathic intracranial hypertension: A report of two cases and literature review. Neurology, 2017. 88(16).	C
195	Tang, R., et al., Optical coherence tomography visualization of optic nerve head structure detects acute changes in the intracranial pressure. Neurology, 2014. 82(10).	C
196	Thompson, D.A., et al., Outsized paediatric VEPs. Documenta Ophthalmologica, 2017. 135(1): p. 24.	C
197	Van De Beeten, S.D.C., et al., Increased total retinal thickness on OCT: A precursor for intracranial hypertension? Child's Nervous System, 2018. 34(5): p. 1006-1007.	C
198	Vangipuram, G. and H. Moss, Changes in basement membrane opening displacement within 1 hour following intracranial pressure lowering in subjects with and without idiopathic intracranial hypertension. Investigative Ophthalmology and Visual Science, 2015. 56(7): p. 2232.	C
199	Varma, H., et al., Longitudinal analysis of optic nerve edema, retinal nerve fiber layer thickness, and visual field mean deviation in pediatric patients with intracranial hypertension. Journal of AAPOS, 2019. 23(4).	C
200	Wall, M., et al., Detection of visual loss in IIH with static automated perimetry: Temporal wedge defects. Investigative Ophthalmology and Visual Science, 2019. 60(9).	C
201	Wan, M.J., Correlation between spectral domain optical coherence tomography (SD-OCT) and long-term vision loss in children with papilledema. Journal of AAPOS, 2019. 23(4).	C
202	Wang, J.K., et al., Automated 3-D Region-Based Volumetric Estimation of Optic Disc Swelling in Papilledema Using Spectral-Domain Optical Coherence Tomography, in Medical Imaging 2013: Biomedical Applications in Molecular, Structural, and Functional Imaging, J.B. Weaver and R.C. Molthen, Editors. 2013, Spie-Int Soc Optical Engineering: Bellingham.	C
203	Wang, J.K., et al., Automated Classification of Retinal Folds and Wrinkles in En-Face Optical Coherence Tomography Images with Optic Disc Swelling. Investigative ophthalmology & visual science, 2019. 60(9).	C
204	Wang, J.K., et al., Automated detection of retinal folds in papilledema using en-face spectral-domain optical coherence tomography. Investigative Ophthalmology and Visual Science, 2018. 59(9).	C
205	Wang, J.K., et al., Change in the deflection of the neural canal opening over time with acetazolamide treatment in idiopathic intracranial hypertension. Investigative Ophthalmology and Visual Science, 2015. 56(7): p. 2233.	C
206	Wang, J.K., et al., Regional spectral-domain optical coherence tomography features better predict Frisen scale grades than total volume alone in papilledema. Investigative Ophthalmology and Visual Science, 2013. 54(15).	C
207	Wang, J.K., et al., Semi-Automated 2D Bruch's Membrane Shape Analysis in Papilledema Using Spectral-Domain Optical Coherence Tomography, in Medical Imaging 2015: Biomedical Applications in Molecular, Structural, and Functional Imaging, B. Gimi and R.C. Molthen, Editors. 2015, Spie-Int Soc Optical Engineering: Bellingham.	C

208	Wang, J.K., R.H. Kardon, and M.K. Garvin, Combined Use of High-Definition and Volumetric Optical Coherence Tomography for the Segmentation of Neural Canal Opening in Cases of Optic Nerve Edema, in Medical Imaging 2015: Image Processing, S. Ourselin and M.A. Styner, Editors. 2015, Spie-Int Soc Optical Engineering: Bellingham.	C
209	Weaver, M.A., et al., Deformation of the peripapillary retinal pigmented epithelium layer on OCT: a useful tool to identify intracranial hypertension in a pediatric population? Journal of AAPOS, 2019. 23(4).	C
210	Yri, H., et al., EHMTI-0155. The course of headache in idiopathic intracranial hypertension: A 12 month prospective follow-up study. Journal of Headache and Pain, 2014. 15.	C
211	Yri, H., et al., Idiopathic intracranial hypertension is not benign; a prospective long-term follow-up study. Journal of Headache and Pain, 2013. 14(1).	C
212	Kleinberg, T.T. and J.R. Bilyk, Evolution of Disc Edema in Thyroid-Related Compressive Optic Neuropathy. Ophthalmic plastic and reconstructive surgery, 2016. 32(4): p. e100.	D
213	Perez-Lopez, M., D.S. Ting, and L. Clarke, Lamina cribrosa displacement after optic nerve sheath fenestration in idiopathic intracranial hypertension: a new tool for monitoring changes in intracranial pressure? Br J Ophthalmol, 2014. 98(11): p. 1603-4.	D
214	Rezkallah, A., et al., Hypertensive choroidopathy: Multimodal imaging and the contribution of wide-field swept-source oct-angiography. American journal of ophthalmology case reports, 2019. 13: p. 131-135.	D
215	Santos-Bueso, E., et al., [Idiopathic intracranial hypertension. Analysis by macular segmentation]. An Pediatr (Barc), 2015. 82(5): p. e230-3.	D
216	Shuaib, M.M., et al., Optical coherence tomography of the optic nerve head before and after optic nerve sheath fenestration for idiopathic intracranial hypertension. Acta neurologica Belgica, 2020.	D
217	Sibony, P.A. and M.J. Kupersmith, Paton's Folds revisited: Wrinkles, folds and creases in papilledema. Investigative Ophthalmology and Visual Science, 2016. 57(12): p. 4553.	D
218	Fard, M.A., Bruch's membrane opening on optical coherence tomography in pediatric papilledema and pseudopapilledema. Journal of AAPOS : the official publication of the American Association for Pediatric Ophthalmology and Strabismus, 2018. 22(3): p. 247.	E
219	García-Montesinos, J., et al., Bruch's membrane opening on optical coherence tomography in pediatric papilledema and pseudopapilledema. J aapos, 2018. 22(3): p. 247-248.	E
220	Ophir, A., Optical coherence tomography as an adjunctive tool for diagnosing pediatric pseudotumor cerebri. Journal of AAPOS, 2008. 12(4): p. 421-422.	E
221	Savini, G., et al., Optical coherence tomography for optic disc edema. Archives of Ophthalmology, 2011. 129(9): p. 1245-1246.	E

Rufai SR, *et al. BMJ Open* 2021; 11:e046935. doi: 10.1136/bmjopen-2020-046935
