# Supplementary material for: Detection of intracranial hypertension in children using optical coherence tomography: a systematic review
Source: BMJ Open. 2021 Aug 11;11(8):e046935. doi: 10.1136/bmjopen-2020-046935 (PMC8359522; doi:10.1136/bmjopen-2020-046935)
Supplement: Author's manuscript [file bmjopen-2020-046935.draft_revisions.pdf]

BMJ Open is committed to open peer review. As part of this commitment we make the peer review history of every article we publish publicly available.

When an article is published we post the peer reviewers' comments and the authors' responses online. We also post the versions of the paper that were used during peer review. These are the versions that the peer review comments apply to.

The versions of the paper that follow are the versions that were submitted during the peer review process. They are not the versions of record or the final published versions. They should not be cited or distributed as the published version of this manuscript.

BMJ Open is an open access journal and the full, final, typeset and author-corrected version of record of the manuscript is available on our site with no access controls, subscription charges or pay-per-view fees (http://bmjopen.bmj.com).

If you have any questions on BMJ Open's open peer review process please email info.bmjopen@bmj.com

BMJ Open

Optical Coherence Tomography and Paediatric Intracranial Hypertension: A Systematic Review

Journal:	*BMJ Open*
Manuscript ID	bmjopen-2020-046935
Article Type:	Original research
Date Submitted by the Author:	14-Nov-2020
Complete List of Authors:	Rufai, Sohaib; University of Leicester, Ulverscroft Eye Unit; Great Ormond Street Hospital for Children, Craniofacial Unit<br>Hisaund, Michael; University of Leicester, Neuroscience, Psychology and Behaviour<br>Jeelani, Noor ul Owase; Great Ormond Street Hospital for Children, Craniofacial Unit<br>McLean, Rebecca; University of Leicester, Ulverscroft Eye Unit
Keywords:	Neuro-ophthalmology < OPHTHALMOLOGY, Paediatric ophthalmology < OPHTHALMOLOGY, Paediatric neurology < NEUROLOGY, Neurological oncology < NEUROLOGY, Paediatric neurosurgery < PAEDIATRIC SURGERY, PLASTIC & RECONSTRUCTIVE SURGERY

SCHOLARONE™
Manuscripts

I, the Submitting Author has the right to grant and does grant on behalf of all authors of the Work (as defined in the below author licence), an exclusive licence and/or a non-exclusive licence for contributions from authors who are: i) UK Crown employees; ii) where BMJ has agreed a CC-BY licence shall apply, and/or iii) in accordance with the terms applicable for US Federal Government officers or employees acting as part of their official duties; on a worldwide, perpetual, irrevocable, royalty-free basis to BMJ Publishing Group Ltd ("BMJ") its licensees and where the relevant Journal is co-owned by BMJ to the co-owners of the Journal, to publish the Work in this journal and any other BMJ products and to exploit all rights, as set out in our licence.

The Submitting Author accepts and understands that any supply made under these terms is made by BMJ to the Submitting Author unless you are acting as an employee on behalf of your employer or a postgraduate student of an affiliated institution which is paying any applicable article publishing charge ("APC") for Open Access articles. Where the Submitting Author wishes to make the Work available on an Open Access basis (and intends to pay the relevant APC), the terms of reuse of such Open Access shall be governed by a Creative Commons licence – details of these licences and which Creative Commons licence will apply to this Work are set out in our licence referred to above.

Other than as permitted in any relevant BMJ Author's Self Archiving Policies, I confirm this Work has not been accepted for publication elsewhere, is not being considered for publication elsewhere and does not duplicate material already published. I confirm all authors consent to publication of this Work and authorise the granting of this licence.

For peer review only

Title Page

Title: Optical Coherence Tomography and Paediatric Intracranial Hypertension: A Systematic Review

Authors:

Dr Sohaib R. Rufai[1-3] (BMBS, MRes), Mr Michael Hisaund[1] (BMedSci), Dr Noor ul Owase Jeelani[3] (MPhil, FRCS), Dr Rebecca J. McLean[1] (MSc, PhD)

Affiliations:

1. The University of Leicester Ulverscroft Eye Unit, Robert Kilpatrick Clinical Sciences Building, Leicester Royal Infirmary, Leicester, UK.
2. Clinical and Academic Department of Ophthalmology, Great Ormond Street Hospital for Children NHS Foundation Trust, London, UK.
3. UCL Great Ormond Street Institute of Child Health, London, UK & Craniofacial Unit, Great Ormond Street Hospital for Children NHS Foundation Trust, London, WC1N 3JH, United Kingdom

Corresponding Author: Dr Rebecca J. McLean (MSc, PhD); Address: The University of Leicester Ulverscroft Eye Unit, Robert Kilpatrick Clinical Sciences Building, PO Box 65, Leicester LE2 7LX, UK; E-mail: rjm19@leicester.ac.uk; Telephone: +44 (0)116 2523152; Fax: +44 (0)116 2231996

Keywords: Intracranial pressure; paediatric intracranial hypertension; optical coherence tomography; early recognition; systematic review.

Funding: The Medical Research Council (London, UK) (grant no.: MR/N004566/1). SRR is funded by a National Institute for Health Research (NIHR) Doctoral Fellowship for this research project. This paper presents independent research funded by the National Institute for Health Research (NIHR) and MRC. The views expressed are those of the author(s) and not necessarily those of the MRC, the NHS, the NIHR or the Department of Health and Social Care.

Competing interests: The authors have no proprietary or commercial interest in any materials discussed in this study.

For peer review only

Abstract

Objectives: To evaluate the diagnostic capability of optical coherence tomography (OCT) in children aged under 18 years old with intracranial hypertension (IH); to provide recommendations for specialists based on published evidence.

Design: Systematic review

Methods: We conducted a systematic review using the following platforms to search the keywords 'optical coherence tomography' and 'intracranial hypertension' from inception until 2nd April 2020: CENTRAL, EMBASE, MEDLINE, PubMed and Web of Science, without language restrictions. Our search returned 2729 records, screened by two independent screeners. Studies were graded according to the Oxford Centre for Evidence-Based Medicine and National Institutes of Health Quality Assessment Tool for observational studies.

Results: 21 studies were included. Conditions included craniosynostosis (n=354 patients), idiopathic intracranial hypertension (IIH; n=102), space occupying lesion (SOL; n=42) and other pathology (n=29). OCT measures included optic nerve parameters, rim parameters (notably retinal nerve fibre layer thickness) and retinal parameters. Levels of evidence included 2b (n=13 studies), 3b (n=4) and 4 (n=4). Quality of 10 studies was fair and 11 poor. There was inconsistency in OCT parameters and gold standard measures studied, albeit OCT did demonstrate good diagnostic capability for IH in craniosynostosis, IIH and SOL.

Conclusions: This systematic review identified various evidence supporting the use of OCT in paediatric IH. This review recommends that OCT can be used to assist diagnosis and management of IH in children with craniosynostosis, IIH and SOL, in conjunction with established clinical measures of ICP. Further research is required to validate optimal OCT parameters in this role and to develop formal clinical guidelines.

PROSPERO registration number: CRD42019154254

Strengths and limitations of this study

- This is the first systematic review to evaluate the role of optical coherence tomography (OCT) in paediatric intracranial hypertension (IH).
- This systematic review was conducted in accordance with rigorous Cochrane methodology.
- A broad search strategy was employed, without date or language restrictions, to identify all relevant evidence.
- There was inconsistency in evidence levels and quality across the included studies, albeit OCT did demonstrate good diagnostic capability for paediatric IH.
- Validating prospective studies are required to develop formal clinical guidelines for OCT in paediatric IH.

For peer review only - http://bmjopen.bmj.com/site/about/guidelines.xhtml

Text

Introduction

Intracranial hypertension (IH) was first described by Quincke in 1896 and remains a subject of major clinical importance.[1] IH affects between 0.63 and 0.71 per 100,000 children.[2, 3] Unaddressed IH can inflict devastating sequelae including visual impairment, neurocognitive delay, disability and death.[4, 5] Subacute pathology in children can cause insidious IH which may pose deleterious effects on the brain and vision before clinical detection. Thus, prompt detection and timely intervention is key in preventing or limiting the sequelae of IH.

Assessment of intracranial pressure (ICP) in children is notoriously difficult. Direct intraparenchymal measurement represents the gold standard, but carries numerous disadvantages including the need for overnight hospital admission, general anaesthesia and significant risk.[6, 7] An ideal screening method would be sensitive, specific, safe, highly reproducible, rapid, non-invasive and child friendly with the capability to record serial measurements. Existing measures fail to satisfy all these criteria and often yield equivocal results in young children, including fundus examination[8], B-scan ocular ultrasound[9, 10], radiology[11] and visual evoked potentials.[12, 13]

IH causes optic nerve and retinal changes, which can be detected and quantified using optical coherence tomography (OCT) – a non-invasive imaging method to acquire ultra-high resolution cross-sectional images of the optic nerve and retina within seconds.[14] OCT has been successfully used to study the normal and abnormal development of the optic nerve[15] and fovea[16] in children, plus various conditions associated with IH.[17-23] Here, we conducted a systematic review to assess the role of OCT in detecting IH in children and to make recommendations to specialists.

For peer review only

For peer review only - http://bmjopen.bmj.com/site/about/guidelines.xhtml

Methods

This systematic review was conducted in accordance with PRISMA guidelines[24] and the Cochrane Handbook.[25] The protocol is registered on PROSPERO[26] (CRD42019154254) and published in *BMJ Open*[27].

Eligibility criteria for considering studies for this review

Eligibility criteria were established *a priori* and included OCT studies of children (aged under 18 years) with intracranial hypertension. Level 4 evidence and above was included, as per the Oxford Centre for Evidence-based Medicine (CEBM).[28] Exclusion criteria were : i) adult studies aged 18 or over; ii) studies not pertaining to IH; iii) studies not using OCT; iv) case reports and expert opinion without critical appraisal.

Search methods for identifying studies

Medical subject headings (MeSH) terms for 'optical coherence tomography' and 'intracranial hypertension' were entered into search platforms: Cochrane Central Register of Controlled Trials (CENTRAL), MEDLINE, EMBASE, PubMed and Web of Science. Appendix 1 contains full details of our search terms and strategy. EndNote X9 (Thomson Reuters, New York, NY) software was used to manage data. No date/language restrictions were stipulated.

Study selection

A three-stage, independent screening process was employed by two screeners (SRR and RJM), involving eligibility screening of titles, abstracts and full papers. Screening questions are listed in Appendix 2.

Data collection and quality assessment

The main outcome measure was the diagnostic capability for OCT in detecting IH, expressed as diagnostic accuracy or by appropriate statistical testing.

Secondary outcome measures were:

- Condition(s) associated with IH per study;
- OCT device(s) used;
- OCT success rate;
- Other surrogate estimates of ICP;
- ICP range determined as normal.

Our data extraction tool was adapted from the Cochrane Collaboration (Appendix 3).[29] Evidence levels were graded by two independent graders (SRR and MH) as per the Oxford CEBM[28] and the National Institutes of Health (NIH) Quality Assessment Tool for Observational Studies[30] was applied for individual study quality grading (Appendix 4).

For peer review only

Patient and public involvement

There was no patient and public involvement specific to this systematic review. However, this group has commenced prospective research using handheld OCT in paediatric IH featuring substantial patient and public involvement, which will be reported separately to this systematic review.

For peer review only

For peer review only - http://bmjopen.bmj.com/site/about/guidelines.xhtml

Results

Our search was executed on April 2nd 2020 and data extraction completed on 12th July 2020. Our search returned 2729 records in total, 1665 following de-duplication. Following full-text screening, 21 studies were eligible for inclusion in our review (Figure 1). Supplementary Table 1 contains the list of excluded articles with reasons. Table 1 summarises the study characteristics and quality assessment of the 21 included studies. One study[31] was written in Polish and translated by an interpreter, whilst the remaining 20 were written in English. Nine studies were prospective[22, 23, 32-38] while twelve were retrospective.[31, 39-49] No randomised controlled trials (RCTs) or systematic reviews were eligible for inclusion. Following review of the included studies, it was deemed inappropriate to perform quantitative synthesis due to inconsistency in study design and methodology, particularly OCT parameters and gold standard measures of ICP. Therefore, qualitative synthesis was performed.

This review identified studies of craniosynostosis (n=354 patients), idiopathic intracranial hypertension (IIH; n=102), space occupying lesion (SOL; n=42) and other pathologies (n=29) associated with risk of IH. OCT measures in these studies included optic nerve parameters, rim parameters and retinal parameters. Main outcome measures are displayed in Table 2. Secondary outcome measures are reported per condition.

Evidence summary: craniosynostosis

Craniosynostosis is characterised by the premature, pathological fusion of one or more cranial sutures. This restriction in skull growth can cause IH. Six studies in this review utilised OCT in patients with craniosynostosis.[22, 23, 33, 34, 40, 49] These studies included a total of 393 participants, of which 354 were diagnosed with craniosynostosis[22, 23, 33, 34, 40, 49]. In one comparative study[22], 5 positive controls had hydrocephalus and suspected IH[22], while 34 were normal controls.[22] OCT devices used include the iVue[22, 23] (Optovue, Fremont, CA, US; software version 3.2) and Spectralis[33, 34, 40, 49] (Heidelberg Engineering, GmbH, Heidelberg, Germany).

OCT parameters evaluated include retinal nerve fibre layer (RNFL) thickness[22, 23, 40], retinal thickness[22, 23, 33, 34, 49] and anterior retinal projection.[22, 23] OCT parameters demonstrated good diagnostic capability for IH in craniosynostosis (Table 2). Increased RNFL thickness, retinal thickness and anterior retinal projection were associated with papilloedema, while RNFL thinning was associated with optic atrophy. Using single, on-table ICP measures, Swanson *et al*[22] demonstrated good sensitivity (89%) and specificity (62%) of combined RNFL thickness and maximal anterior retinal projection in detecting IH (Figure 2). They found that maximal RNFL thickness exceeding 207µm or maximal anterior retinal projection exceeding 159µm in either eye corresponded to the 97.5th percentile of healthy control patients, thereby representing IH; these figures did not vary significantly based on age.[22]

Reported OCT imaging success rates were high. Driessen *et al*[34] reported overall OCT success in 85% of eyes. Dagi *et al*[40] did not specify success rate, but reported that 16.9% were excluded due to limited cooperation, severe nystagmus, poor scan quality or retinal degeneration. Swanson *et al* implied 100% success rate in both studies[22, 23], but acquired OCT images under general anaesthesia and therefore were non-reliant on patient cooperation. Other studies of craniosynostosis did not report OCT success rates.

For peer review only - http://bmjopen.bmj.com/site/about/guidelines.xhtml

Apart from OCT, other surrogate measures of ICP displayed poor sensitivity, limiting their potential as screening tools for IH when used in isolation. These included fundoscopy[22, 23, 33, 40], visual acuity[40], visual fields[40], radiological signs.[22, 23, 40] and clinical history including complaints of headache.[22, 23]

With respect to defining IH, there is no universally agreed clinical consensus on timing, frequency and duration for accurate ICP measurement, or indeed what figure constitutes raised ICP.[50] In the three studies using ICP measurements[22, 33, 49], ICP <10 mmHg was determined as normal, while 10-15 mmHg was determined as borderline and >15 mmHg was determined as raised.

Evidence summary: idiopathic intracranial hypertension

IIH, or primary IH, is characterised by raised ICP in the context of normal CSF composition and no evidence of SOL or ventriculomegaly on neuroimaging.[14] Principles from the modified Dandy criteria[51] and the revised criteria by Friedman *et al*[52] can assist in making diagnosis without ambiguity. Eight studies in this review utilised OCT in patients with IIH.[35, 36, 42, 43, 45-48] These studies included a total of 237 participants, of which 102 were diagnosed with IIH[35, 36, 42, 43, 45-48]. Of the other included participants in these studies, 74 had pseudopapilledema,[43, 46, 48] 3 had SOL[43], 6 had other pathology[43] covered below and 52 were normal controls.[35, 46, 48] OCT devices used include the Cirrus HD-OCT[14] (Carl Zeiss Meditec, Dublin, CA, US), Spectralis[42, 48] and Stratus[35, 47] (Carl Zeiss Meditec, Dublin, CA, US). Three studies did not specify which OCT device was used.[43, 45, 46]

OCT parameters included RNFL thickness[35, 36, 42, 47, 48], macular volume[35], photoreceptor loss[42] and Bruch's membrane opening.[48] Two studies did not specify which OCT parameters were used.[45, 46] OCT demonstrated good diagnostic capability in IIH (Table 2). Increased RNFL thickness[35, 36, 43, 48] and macular volume[35] were associated with IH, while RNFL thinning and photoreceptor loss were associated with optic atrophy and vision loss.[42] In addition, Thompson *et al*[48] found that the transverse diameter of Bruch's membrane opening was enlarged in mild papilloedema and could be used together with RNFL thickness to distinguish mild papilloedema from psuedopapilloedema (Figure 3).

Lee *et al*[36] reported 86.7% OCT imaging success rate, while Sanches-Tocino *et al*[47] reported 100% OCT success. Other studies of IIH did not report success rates.

With regards to other surrogate measures of ICP, Ozturk et al[46] found that optic nerve sheath diameter associated moderately with CSF opening pressure (r=0.661; p<0.005). Lee et al[36] found that body mass index (BMI) associated moderately with lumbar puncture opening pressure (r=0.607; p=0.028). Headache characteristics did not reliably detect children with IIH.[35, 36, 45-47]

With respect to ICP measurements, only Krishnakumar *et al*[45] provided definitions for normal and raised ICP, using lumbar CSF opening and steady state pressures: <15 mmHg = normal CSF pressure; >20 mmHg = high pressure.

Evidence summary: space occupying lesion

Intracranial SOL include tumours or abscesses within the cranial cavity, which are associated with IH. Five studies in this review utilized OCT in patients with SOL.[37, 39, 41, 43, 44] These studies included a total of 121 participants, of which 42 had SOL[37, 39, 41, 43, 44]. Of the remaining participants, 2 had IIH[43], 9 had psuedopapilloedema[43], 10 had other pathology[37, 41, 43] covered below and 58 had optic disc drusen

For peer review only - http://bmjopen.bmj.com/site/about/guidelines.xhtml

with no intracranial pathology.[41] The following OCT devices were used: Cirrus HD-OCT[39, 44], DRI-OCT-1 Atlantis[37], Spectralis[41] and Stratus[39]. Malem *et al*[43] did not specify which device was used.

OCT parameters included RNFL thickness[39, 41, 43, 44], ganglion cell layer thickness[44], anterior bowing of Bruch's membrane[41], neural canal diameter[37], papillary vertical height[37] and anterior lamina cribrosa depth.[37] OCT demonstrated value as part of the clinical work-up of these children (Table 2). RNFL thinning was associated with optic atrophy[39] and visual field loss[39, 44], as was ganglion cell loss.[44] Lee *et al*[37] highlighted the reversibility of papilloedema in patients with SOL following ICP reducing surgery, whereby mean neural canal diameter and papillary vertical height decreased whilst mean anterior lamina cribrosa depth increased (Figure 4). Dahlmann-Noor *et al*[41] reported one patient with intraventricular tumour with increased temporal RNFL thickness, but no bowing of Bruch's membrane.

Dahlmann-Noor *et al*[41] reported OCT imaging success rate of 100%, while other SOL studies did not report success rate.

Clinical history including headache characteristics did not reliably detect children with SOL.[39, 41] Humphrey visual field testing is not designed for children and was difficult or not feasible in many cases.[39, 44]

Lee *et al*[37] reported mean preoperative and postoperative ICP values of 24.0±5.0 and 13.2±6.3 mmHg, but no SOL study defined a normal range for ICP.[39, 41]

Evidence summary: other pathology

Seven studies[22, 31, 32, 37, 38, 41, 43] included children with other pathology associated with IH, including hydrocephalus[22, 37, 38] (n=16), papilloedema of unspecified aetiology[32] (n=5), acute lymphoblastic leukaemia[41] (n=1), aseptic meningitis[43] (n=1), growth hormone replacement therapy[31] (n=1), head injury[43] (n=1), recurrent nephrotic syndrome[31] (n=1), rickets[41] (n=1), sagittal sinus thrombosis[43] (n=3) and sigmoid sinus thrombosis[43] (n=1). Of note, Tran-Viet *et al*[38] used the Envisu C2300 (Bioptigen, Inc, Research Triangle Park, NC) – a handheld OCT device, with which they successfully scanned 25/26 (96%) undilated eyes of conscious infants without sedation. Other OCT devices used were the Spectralis and DRI-OCT-1 Atlantis; Malem *et al*[43] did not specify which device was used.

OCT parameters used in these studies include RNFL thickness[31, 32, 41, 43], volumetric spectral domain (SD) OCT of optic nerve head[32], volumetric enhanced depth imaging (EDI) OCT of optic nerve head [32], neural canal diameter[37], papillary vertical height[37], anterior lamina cribrosa depth[37], and anterior bowing of Bruch's membrane[41] (Table 2). Tran-Viet *et al*[38] did not specify which OCT parameters were used. Chang *et al*[32] found a positive association between optic nerve head volume on SD-OCT and EDI-OCT with papilloedema, but did not specify aetiology. Other associations between OCT parameters and IH have been described above.

OCT imaging success rates were high in these studies: Tran-Viet *et al*[38] reported 96% success, Chang *et al*[32] reported 98% and Dahlmann-Noor *et al*[41] reported 100%. Mrugacz *et al*[31] and Swanson *et al*[22, 23] implied 100% success rates. Other studies did not report OCT success rates.

These conditions were omitted from the recommendations of this review due to low numbers, albeit OCT still demonstrated good potential for detecting IH in these studies.

For peer review only - http://bmjopen.bmj.com/site/about/guidelines.xhtml

Recommendations of this review

This review recommends that OCT can be used to assist diagnosis and management of IH in children with craniosynostosis, IIH and SOL, in conjunction with established clinical measures of ICP. This review could not recommend the widespread use of OCT in all children at risk of IH as standard clinical practice, as no level 1 studies were identified for these conditions. However, this review recognises the value of OCT in paediatric IH, particularly in cases where ICP status is uncertain or borderline, for a number of reasons: i) OCT enables rapid, non-invasive, quantitative cross-sectional measurements of the optic nerve head and retina, not offered by conventional fundoscopy; ii) OCT permits serial measurements to help appreciate evolution in optic nerve head and retinal changes over time, where applicable; iii) OCT could guide and support the overall clinical work-up of affected children.

For peer review only

For peer review only - http://bmjopen.bmj.com/site/about/guidelines.xhtml

Discussion

This systematic review collated a body of evidence evaluating the role of OCT to detect IH, specifically the structural changes associated with papilloedema and optic atrophy. Our review recommended that OCT can be used to assist diagnosis and management of IH in children with craniosynostosis, IIH and SOL, in conjunction with established clinical measures of ICP.

Quality of Evidence

Two independent graders (SRR and MH) identified varying levels of evidence among the 21 included studies, as per the Oxford CEBM.[28] There were no systematic reviews, RCTs or validating cohort studies measuring OCT parameters against established reference standards, hence no study was graded as level 1 evidence. The NIH Quality Assessment Tool for individual studies identified 10 studies of fair quality and 11 studies of poor quality (Table 1). The major limiting factor to quality was use of other surrogate clinical measures as the gold standard for IH, as opposed to direct intraparenchymal ICP monitoring.

Research in context

There is a paucity of clinical guidelines for the management of pediatric IH in general. Searches for relevant clinical guidelines were performed via PubMed using the search terms “guidelines” AND “optical coherence tomography” AND “idiopathic intracranial hypertension” OR “craniosynostosis” OR “space occupying lesion”, which returned two relevant guidelines. Mollan *et al*[53] recently published the first consensus guidelines on management of IIH, which state: “Where visual function is found to be threatened, regular ophthalmic examination must occur because this will influence timely management… Formal documentation of the optic nerve head appearance, such as serial photographs or OCT imaging, is useful.” This is consistent with our review’s recommendation that OCT can be used in children with IIH to support diagnosis and management. The guidelines by Mollan *et al*[53] apply to all patients with IIH and are not specific to children. The Working Group on Craniosynostosis[54] published clinical guidelines for craniosynostosis in 2015, but these did not feature OCT. No relevant guidelines were identified for the use of OCT in SOL. The same search strategy was used for nice.org.uk - the UK’s National Institute for Health and Care Excellence, which returned no relevant guidelines.

The NORDIC Idiopathic Intracranial Hypertension Treatment Trial (IIHTT) is a landmark RCT with a dedicated OCT sub-study committee.[55] Although the resulting papers appeared in our systematic search, they were excluded from our review because they excluded children aged under 18. On further reading, a positive association between ICP and RNFL thickness, total retinal thickness and optic nerve head volume was also found, consistent with our review findings.[55]

This review identified studies using a range of OCT devices. Conventional, table-mounted OCT devices such as the Spectralis may be suitable in school-age children, but are not designed for young infants. Whilst high OCT imaging success rates were reported, many studies were limited to school aged children rather than young infants. By contrast, this review found that the portable iVue device was successfully used for on-table OCTs in young infants under general anaesthesia,[22, 23] while the Envisu C2300 handheld OCT was used in conscious newborns without the need for general anaesthesia or pupil dilation in one feasibility study.[38]

For peer review only - http://bmjopen.bmj.com/site/about/guidelines.xhtml

Of note, handheld OCT has been recently used to describe the normal development of the optic nerve[27] and fovea[15, 16] in infants and children, with excellent feasibility. Handheld OCT has also been utilised in a wide range of other pedatric conditions including retinopathy of prematurity,[56] nystagmus[57], albinism[58], achromatopsia[59], foveal hypoplasia,[60] optic nerve hypoplasia,[61] primary congenital glaucoma[62], microcephaly[63] and others. Handheld OCT may be better tolerated in young children, particularly those with craniosynostosis associated with cognitive delay. However, further research is required to validate this. Therefore, our group has recently commenced prospective research using handheld OCT in craniosynostosis.

Strengths and limitations

This systematic review has a number of strengths. To the best of our knowledge, this is the first systematic review assessing the role of OCT in pediatric IH. Indeed, no such other systematic review appeared in our systematic search. PRISMA Guidelines[24] and rigorous Cochrane methods were followed.[25] Our protocol was registered on PROSPERO[26] and published[27] prior to this study, to promote transparency and avoid duplication. Two independent screeners (SRR and RJM) conducted the systematic search and two independent graders (SRR and MH) completed the quality assessment. A broad search strategy was developed with support from an experienced research librarian. Notably, our search terms did not restrict to certain conditions or age-related keywords – rather, the records were manually searched to avoid missing any key evidence. There were no time or language restrictions, yielding a broad range of eligible studies for inclusion including one article translated from Polish.[31]

We also acknowledge the limitations of this review. Meta-analysis was not possible due to the absence of eligible RCTs and inconsistency in OCT measures and gold standard measures of IH per study. The maximum grade of recommendations from this review was grade B, based on consistent level 2 or 3 studies or extrapolations from level 1 studies.[28] Notably, while some studies demonstrated OCT detection of papilloedema (e.g. RNFL thickening), other studies demonstrated OCT detection of optic atrophy (e.g. RNFL thinning), therefore caution must be exercised in interpreting OCT changes within the full clinical context and in serial examinations as far as possible. No level 1 evidence was returned by this review, which would be required to identify optimal OCT parameters and develop formal clinical guidelines. The broad search strategy resulted in a large number of records to screen, albeit this reduced the risk of missing key evidence.

Further research

This review highlighted the lack of standardisation in OCT parameters used to detect IH in children. Further research is required to clarify the most appropriate OCT parameters for this purpose, using gold standard ICP measures. This could be achieved by a validating prospective study using existing OCT reference standards. This should qualify as level 1b evidence as per the Oxford CEBM.[28] Handheld OCT could enable serial imaging in young infants without sedation, which would be particularly valuable as many pathologies associated with pediatric ICP can begin from birth, however current evidence is lacking. Serial OCT imaging could enable appreciation of evolving optic nerve head and retinal changes over time, where applicable. Further high quality prospective studies could help to integrate OCT into formal clinical guidelines and clinical decision-making algorithms.

For peer review only - http://bmjopen.bmj.com/site/about/guidelines.xhtml

Conclusions

This systematic review has recognised the diagnostic capability of OCT in paediatric IH and recommends that it can be used, in conjunction with established clinical measures of ICP, in craniosynostosis, IIH and SOL, to guide diagnosis and management. However, this review could not recommend the development of formal guidelines, nor the widespread use of OCT in all children at risk of IH as standard clinical practice at this stage. Further validating prospective studies are required to improve our understanding of the clinical utility of OCT in this role (including handheld devices), to establish optimal OCT parameters for paediatric IH and to inform formal clinical guidelines.

For peer review only

For peer review only - http://bmjopen.bmj.com/site/about/guidelines.xhtml

Acknowledgements

We thank the authors of the included studies for providing further data where required for our systematic review. We thank Selina Lock, Research Services Consultant at the University of Leicester David Wilson Library, for providing expert guidance in our systematic search strategy. We thank Krystyna Ulanicka for providing the English translation of the included Polish study. SRR is funded by a National Institute for Health Research (NIHR) Doctoral Fellowship for this research project. This publication presents independent research funded by the MRC and National Institute for Health Research (NIHR). The views expressed are those of the author(s) and not necessarily those of the MRC, the NHS, the NIHR or the Department of Health and Social Care.

Authorship and contribution

Sohaib R. Rufai: Design and conceptualisation of the study; major role in the acquisition of data; analysis and interpretation of the data; drafted the manuscript for intellectual content.

Michael Hisaund: Analysis and interpretation of the data; revised the manuscript for intellectual content.

Noor ul Owase Jeelani: Design and conceptualisation of the study; revised the manuscript for intellectual content; supervision.

Rebecca J. McLean: Guarantor; design and conceptualisation of the study; major role in the acquisition of data; analysis and interpretation of the data; revised the manuscript for intellectual content; supervision.

Data sharing statement

No additional data available.

Ethical approval

No ethical approval was required for conducting this systematic review.

For peer review only

References

1. Quincke, H., Ueber Meningitis serosa und verwandte Zustände. *Deutsche Zeitschrift für Nervenheilkunde*. 1896. 9(3-4): p. 149-168.
2. Matthews YY, Dean F, Lim MJ, et al. Pseudotumor cerebri syndrome in childhood: incidence, clinical profile and risk factors in a national prospective population-based cohort study. *Arch Dis Child*. 2017;102(8):715-721.
3. Gillson N, Jones C, Reem RE, Rogers DL, Zumberge N, Aylward SC. Incidence and Demographics of Pediatric Intracranial Hypertension. *Pediatr Neurol*. 2017;73:42-47.
4. Derderian C, Seaward J. Syndromic craniosynostosis. *Semin Plast Surg*. 2012;26(2):64-75.
5. Jordan CO, Aylward SC. Intracranial hypertension: a current review. *Curr Opin Pediatr*. 2018;30(6):764-774.
6. Zhang X, Medow JE, Iskandar BJ, et al. Invasive and noninvasive means of measuring intracranial pressure: a review. *Physiol Meas*. 2017;38(8):R143-R182. Published 2017 Jul 24.
7. Tamburrini G, Caldarelli M, Massimi L, Santini P, Di Rocco C. Intracranial pressure monitoring in children with single suture and complex craniosynostosis: a review. *Childs Nerv Syst*. 2005;21(10):913-921.
8. Tuite GF, Chong WK, Evanson J, et al. The effectiveness of papilledema as an indicator of raised intracranial pressure in children with craniosynostosis. *Neurosurgery*. 1996;38(2):272-278.
9. Dubourg J, Javouhey E, Geeraerts T, Messerer M, Kassai B. Ultrasonography of optic nerve sheath diameter for detection of raised intracranial pressure: a systematic review and meta-analysis. *Intensive Care Med*. 2011;37(7):1059-1068.
10. Driessen C, Bannink N, Lequin M, et al. Are ultrasonography measurements of optic nerve sheath diameter an alternative to funduscopy in children with syndromic craniosynostosis?. *J Neurosurg Pediatr*. 2011;8(3):329-334.
11. Tuite GF, Evanson J, Chong WK, et al. The beaten copper cranium: a correlation between intracranial pressure, cranial radiographs, and computed tomographic scans in children with craniosynostosis. *Neurosurgery*. 1996;39(4):691-699.
12. Thompson DA, Liasis A, Hardy S, et al. Prevalence of abnormal pattern reversal visual evoked potentials in craniosynostosis. *Plast Reconstr Surg*. 2006;118(1):184-192.
13. Andersson L, Sjölund J, Nilsson J. Flash visual evoked potentials are unreliable as markers of ICP due to high variability in normal subjects. *Acta Neurochir (Wien)*. 2012;154(1):121-127.
14. Lee H, Proudlock FA, Gottlob I. Pediatric Optical Coherence Tomography in Clinical Practice-Recent Progress. *Invest Ophthalmol Vis Sci*. 2016;57(9):OCT69-OCT79.
15. Patel A, Purohit R, Lee H, et al. Optic Nerve Head Development in Healthy Infants and Children Using Handheld Spectral-Domain Optical Coherence Tomography. *Ophthalmology*. 2016;123(10):2147-2157.
16. Lee H, Purohit R, Patel A, et al. In Vivo Foveal Development Using Optical Coherence Tomography. *Invest Ophthalmol Vis Sci*. 2015;56(8):4537-4545.
17. Optical Coherence Tomography Substudy Committee; NORDIC Idiopathic Intracranial Hypertension Study Group. Papilledema Outcomes from the Optical Coherence Tomography Substudy of the Idiopathic Intracranial Hypertension Treatment Trial. *Ophthalmology*. 2015;122(9):1939-45.e2.

For peer review only - http://bmjopen.bmj.com/site/about/guidelines.xhtml

18. Scott CJ, Kardon RH, Lee AG, Frisén L, Wall M. Diagnosis and grading of papilledema in patients with raised intracranial pressure using optical coherence tomography vs clinical expert assessment using a clinical staging scale. *Arch Ophthalmol*. 2010;128(6):705-711.
19. Skau M, Yri H, Sander B, Gerds TA, Milea D, Jensen R. Diagnostic value of optical coherence tomography for intracranial pressure in idiopathic intracranial hypertension. *Graefes Arch Clin Exp Ophthalmol*. 2013;251(2):567-574.
20. Anand A, Pass A, Urfy MZ, et al. Optical coherence tomography of the optic nerve head detects acute changes in intracranial pressure. *J Clin Neurosci*. 2016;29:73-76.
21. Patel MD, Malhotra K, Shirazi Z, Moss HE. Methods for Quantifying Optic Disc Volume and Peripapillary Deflection Volume Using Radial Optical Coherence Tomography Scans and Association With Intracranial Pressure. *Front Neurol*. 2019;10:798.
22. Swanson JW, Aleman TS, Xu W, et al. Evaluation of Optical Coherence Tomography to Detect Elevated Intracranial Pressure in Children. *JAMA Ophthalmol*. 2017;135(4):320-328.
23. Swanson JW, Xu W, Ying GS, et al. Intracranial pressure patterns in children with craniosynostosis utilizing optical coherence tomography. *Childs Nerv Syst*. 2020;36(3):535-544.
24. Moher D, Liberati A, Tetzlaff J, Altman DG; PRISMA Group. Preferred reporting items for systematic reviews and meta-analyses: the PRISMA statement. *Ann Intern Med*. 2009;151(4):264-W64.
25. Cochrane Training. *Cochrane Handbook for Systematic Reviews of* Interventions. 2019. https://training.cochrane.org/handbook [Accessed 10 August 2020]
26. Sohaib Rufai. Detection of intracranial hypertension in children using optical coherence tomography: a systematic review. PROSPERO 2019 CRD42019154254 Available from: https://www.crd.york.ac.uk/prospero/display_record.php?ID=CRD42019154254 [Accessed 10 August 2020]
27. Rufai SR, Jeelani NUO, McLean RJ. Detection of intracranial hypertension in children using optical coherence tomography: a systematic review protocol. BMJ Open. 2020;10(7):e037833. Published 2020 Jul 6.
28. Oxford Centre for Evidence-based Medicine. *Levels of Evidence*. March 2009. https://www.cebm.net/2009/06/oxford-centre-evidence-based-medicine-levels-evidence-march-2009/ [Accessed 10 August 2020]
29. Cochrane. *Data extraction forms.* 2019. https://dplp.cochrane.org/data-extraction-forms [Accessed 10 August 2020]
30. NIH. *Study Quality Assessment Tools: Quality Assessment Tool for Observational Cohort and Cross-Sectional Studies*. https://www.nhlbi.nih.gov/health-topics/study-quality-assessment-tools [Accessed 10 August 2020]
31. Mrugacz M, Szumiński M, Bakunowicz-Łazarczyk A. Optyczna koherentna tomografia w diagnostyce nadciśnienia wewnatrzczaszkowego u dzieci i młodziezy [Optical coherence tomography in diagnosis of intracranial hypertension in children]. *Klin Oczna*. 2011;113(7-9):258-262.
32. Chang MY, Velez FG, Demer JL, et al. Accuracy of Diagnostic Imaging Modalities for Classifying Pediatric Eyes as Papilledema Versus Pseudopapilledema. *Ophthalmology*. 2017;124(12):1839-1848.

For peer review only - http://bmjopen.bmj.com/site/about/guidelines.xhtml

33. den Ottelander BK, de Goederen R, van Veelen MC, et al. Muenke syndrome: long-term outcome of a syndrome-specific treatment protocol [published online ahead of print, 2019 Jul 19]. *J Neurosurg Pediatr*. 2019;1-8.
34. Driessen C, Eveleens J, Bleyen I, van Veelen ML, Joosten K, Mathijssen I. Optical coherence tomography: a quantitative tool to screen for papilledema in craniosynostosis. *Childs Nerv Syst*. 2014;30(6):1067-1073.
35. El-Dairi MA, Holgado S, O'Donnell T, Buckley EG, Asrani S, Freedman SF. Optical coherence tomography as a tool for monitoring pediatric pseudotumor cerebri. *J AAPOS*. 2007;11(6):564-570.
36. Lee YA, Tomsak RL, Sadikovic Z, Bahl R, Sivaswamy L. Use of Ocular Coherence Tomography in Children With Idiopathic Intracranial Hypertension-A Single-Center Experience. *Pediatr Neurol*. 2016;58:101-106.e1.
37. Lee WJ, Kim HJ, Park KH, et al. Change in Optic Nerve After Intracranial Pressure Reduction in Children. *Ophthalmology*. 2017;124(11):1713-1715.
38. Tran-Viet D, Wong BM, Mangalesh S, Maldonado R, Cotten CM, Toth CA. HANDHELD SPECTRAL DOMAIN OPTICAL COHERENCE TOMOGRAPHY IMAGING THROUGH THE UNDILATED PUPIL IN INFANTS BORN PRETERM OR WITH HYPOXIC INJURY OR HYDROCEPHALUS. *Retina*. 2018;38(8):1588-1594.
39. Bialer OY, Goldenberg-Cohen N, Toledano H, Snir M, Michowiz S. Retinal NFL thinning on OCT correlates with visual field loss in pediatric craniopharyngioma. *Can J Ophthalmol*. 2013;48(6):494-499.
40. Dagi LR, Tiedemann LM, Heidary G, Robson CD, Hall AM, Zurakowski D. Using spectral-domain optical coherence tomography to detect optic neuropathy in patients with craniosynostosis. *J AAPOS*. 2014;18(6):543-549.
41. Dahlmann-Noor AH, Adams GW, Daniel MC, et al. Detecting optic nerve head swelling on ultrasound and optical coherence tomography in children and young people: an observational study. *Br J Ophthalmol*. 2018;102(3):318-322.
42. Gospe SM 3rd, Bhatti MT, El-Dairi MA. Anatomic and visual function outcomes in paediatric idiopathic intracranial hypertension. *Br J Ophthalmol*. 2016;100(4):505-509.
43. Malem A, De Salvo G, West S. Use of MultiColor imaging in the assessment of suspected papilledema in 20 consecutive children. *J AAPOS*. 2016;20(6):532-536.
44. Mediero S, Noval S, Bravo-Ljubetic L, Contreras I, Carceller F. Visual Outcomes, Visual Fields, and Optical Coherence Tomography in Paediatric Craniopharyngioma. *Neuroophthalmology*. 2015;39(3):132-139.
45. Krishnakumar D, Pickard JD, Czosnyka Z, Allen L, Parker A. Idiopathic intracranial hypertension in childhood: pitfalls in diagnosis. *Dev Med Child Neurol*. 2014;56(8):749-755.
46. Ozturk Z, Atalay T, Arhan E, et al. The efficacy of orbital ultrasonography and magnetic resonance imaging findings with direct measurement of intracranial pressure in distinguishing papilledema from pseudopapilledema. *Childs Nerv Syst*. 2017;33(9):1501-1507.
47. Sánchez-Tocino H, Bringas R, Iglesias D, González-Pérez A, Del Villar-Galán R. Utilidad del tomógrafo de coherencia óptica (OCT) en el seguimiento de la hipertensión intracraneal idiopática en la infancia [Utility of optic coherence tomography (OCT) in the follow-up of idiopathic intracranial hypertension in childhood]. *Arch Soc Esp Oftalmol*. 2006;81(7):383-389.

For peer review only - http://bmjopen.bmj.com/site/about/guidelines.xhtml

48. Thompson AC, Bhatti MT, El-Dairi MA. Bruch's membrane opening on optical coherence tomography in pediatric papilledema and pseudopapilledema. *J AAPOS*. 2018;22(1):38-43.e3.
49. van de Beeten SDC, Cornelissen MJ, van Seeters RM, et al. Papilledema in unicoronal synostosis: a rare finding [published online ahead of print, 2019 May 17]. *J Neurosurg Pediatr*. 2019;1-6.
50. Hayward R, Britto J, Dunaway D, Jeelani O. Connecting raised intracranial pressure and cognitive delay in craniosynostosis: many assumptions, little evidence. *J Neurosurg Pediatr*. 2016;18(2):242-250.
51. Dandy WE. INTRACRANIAL PRESSURE WITHOUT BRAIN TUMOR: DIAGNOSIS AND TREATMENT. *Ann Surg*. 1937;106(4):492-513.
52. Friedman DI, Liu GT, Digre KB. Revised diagnostic criteria for the pseudotumor cerebri syndrome in adults and children. *Neurology*. 2013;81(13):1159-1165.
53. Mollan SP, Davies B, Silver NC, et al. Idiopathic intracranial hypertension: consensus guidelines on management. *J Neurol Neurosurg Psychiatry*. 2018;89(10):1088-1100.
54. Mathijssen IM. Guideline for Care of Patients With the Diagnoses of Craniosynostosis: Working Group on Craniosynostosis. *J Craniofac Surg*. 2015;26(6):1735-1807.
55. Optical Coherence Tomography Substudy Committee; NORDIC Idiopathic Intracranial Hypertension Study Group. Papilledema Outcomes from the Optical Coherence Tomography Substudy of the Idiopathic Intracranial Hypertension Treatment Trial. *Ophthalmology*. 2015;122(9):1939-45.e2.
56. Anwar S, Nath M, Patel A, et al. POTENTIAL UTILITY OF FOVEAL MORPHOLOGY IN PRETERM INFANTS MEASURED USING HAND-HELD OPTICAL COHERENCE TOMOGRAPHY IN RETINOPATHY OF PREMATURITY SCREENING. *Retina*. 2020;40(8):1592-1602.
57. Lee H, Proudlock F, Gottlob I. Is handheld optical coherence tomography reliable in infants and young children with and without nystagmus?. *Invest Ophthalmol Vis Sci*. 2013;54(13):8152-8159.
58. Mohammad S, Gottlob I, Sheth V, et al. Characterization of Abnormal Optic Nerve Head Morphology in Albinism Using Optical Coherence Tomography. *Invest Ophthalmol Vis Sci*. 2015;56(8):4611-4618.
59. Thomas MG, Kumar A, Kohl S, Proudlock FA, Gottlob I. High-resolution in vivo imaging in achromatopsia. *Ophthalmology*. 2011;118(5):882-887.
60. Rufai SR, Thomas MG, Purohit R, et al. Can Structural Grading of Foveal Hypoplasia Predict Future Vision in Infantile Nystagmus?: A Longitudinal Study. *Ophthalmology*. 2020;127(4):492-500.
61. Pilat A, Sibley D, McLean RJ, Proudlock FA, Gottlob I. High-Resolution Imaging of the Optic Nerve and Retina in Optic Nerve Hypoplasia. *Ophthalmology*. 2015;122(7):1330-1339.
62. Pilat AV, Proudlock FA, Shah S, et al. Assessment of the anterior segment of patients with primary congenital glaucoma using handheld optical coherence tomography. *Eye (Lond)*. 2019;33(8):1232-1239.
63. Papageorgiou E, Pilat A, Proudlock F, et al. Retinal and optic nerve changes in microcephaly: An optical coherence tomography study. *Neurology*. 2018;91(6):e571-e585.

For peer review only - http://bmjopen.bmj.com/site/about/guidelines.xhtml

Figure Legends

Figure 1: PRISMA study inclusion flow diagram. Key: *Reasons for exclusion were as follows: adult studies (n=98); mixed studies of adults and children without breakdown (n=27); conference abstracts (n=86); case reports (n=6); correspondence (n=4). Supplemental Table 1 contains the full list of excluded studies with reasons.

Figure 2. Optical Coherence Tomographic (OCT) Findings, Parameter Variation With Intracranial Pressure (ICP), and Utility as a Screening Test. A, Near infrared reflectance (NIR-REF) and 3-dimensional spectral-domain (SD)–OCT. The white arrowheads indicate blurred optic disc margins. B, Fundal images of patients with normal (patient 1; ICP, 6 mm Hg) and elevated (patient 2; ICP, 18 mm Hg and patient 3; ICP, 20 mm Hg) ICP. C, OCT retinal parameters (maximal retinal thickness, anterior projection, and maximal retinal nerve fiber layer [RNFL] thickness) plotted as a function of ICP measured intraoperatively. D, Receiver operating characteristic (ROC) curves for each of the 3 OCT parameters, combined RNFL and maximal retinal thickness parameters, and a model combining all parameters.

Reprinted with permission from: Swanson JW, Aleman TS, Xu W, et al. Evaluation of Optical Coherence Tomography to Detect Elevated Intracranial Pressure in Children. *JAMA Ophthalmol*. 2017;135(4):320-328, Copyright © 2018 American Medical Association

Figure 3. Measuring the transverse horizontal diameter of Bruch's membrane opening (BMO) and the papillary height on SD-OCT when the ONH is swollen from mild papilledema (A) and when the papilledema has resolved (B).

Reprinted with permission from: Thompson AC, Bhatti MT, El-Dairi MA. Bruch's membrane opening on optical coherence tomography in pediatric papilledema and pseudopapilledema. *J AAPOS*. 2018;22(1):38-43, Copyright © 2018 Elsevier.

Figure 4: Swept-source optical coherence tomography (SS-OCT) images (horizontal scan) of optic nerve head structures in the right eye of an 8-year-old boy with pilocytic astrocytoma. Disc photographs showing preoperative papilledema (A) and postoperative resolution of the same (B) are presented with the indicating OCT section (small squares). A row SS-OCT image of preoperative (C) and postoperative (D) states can be observed. Measurements are obtained using the preoperative (E) and postoperative (F) images. After surgical decompression, the neural canal diameter (red line) and papillary vertical height (green line) have decreased and the lamina cribrosa (LC) shows posterior displacement (yellow dotted line; anterior surface of the LC contour). IICP = increased intracranial pressure.

Reprinted with permission from: Lee WJ, Kim HJ, Park KH, et al. Change in Optic Nerve After Intracranial Pressure Reduction in Children. *Ophthalmology*. 2017;124(11):1713-1715, Copyright © 2017 Elsevier.

For peer review only - http://bmjopen.bmj.com/site/about/guidelines.xhtml

For peer review only

For peer review only

For peer review only - http://bmjopen.bmj.com/site/about/guidelines.xhtml

Source	Country	Study design	Conditions	Total number of study participants	Age	Level of Evidence (1-5)*	Quality rating†
Prospective studies							
**Chang 2017[32]**	US	Prospective observational study	Other	19	M: 11; Ra: 5-17	2b	⊕⊕⊖
**Den Ottelander 2019[33]**	The Netherlands	Prospective cohort study	Craniosynostosis	38	M: 0.7; Ra: 0.2-1.5	2b	⊕⊖⊖
**Driessen 2014[34]**	The Netherlands	Prospective cohort study	Craniosynostosis	38	M: 6.2; Ra: 3.0-11.0	2b	⊕⊖⊖
**El-Dairi 2007[35]**	US	Prospective observational study	IIH	48	Mdn: 11; Ra: 4-14‡	2b	⊕⊕⊖
**Lee 2016[36]**	US	Prospective observational study	IIH	13	M: 14.8; SD 3.1‡	2b	⊕⊕⊖
**Lee 2017[37]**	South Korea	Prospective observational study	SOL, other	10	M: 12.7; SD: 2.7	2b	⊕⊕⊖
**Tran-Viet 2018[38]**	US	Prospective feasibility study	Other	11	Ra: 0.7-2	3b	⊕⊖⊖
**Swanson 2017[22]**	US	Prospective observational study	Craniosynostosis, other	79	M: 2.9; SD: 3.8; Ra: 0.3-15.0‡	2b	⊕⊕⊖
**Swanson 2020[23]**	US	Prospective observational study	Craniosynostosis	80	M: 3.4; SD: 4.7; Ra: 0.2-18	2b	⊕⊖⊖
Retrospective studies							
**Bialer 2013[39]**	Israel	Retrospective chart review	SOL	20	M: 6.5; SD: 3.9	2b	⊕⊖⊖
**Dagi 2014[40]**	US	Retrospective chart review	Craniosynostosis	54	Mdn: 9.3; SD: 4.6	2b	⊕⊖⊖
**Dahlman-Noor 2018[41]**	UK	Retrospective cohort study	SOL, other	61	Mdn: 10.9; IQR: 7.9-13	2b	⊕⊕⊖
**Gospe 2016[42]**	US	Retrospective chart review	IIH	31	7.8; SD: 3.4	2b	⊕⊕⊖
**Malem 2016[43]**	UK	Case series	SOL, IIH, other	20	M: 11; Ra: 5-16	4	⊕⊕⊖
**Mediero 2015[44]**	Spain	Case series	SOL	10	Mdn: 5; Ra: 3-16	4	⊕⊖⊖
**Mrugacz 2011[31]**	Poland§	Case series	IIH, other	4	Mdn: 9.5; Ra: 3-12	4	⊕⊖⊖
**Krishnakumar 2014[45]**	UK	Retrospective chart review	IIH	16	Mdn: 12; Ra: 3-15	3b	⊕⊕⊖
**Ozturk 2017[46]**	Turkey	Retrospective observational study	IIH	16	M: 11.4; SD: 4.1; Ra: 3-17	3b	⊕⊖⊖
**Sanchez-Tocino 2006[47]**	Spain	Case series	IIH	3	M: 8.3; SD: 3.8; Ra: 4-11	4	⊕⊖⊖
**Thompson 2018[48]**	US	Retrospective cohort study	IIH	90	M: 12.2; SD: 3.7‡	2b	⊕⊕⊖
**Van de Beeten 2019[49]**	The Netherlands	Retrospective chart review	Craniosynostosis	104	M: 0.9; Ra: 0.5-1.7	3b	⊕⊖⊖

Table 1: Study characteristics and quality ratings of included studies. Key: * Levels of evidence as per the Oxford Centre for Evidence-Based Medicine; † The National Institutes of Health (NIH) Quality Assessment Tool was used: ⊕⊕⊕, good; ⊕⊕⊖, fair; ⊕⊖⊖, poor; ‡ Where separate age ranges were reported for patients and controls, the age range for patients is displayed here; § Article written in Polish. IIH = idiopathic intracranial hypertension; M = mean; Mdn = median; Ra = range; SD = standard deviation

For peer review only - http://bmjopen.bmj.com/site/about/guidelines.xhtml

Source	OCT Parameter(s)	Gold standard	Diagnostic capability of OCT
Prospective studies			
**Chang 2017**[32]	i) RNFL thickness; ii) volumetric SD-OCT of ONH; iii) volumetric EDI-OCT of ONH	Clinical diagnosis	i) 69% accuracy; ii) 71% accuracy; iii) 67% accuracy
**Den Ottelander 2019**[33]	TRT	Fundoscopy and/or 24 hour ICP	No patients had abnormal TRT
**Driessen 2014**[34]	TRT	Fundoscopy	TRT increased in abnormal fundoscopy (TRT p<0.001)
**El-Dairi 2007**[35]	i) RNFL thickness; ii) macular volume	Modified Dandy IIH criteria	i) Thicker RNFL in IIH vs controls (p<0.0001); ii) thicker macular volume in IIH vs controls (p=0.0008)
**Lee 2016**[36]	RNFL thickness	Fundoscopy	RE: r=0.633, p=0.02; LE: r=0.868, P=0.001
**Lee 2017**[37]	i) Neural canal diameter; ii) papillary vertical height; iii) anterior LC depth	Single ICP measures	i) Post-op reduction (p=0.027); ii) post-op reduction (p<0.001); iii) post-op increase (p=0.001)
**Tran-Viet 2018**[38]	Not specified	Clinical diagnosis	Not assessed
**Swanson 2017**[22]	i) Maximal RNFL thickness; ii) maximal retinal thickness iii) maximal anterior retinal projection; iv) Combination of i) and iii)	Single ICP measures	i) Sens: 79%; Spec: 81%; ii) Sens: 63%; Spec: 86%; iii) Sens: 84%; Spec: 67%; iv) Sens: 89%; Spec: 62%
**Swanson 2020**[23]	Combination of RNFL thickness, maximal retinal thickness and maximal anterior retinal projection	OCT	Not assessed
Retrospective studies			
**Bialer 2013**[39]	RNFL thickness	Clinical diagnosis	Significantly lower in optic atrophy vs. controls (p<0.001)
**Dagi 2014**[40]	RNFL thickness	Fundoscopy	Optic atrophy: Sens: 88%; spec: 94%; papilledema: Sens: 60%; spec: 90%; all: Sens: 77%; spec: 83%
**Dahlman-Noor 2018**[41]	i) RNFL thickness; ii) anterior bowing of BM	Clinical diagnosis	i) Present in 2 in 3 children; ii) present in 1 in 3 children.
**Gospe 2016**[42]	i) Optic atrophy: RNFL thickness <80µm; ii) photoreceptor loss	Revised Friedman IIH criteria	i) OR for any vision loss: infinite (p<0.0001); ii) OR for any vision loss: 120 (p<0.0001)
**Malem 2016**[43]	RNFL thickness	Neuroimaging +/- LP	Not assessed
**Mediero 2015**[44]	i) GCL thickness; ii) RNFL thickness	HVF	i) kappa = 1; p < 0.001; ii) kappa = 0.632; p = 0.011
**Mrugacz 2011**[31]	RNFL thickness	Clinical diagnosis	Not assessed
**Krishnakumar 2014**[45]	Not specified	LP	Not assessed
**Ozturk 2017**[46]	Not specified	LP	Not assessed
**Sanchez-Tocino 2006**[47]	RNFL thickness	LP	Not assessed
**Thompson 2018**[48]	i) BMO; ii) RNFL	Revised Friedman IIH criteria	i) AUC=0.81; ii) AUC=0.96. Combined cut-offs: Sens: 91.7%; Spec: 92.2%
**Van de Beeten 2019**[49]	TRT	Clinical diagnosis	Not assessed

Table 2: Main outcome measures. Key: BM = Bruch's membrane; EDI = enhanced depth imaging; GCL = ganglion cell layer; HVF = Humphrey visual fields; LC = lamina cribrosa; LE = left eye; LP = lumbar puncture; OR = odds ratio; RE = right eye; RNFL = retinal nerve fibre layer; SD = spectral domain; TRT = total retinal thickness; op = operation

For peer review only - http://bmjopen.bmj.com/site/about/guidelines.xhtml

This page has been left blank intentionally

For peer review only

For peer review only - http://bmjopen.bmj.com/site/about/guidelines.xhtml

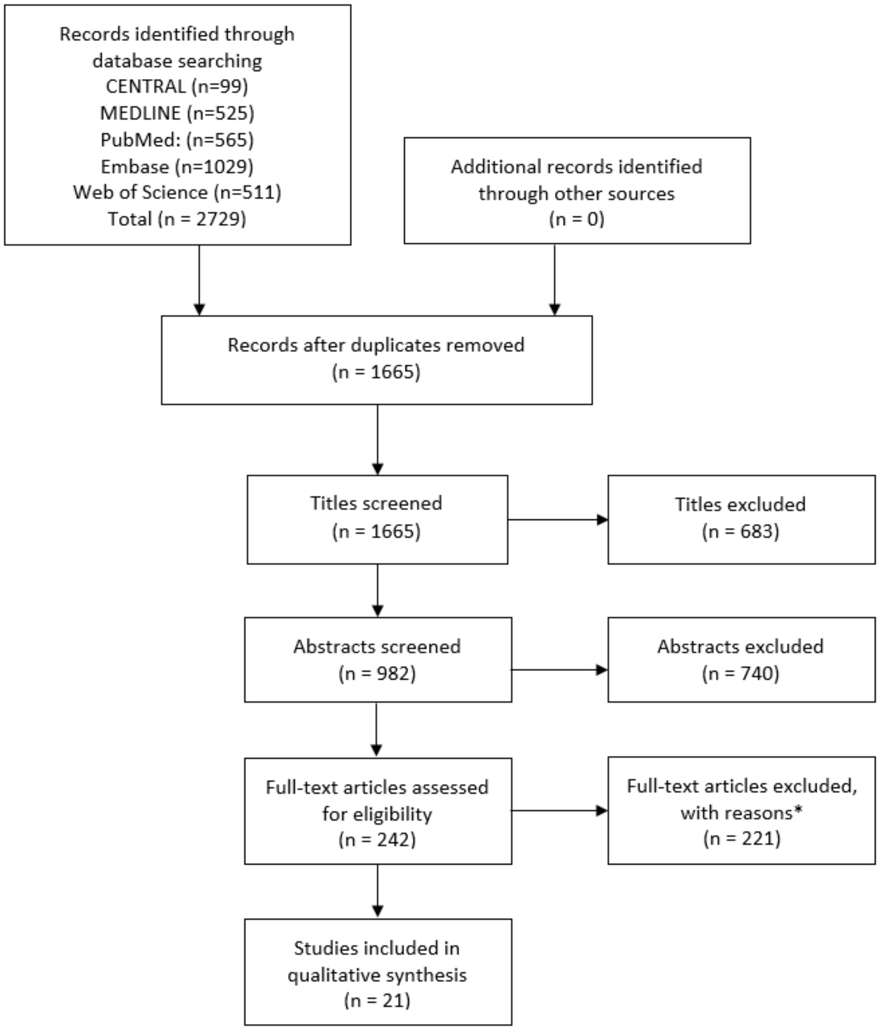

197x218mm (96 x 96 DPI)

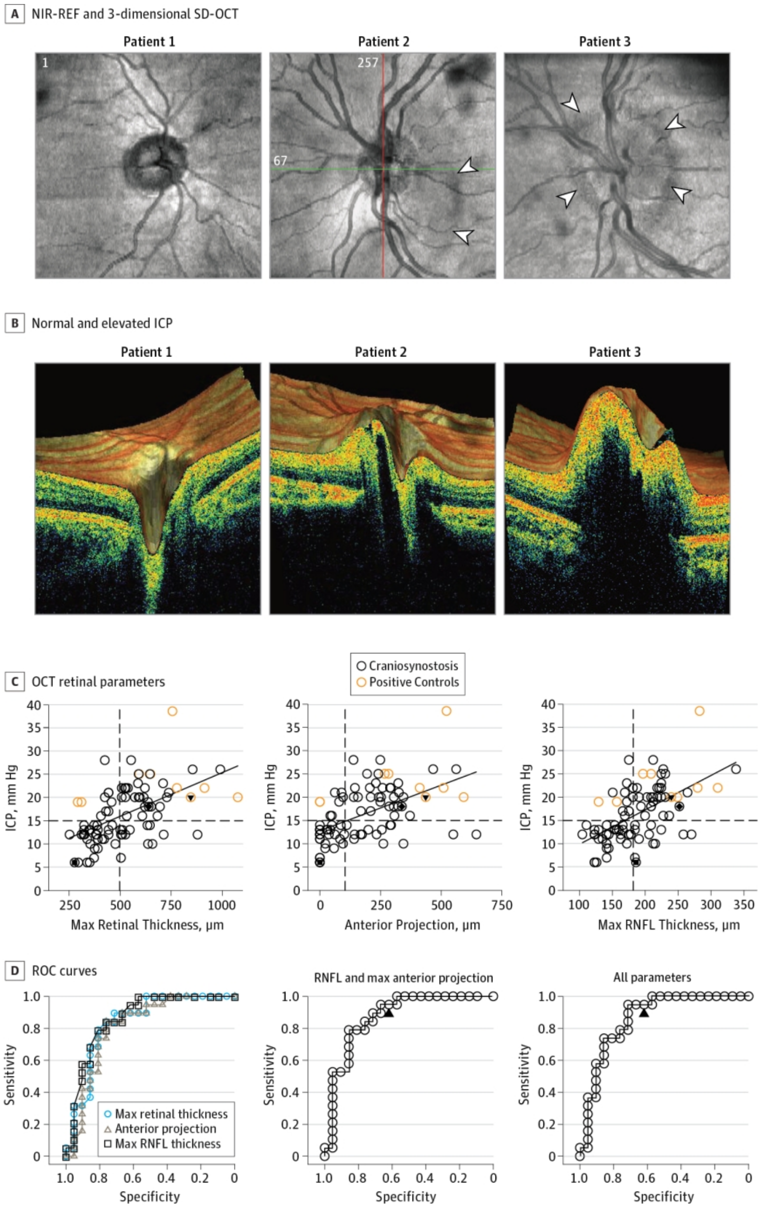
A NIR-REF and 3-dimensional SD-OCT
Patient 1
Patient 2
Patient 3
1
257
67
B Normal and elevated ICP
Patient 1
Patient 2
Patient 3
C OCT retinal parameters
Craniosynostosis
Positive Controls
ICP, mm Hg
Max Retinal Thickness, μm
Anterior Projection, μm
Max RNFL Thickness, μm
D ROC curves
RNFL and max anterior projection
All parameters
Sensitivity
Specificity
Max retinal thickness
Anterior projection
Max RNFL thickness

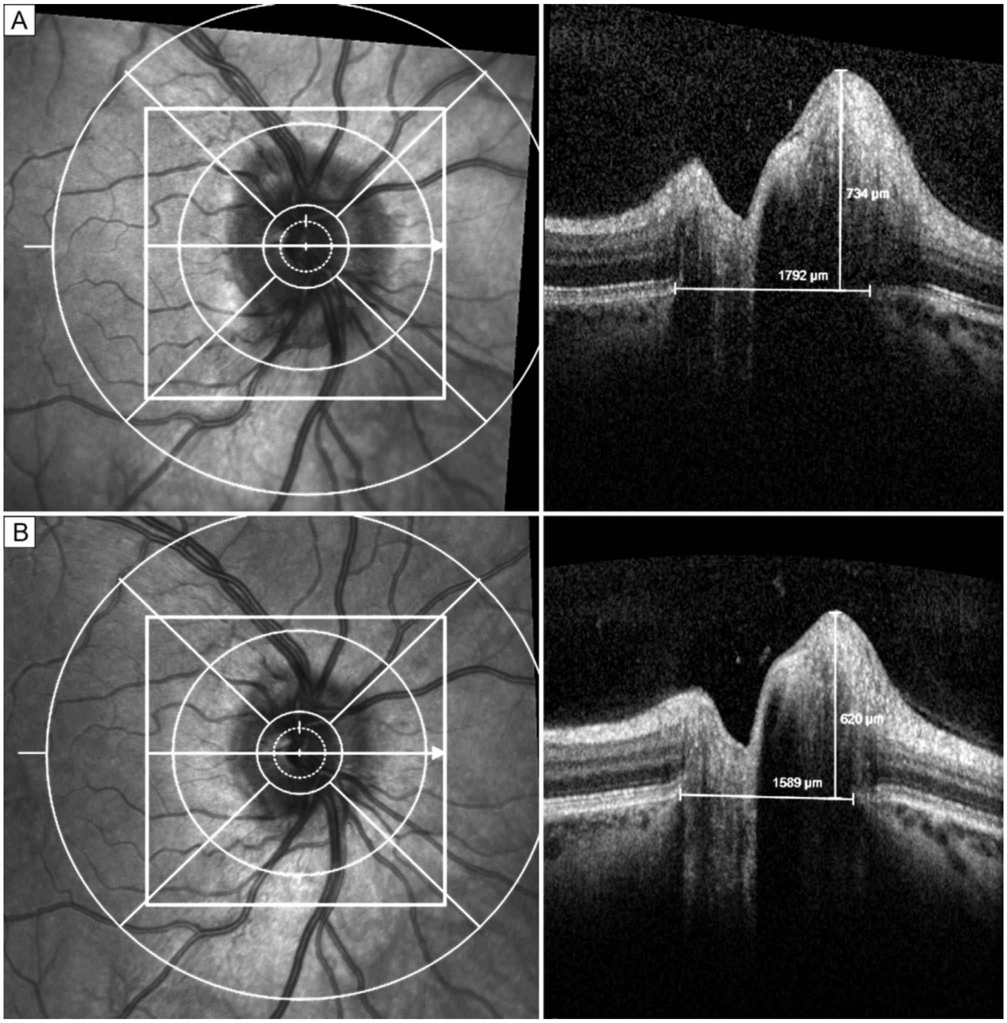

263x267mm (144 x 144 DPI)

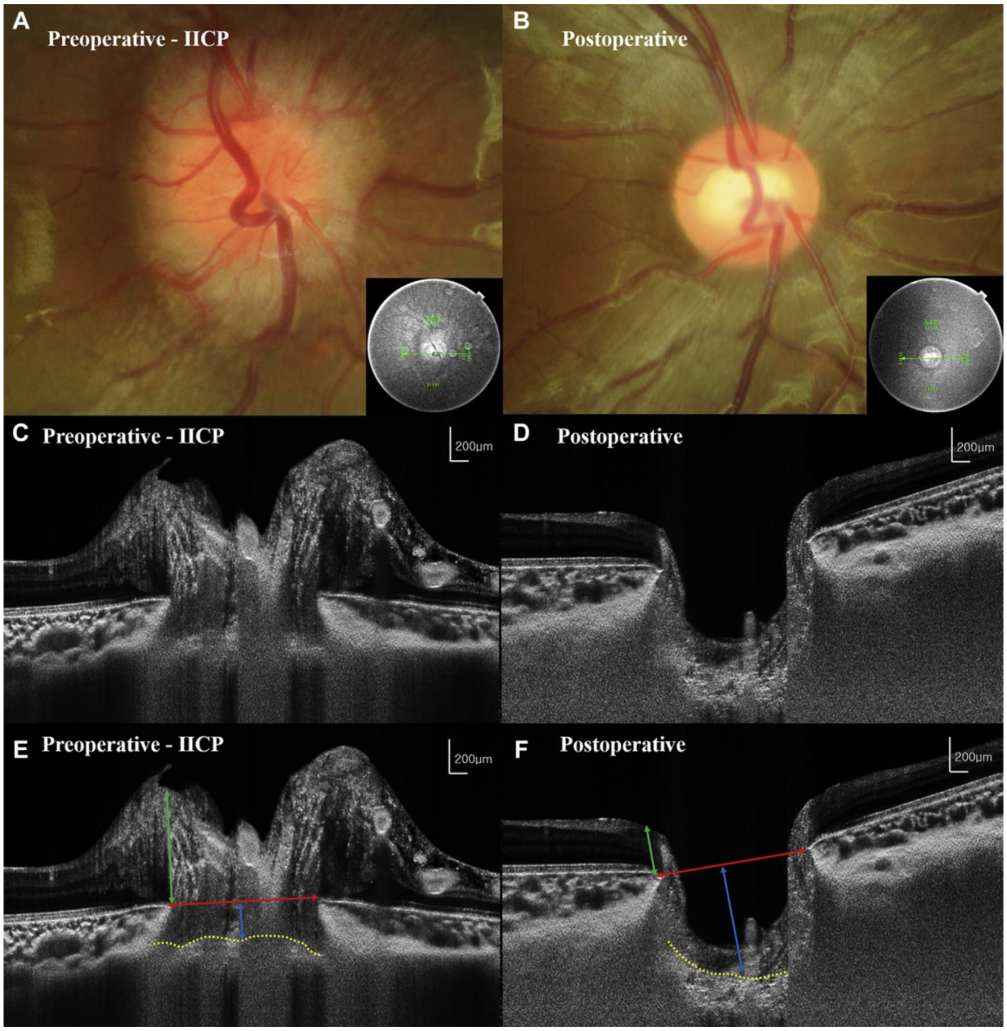

364x372mm (144 x 144 DPI)

Supplemental Data

Contents

Appendix 1: Search terms....................2
Appendix 2: Screening questions....................3
Appendix 3: Data extraction tool, adapted from the Cochrane Collaboration....................4
Appendix 4: NIH Quality Assessment Tool for Observational Cohort and Cross-Sectional Studies ...7
Supplemental Table 1: Excluded studies with reasons....................8

For peer review only

For peer review only - http://bmjopen.bmj.com/site/about/guidelines.xhtml

Appendix 1: Search terms

Intracranial pressure, intracranial hypertension, papilloedema, optical coherence tomography

1. Intracranial pressure
2. ICP
3. Intracerebral pressure
4. Intracranial hypertension
5. Cerebral swelling
6. Cerebral oedema
7. Cerebral edema
8. Brain swelling
9. Brain oedema
10. Brain edema
11. Papilloedema
12. Papilledema
13. Optical coherence tomography
14. OCT

Search combination:

1 OR 2 OR 3 OR 4 OR 5 OR 6 OR 7 OR 8 OR 9 OR 10 OR 11 OR 12

AND

13 or 14

For peer review only

For peer review only - http://bmjopen.bmj.com/site/about/guidelines.xhtml

Appendix 2: Screening questions

Instructions for screeners: Tick the appropriate box per screening question. If "yes" at Stage 1, proceed to Stage 2; if "yes" at Stage 2, include. If "no" at any stage, exclude. If "unclear", proceed to next stage. If still "unclear" after Stage 2, then submit to third arbitrator for verdict.

Stage 1: Title Screening

Does the study represent Level 4 evidence or above, i.e. case series, case-control studies, cohort studies, randomised controlled trials (RCTs) and systematic reviews?

Yes	
No	
Unclear	

Does the study pertain to the use of optical coherence tomography (OCT) to detect raised intracranial pressure (ICP) in children, defined as 0 to 18 years of age?

Yes	
No	
Unclear	

Stage 2: Abstract screening

Does the study represent Level IV evidence or above, i.e. case series, cohort studies, case-control studies, randomised controlled trials (RCTs) and systematic reviews?

Yes	
No	
Unclear	

Does the study pertain to the use of optical coherence tomography (OCT) to detect raised intracranial pressure (ICP) in children, defined as 0 to 18 years of age?

Yes	
No	
Unclear	

Stage 3: Full paper screening

Does the study pertain to the use of optical coherence tomography (OCT) to detect raised intracranial pressure (ICP) in children, defined as 0 to 18 years of age?

Yes	
No	
Unclear	

For peer review only - http://bmjopen.bmj.com/site/about/guidelines.xhtml

For peer review only

Appendix 3: Data extraction tool, adapted from the Cochrane Collaboration

General Information

Study title:
Author(s):
Source:
Date of Extraction:

Methods

Aim of study	
Study design	
Inclusion criteria	
Exclusion criteria	
Method of recruitment	
Method of randomisation (if applicable)	
Blinding (if applicable)	
Study duration	
Ethics approval	

Participants

Population description	
Setting	
Total number randomised	
Mean age	
Male/Female %	

Intervention 1

Group name	
Number in group	
Description	
Intervention parameter 1	
Intervention parameter 2 (if applicable)	
Intervention parameter 3 (if applicable)	
Intervention parameter 4 (if applicable)	
Intervention parameter 5 (if applicable)	
Intervention parameter 6 (if applicable)	
Intervention parameter 7 (if applicable)	
Intervention parameter 8 (if applicable)	
Drop-outs from group	

Outcome 1

Outcome group name	
Time points measured	

For peer review only - http://bmjopen.bmj.com/site/about/guidelines.xhtml

For peer review only

Outcome definition	
Outcome parameter 1	
Outcome parameter 2 (if applicable)	
Outcome parameter 3 (if applicable)	
Outcome parameter 4 (if applicable)	
Outcome parameter 5 (if applicable)	
Outcome parameter 6 (if applicable)	
Outcome parameter 7 (if applicable)	
Outcome parameter 8 (if applicable)	
Person measuring	
Imputation of missing data	

Intervention 2 (if applicable)

Intervention group name	
Number in group	
Description	
Intervention parameter 1	
Intervention parameter 2 (if applicable)	
Intervention parameter 3 (if applicable)	
Intervention parameter 4 (if applicable)	
Intervention parameter 5 (if applicable)	
Intervention parameter 6 (if applicable)	
Intervention parameter 7 (if applicable)	
Intervention parameter 8 (if applicable)	
Drop-outs from group	

Outcome 2 (if applicable)

Outcome group name	
Time points measured	
Outcome definition	
Outcome parameter 1	
Outcome parameter 2 (if applicable)	
Outcome parameter 3 (if applicable)	
Outcome parameter 4 (if applicable)	
Outcome parameter 5 (if applicable)	
Outcome parameter 6 (if applicable)	
Outcome parameter 7 (if applicable)	
Outcome parameter 8 (if applicable)	
Person measuring	
Imputation of missing data	

For peer review only - http://bmjopen.bmj.com/site/about/guidelines.xhtml

Intervention 3 (if applicable)

Intervention group name	
Number in group	
Description	
Intervention parameter 1	
Intervention parameter 2 (if applicable)	
Intervention parameter 3 (if applicable)	
Intervention parameter 4 (if applicable)	
Intervention parameter 5 (if applicable)	
Intervention parameter 6 (if applicable)	
Intervention parameter 7 (if applicable)	
Intervention parameter 8 (if applicable)	
Drop-outs from group	

Outcome 3 (if applicable)

Outcome group name	
Time points measured	
Outcome definition	
Outcome parameter 1	
Outcome parameter 2 (if applicable)	
Outcome parameter 3 (if applicable)	
Outcome parameter 4 (if applicable)	
Outcome parameter 5 (if applicable)	
Outcome parameter 6 (if applicable)	
Outcome parameter 7 (if applicable)	
Outcome parameter 8 (if applicable)	
Person measuring	
Imputation of missing data	

For peer review only

For peer review only - http://bmjopen.bmj.com/site/about/guidelines.xhtml

Appendix 4: NIH Quality Assessment Tool for Observational Cohort and Cross-Sectional Studies

**Criteria**	**Yes**	**No**	**Other (CD, NR, NA)***
1. Was the research question or objective in this paper clearly stated?			
2. Was the study population clearly specified and defined?			
3. Was the participation rate of eligible persons at least 50%?			
4. Were all the subjects selected or recruited from the same or similar populations (including the same time period)? Were inclusion and exclusion criteria for being in the study prespecified and applied uniformly to all participants?			
5. Was a sample size justification, power description, or variance and effect estimates provided?			
6. For the analyses in this paper, were the exposure(s) of interest measured prior to the outcome(s) being measured?			
7. Was the timeframe sufficient so that one could reasonably expect to see an association between exposure and outcome if it existed?			
8. For exposures that can vary in amount or level, did the study examine different levels of the exposure as related to the outcome (e.g., categories of exposure, or exposure measured as continuous variable)?			
9. Were the exposure measures (independent variables) clearly defined, valid, reliable, and implemented consistently across all study participants?			
10. Was the exposure(s) assessed more than once over time?			
11. Were the outcome measures (dependent variables) clearly defined, valid, reliable, and implemented consistently across all study participants?			
12. Were the outcome assessors blinded to the exposure status of participants?			
13. Was loss to follow-up after baseline 20% or less?			
14. Were key potential confounding variables measured and adjusted statistically for their impact on the relationship between exposure(s) and outcome(s)?			

*CD, cannot determine; NA, not applicable; NR, not reported

**Quality Rating (Good, Fair, or Poor)**	
**Rater #1 initials:**	
**Rater #2 initials:**	
**Additional Comments (If POOR, please state why):**	

For peer review only - http://bmjopen.bmj.com/site/about/guidelines.xhtml

Supplemental Table 1: Excluded studies with reasons

Key: *Reasons for exclusion were as follows: A: adult studies (n=98); B: mixed adults and children without breakdown (n=27); C: conference abstracts (n=86); D: case reports (n=6); E: correspondence (n=4).

Number	Reference	Reason for exclusion*
1	Afonso, C.L., et al., Relationship between pattern electroretinogram, frequency-domain OCT, and automated perimetry in chronic papilledema from pseudotumor cerebri syndrome. Investigative Ophthalmology and Visual Science, 2015. 56(6): p. 3656-3665.	A
2	Afonso, J.M., et al., Spectral Domain-Optical Coherence Tomography As a New Diagnostic Marker for Idiopathic Normal Pressure Hydrocephalus. Frontiers in Neurology, 2017. 8: p. 10.	A
3	Aghsaei Fard, M., et al., Optic Nerve Head and Macular Optical Coherence Tomography Measurements in Papilledema Compared With Pseudopapilledema. Journal of neuro-ophthalmology : the official journal of the North American Neuro-Ophthalmology Society, 2019. 39(1): p. 28-34.	A
4	Albrecht, P., et al., Optical coherence tomography for the diagnosis and monitoring of idiopathic intracranial hypertension. Journal of neurology, 2017. 264(7): p. 1370-1380.	A
5	Alessi, G., et al., Evolution en tomographie par coherence optique de l'oedme papillaire dans l'hypertension intracranienne idiopathique traitee par mise en place de stent endosinusien du sinus lateralOptical coherence tomography in following up papilledema in idiopathic intracranial hypertension treated with lateral sinus stent placement. Journal Francais d'Ophtalmologie, 2010. 33(9): p. 637-648.	A
6	Anand, A., et al., Optical coherence tomography of the optic nerve head detects acute changes in intracranial pressure. Journal of Clinical Neuroscience, 2016. 29: p. 73-76.	A
7	Aojula, A., et al., Segmentation error in spectral domain optical coherence tomography measures of the retinal nerve fibre layer thickness in idiopathic intracranial hypertension. BMC ophthalmology, 2018. 17(1): p. 257.	A
8	Asian, F. and B. Ozkal, Optical coherence tomography measurements of the optic nerve head and retina in newly diagnosed idiopathic intracranial hypertension without loss of vision. Ceska a Slovenska Neurologie a Neurochirurgie, 2019. 82(3): p. 309-315.	A
9	Athappilly, G., et al., Ganglion Cell Complex Analysis as a Potential Indicator of Early Neuronal Loss in Idiopathic Intracranial Hypertension. Neuro-ophthalmology (Aeolus Press), 2019. 43(1): p. 10-17.	A
10	Auinger, P., et al., Baseline OCT Measurements in the Idiopathic Intracranial Hypertension Treatment Trial, Part II: Correlations and Relationship to Clinical Features. Investigative Ophthalmology & Visual Science, 2014. 55(12): p. 8173-8179.	A

For peer review only - http://bmjopen.bmj.com/site/about/guidelines.xhtml

11	Auinger, P., et al., Papilledema Outcomes from the Optical Coherence Tomography Substudy of the Idiopathic Intracranial Hypertension Treatment Trial. Ophthalmology, 2015. 122(9): p. 1939-1945.	A
12	Bassi, S.T. and K.P. Mohana, Optical coherence tomography in papilledema and pseudopapilledema with and without optic nerve head drusen. Indian journal of ophthalmology, 2014. 62(12): p. 1146-51.	A
13	Bilen, F.T. and H. Atilla, Peripapillary Vessel Density Measured by Optical Coherence Tomography Angiography in Idiopathic Intracranial Hypertension. Journal of Neuro-Ophthalmology, 2019. 39(3): p. 319-323.	A
14	Carey AR, Bosley TM, Miller NR, McCulley TJ, Henderson AD. Use of En Face Optical Coherence Tomography to Monitor Papilledema in Idiopathic Intracranial Hypertension: A Pilot Study [published online ahead of print, 2020 Mar 24]. J Neuroophthalmol. 2020;10.1097/WNO.0000000000000940. doi:10.1097/WNO.0000000000000940	A
15	Carta, A., et al., Optical coherence tomography is a useful tool in the differentiation between true edema and pseudoedema of the optic disc. PLoS ONE, 2018. 13(11).	A
16	Chen, J.J., et al., Optic disc edema in glial fibrillary acidic protein autoantibody-positive meningoencephalitis. Journal of Neuro-Ophthalmology, 2018. 38(3): p. 276-281.	A
17	Choi, S.S., et al., Changes in cellular structures revealed by ultra-high resolution retinal imaging in optic neuropathies. Investigative ophthalmology & visual science, 2008. 49(5): p. 2103-19.	A
18	Dreesbach, M., et al., Optic nerve head volumetry by optical coherence tomography in papilledema related to idiopathic intracranial hypertension. Translational Vision Science and Technology, 2020. 9(3).	A
19	Eren, Y., et al., Evaluation of optic nerve head changes with optic coherence tomography in patients with idiopathic intracranial hypertension. Acta Neurologica Belgica, 2019. 119(3): p. 351-357.	A
20	Fard, M.A., et al., Optic Nerve Head and Macular Optical Coherence Tomography Measurements in Papilledema Compared With Pseudopapilledema. Journal of Neuro-Ophthalmology, 2019. 39(1): p. 28-34.	A
21	Fard, M.A., et al., Optical Coherence Tomography Angiography in Optic Disc Swelling. American journal of ophthalmology, 2018. 191: p. 116-123.	A
22	Fard, M.A., et al., Optical Coherence Tomography Angiography in Papilledema Compared With Pseudopapilledema. Investigative ophthalmology & visual science, 2019. 60(1): p. 168-175.	A
23	Fard, M.A., et al., Quantification of peripapillary total retinal volume in pseudopapilledema and mild papilledema using spectral-domain optical coherence tomography. American journal of ophthalmology, 2014. 158(1): p. 136-43.	A
24	Flores-Rodríguez, P., P. Gili, and M.D. Martín-Ríos, Sensitivity and specificity of time-domain and spectral-domain optical coherence tomography in differentiating optic nerve head drusen and optic disc oedema. Ophthalmic Physiol Opt, 2012. 32(3): p. 213-21.	A
25	Gampa, A., et al., Quantitative Association Between Peripapillary Bruch's Membrane Shape and Intracranial Pressure. Investigative ophthalmology & visual science, 2017. 58(5): p. 2739-2745.	A

For peer review only - http://bmjopen.bmj.com/site/about/guidelines.xhtml

26	García-Montesinos, J., et al., Relationship between lamina cribrosa displacement and trans-laminar pressure difference in papilledema. Graefes Arch Clin Exp Ophthalmol, 2017. 255(6): p. 1237-1243.	A
27	Geddie, B.E., U.E. Altiparmak, and E.R. Eggenberger, Cup-to-disc ratio in patients with idiopathic intracranial hypertension is smaller than that in normal subjects. Journal of neuro-ophthalmology : the official journal of the North American Neuro-Ophthalmology Society, 2010. 30(3): p. 231-4.	A
28	Gozzi, F., et al., Optical coherence tomography in the differential diagnosis of true edema versus pseudoedema of the optic disc. Acta Ophthalmologica, 2017. 95.	A
29	Group, O.C.T.S.-S.C.f.N.I.I.H.S., et al., Baseline OCT measurements in the idiopathic intracranial hypertension treatment trial, part II: correlations and relationship to clinical features. Investigative ophthalmology & visual science, 2014. 55(12): p. 8173-9.	A
30	Hamill, E., et al., Cup-to-Disc Ratio in Idiopathic Intracranial Hypertension without Papilloedema. Neuro-ophthalmology (Aeolus Press), 2014. 38(2): p. 69-73.	A
31	Hata, M. and K. Miyamoto, Causes and Prognosis of Unilateral and Bilateral Optic Disc Swelling. Neuro-Ophthalmology, 2017. 41(4): p. 187-191.	A
32	Hata, M., et al., Measurement of retinal nerve fiber layer thickness in eyes with optic disc swelling by using scanning laser polarimetry and optical coherence tomography. Clinical ophthalmology (Auckland, N.Z.), 2014. 8: p. 105-11.	A
33	Hoye, V.J., 3rd, et al., Optical coherence tomography demonstrates subretinal macular edema from papilledema. Archives of ophthalmology (Chicago, Ill. : 1960), 2001. 119(9): p. 1287-90.	A
34	Huang-Link, Y., et al., Optical coherence tomography represents a sensitive and reliable tool for routine monitoring of idiopathic intracranial hypertension with and without papilledema. European journal of neurology, 2019. 26(5): p. 808-e57.	A
35	Huang-Link, Y.M., et al., OCT measurements of optic nerve head changes in idiopathic intracranial hypertension. Clinical Neurology and Neurosurgery, 2015. 130: p. 122-127.	A
36	Igarashi, N., et al., Optic disc cupping characteristics of normal pressure hydrocephalus patients with normal-tension glaucoma. Scientific reports, 2019. 9(1): p. 3108.	A
37	Intracranial, N.I., Baseline OCT Measurements in the Idiopathic Intracranial Hypertension Treatment Trial, Part I: Quality Control, Comparisons, and Variability (vol 55, pg 8180, 2014). Investigative Ophthalmology & Visual Science, 2016. 57(15): p. 6909-6909.	A
38	Johnson, L.N., et al., Differentiating optic disc edema from optic nerve head drusen on optical coherence tomography. Archives of ophthalmology (Chicago, Ill. : 1960), 2009. 127(1): p. 45-9.	A
39	Kamil, M.A.S., et al., Association between papilledema and guillian - Barre syndrome. Indian Journal of Public Health Research and Development, 2018. 9(12): p. 549-555.	A
40	Kaufhold, F., et al., Optic nerve head quantification in idiopathic intracranial hypertension by spectral domain OCT. PloS one, 2012. 7(5): p. e36965.	A

For peer review only - http://bmjopen.bmj.com/site/about/guidelines.xhtml

41	Kim, B.H. and E.J. Lee, Optic Disc Swelling After Intraocular Pressure Lowering Treatment in Acute Primary Angle Closure. Journal of glaucoma, 2017. 26(2): p. e87-e89.	A
42	Kim, M.J. and S.P. Kelly, Optical Coherence Tomography Imaging of Paton Folds in Papilledema With Retinopathy From Systemic Hypertension. JAMA ophthalmology, 2016. 134(10): p. e162121.	A
43	Koraysha, N.A., et al., Evaluating optic nerve diameter as a possible biomarker for disability in patients with multiple sclerosis. Neuropsychiatric Disease and Treatment, 2019. 15: p. 2571-2578.	A
44	Kupersmith, M., Baseline optical coherence tomography (OCT) measurements in the idiopathic intracranial hypertension treatment trial: Correlations and relationship to clinical features. Neurology, 2014. 82(10).	A
45	Kupersmith, M., Effects of intervention on optical imaging of papilledema in the idiopathic intracranial hypertension treatment trial. Neurology, 2015. 84.	A
46	Kupersmith, M., et al., Papilledema Outcomes from the Optical Coherence Tomography Substudy of the Idiopathic Intracranial Hypertension Treatment Trial. Ophthalmology, 2015. 122(9): p. 1939-1945.	A
47	Kupersmith, M.J. and N.I.S. Grp, Baseline Optical Coherence Tomography (OCT) of Participants in the Idiopathic Intracranial Hypertension Treatment Trial: Correlations and Relationships to Clinical Features. Investigative Ophthalmology & Visual Science, 2014. 55(13): p. 2.	A
48	Kupersmith, M.J., Baseline OCT measurements in the idiopathic intracranial hypertension treatment trial, part I: Quality control, comparisons, and variability. Investigative Ophthalmology and Visual Science, 2014. 55(12): p. 8180-8188.	A
49	Kupersmith, M.J., et al., Optical coherence tomography of the swollen optic nerve head: Deformation of the peripapillary retinal pigment epithelium layer in papilledema. Investigative Ophthalmology and Visual Science, 2011. 52(9): p. 6558-6564.	A
50	Kupersmith, M.J., et al., Papilledema Outcomes from the Optical Coherence Tomography Substudy of the Idiopathic Intracranial Hypertension Treatment Trial. Ophthalmology, 2015. 122(9): p. 1939-+.	A
51	Kupersmith, M.J., et al., Scanning laser polarimetry reveals status of RNFL integrity in eyes with optic nerve head swelling by OCT. Investigative ophthalmology & visual science, 2012. 53(4): p. 1962-70.	A
52	Kupersmith, M.J., Papilledema outcome evaluation in the OCT substudy of the idiopathic intracranial hypertension treatment trial. Investigative Ophthalmology and Visual Science, 2015. 56(7): p. 2235.	A
53	Labib, D.M. and D.H.A. Raouf, Diagnostic value of optical coherence tomography in patients with idiopathic intracranial hypertension. Egyptian Journal of Neurology Psychiatry and Neurosurgery, 2015. 52(4): p. 249-253.	A
54	Laemmer, R., et al., Detection of nerve fiber atrophy in apparently effectively treated papilledema in idiopathic intracranial hypertension. Graefes Archive for Clinical and Experimental Ophthalmology, 2010. 248(12): p. 1787-1793.	A
55	Lee, K.M., S.J. Woo, and J.-M. Hwang, Differentiation of optic nerve head drusen and optic disc edema with spectral-domain optical coherence tomography. Ophthalmology, 2011. 118(5): p. 971-7.	A

For peer review only - http://bmjopen.bmj.com/site/about/guidelines.xhtml

56	Liu, K.C., et al., Venous sinus stenting for reduction of intracranial pressure in IIH: A prospective pilot study. Journal of Neurosurgery, 2017. 127(5): p. 1126-1133.	A
57	Malhotra, K., et al., Association Between Peripapillary Bruch's Membrane Shape and Intracranial Pressure: Effect of Image Acquisition Pattern and Image Analysis Method, a Preliminary Study. Frontiers in neurology, 2018. 9: p. 1137.	A
58	Margolin, E., Diagnosis and grading of papilledema in patients with raised intracranial pressure using optical coherence tomography versus clinical expert assessment using a clinical staging scale. Evidence-Based Ophthalmology, 2011. 12(1): p. 42-43.	A
59	Marzoli, S.B., et al., Quantitative analysis of optic nerve damage in idiopathic intracranial hypertension (IIH) at diagnosis. Neurological sciences : official journal of the Italian Neurological Society and of the Italian Society of Clinical Neurophysiology, 2013. 34 Suppl 1: p. S143-5.	A
60	Monteiro, M.L. and C.L. Afonso, Macular thickness measurements with frequency domain-OCT for quantification of axonal loss in chronic papilledema from pseudotumor cerebri syndrome. Eye (Lond), 2014. 28(4): p. 390-8.	A
61	Oner, A., A. Agadayi, and N. Sinim, Optik Disk Odemi ve Optik Disk Druzeni Ayriminda Spektral-Domain Optik Kohorens Tomografi KullanilmasiDifferentiating optic disc edema from optic nerve head drusen with spectral domain optical coherence tomography. Retina-Vitreus, 2015. 23(2): p. 141-144.	A
62	Optical Coherence Tomography Substudy Committee; NORDIC Idiopathic Intracranial Hypertension Study Group. Papilledema Outcomes from the Optical Coherence Tomography Substudy of the Idiopathic Intracranial Hypertension Treatment Trial. Ophthalmology, 2015. 122(9): p. 1939-45.e2.	A
63	Optical Coherence Tomography Substudy, C. and N.I.I.H.S. Group, Papilledema Outcomes from the Optical Coherence Tomography Substudy of the Idiopathic Intracranial Hypertension Treatment Trial. Ophthalmology, 2015. 122(9): p. 1939-45.e2.	A
64	Pardon LP, Cheng H, Tang RA, Saenz R, Frishman LJ, Patel NB. Custom Optical Coherence Tomography Parameters for Distinguishing Papilledema from Pseudopapilledema. Optom Vis Sci. 2019;96(8):599-608. doi:10.1097/OPX.0000000000001408	A
65	Pardon, L.P., et al., Custom Optical Coherence Tomography Parameters for Distinguishing Papilledema from Pseudopapilledema. Optometry and vision science : official publication of the American Academy of Optometry, 2019. 96(8): p. 599-608.	A
66	Pasaoglu, I., et al., Lamina cribrosa surface position in idiopathic intracranial hypertension with swept-source optical coherence tomography. Indian journal of ophthalmology, 2019. 67(7): p. 1085-1088.	A
67	Patel, M.D., et al., Methods for Quantifying Optic Disc Volume and Peripapillary Deflection Volume Using Radial Optical Coherence Tomography Scans and Association With Intracranial Pressure. Frontiers in neurology, 2019. 10: p. 798.	A
68	Pilat, A.V., et al., Macular morphology in patients with optic nerve head drusen and optic disc edema. Ophthalmology, 2014. 121(2): p. 552-7.	A

For peer review only - http://bmjopen.bmj.com/site/about/guidelines.xhtml

69	Pilat, A.V., et al., Morphology of retinal vessels in patients with optic nerve head drusen and optic disc edema. Investigative ophthalmology & visual science, 2014. 55(6): p. 3484-90.	A
70	Rebolleda, G. and F.J. Muñoz-Negrete, Follow-up of mild papilledema in idiopathic intracranial hypertension with optical coherence tomography. Invest Ophthalmol Vis Sci, 2009. 50(11): p. 5197-200.	A
71	Rodriguez Torres Y, Lee P, Mihlstin M, Tomsak RL. Correlation Between Optic Disc Peripapillary Capillary Network and Papilledema Grading in Patients With Idiopathic Intracranial Hypertension: A Study of Optical Coherence Tomography Angiography [published online ahead of print, 2020 Jan 16]. J Neuroophthalmol. 2020;10.1097/WNO.0000000000000877. doi:10.1097/WNO.0000000000000877	A
72	Saenz, R., et al., Use of A-scan Ultrasound and Optical Coherence Tomography to Differentiate Papilledema From Pseudopapilledema. Optometry and vision science : official publication of the American Academy of Optometry, 2017. 94(12): p. 1081-1089.	A
73	Sarac, O., et al., Differentiation of optic disc edema from optic nerve head drusen with spectral-domain optical coherence tomography. Journal of neuro-ophthalmology : the official journal of the North American Neuro-Ophthalmology Society, 2012. 32(3): p. 207-11.	A
74	Savini, G., et al., Detection and quantification of retinal nerve fiber layer thickness in optic disc edema using stratus OCT. Archives of Ophthalmology, 2006. 124(8): p. 1111-1117.	A
75	Scott, C.J., et al., Diagnosis and grading of papilledema in patients with raised intracranial pressure using optical coherence tomography vs clinical expert assessment using a clinical staging scale. Archives of Ophthalmology, 2010. 128(6): p. 705-711.	A
76	Sheils, C.R., et al., The Relationship Between Optic Disc Volume, Area, and Frisen Score in Patients With Idiopathic Intracranial Hypertension. American journal of ophthalmology, 2018. 195: p. 101-109.	A
77	Sibony, P., et al., Optical coherence tomography shape analysis of the peripapillary retinal pigment epithelium layer in presumed optic nerve sheath meningiomas. Journal of neuro-ophthalmology : the official journal of the North American Neuro-Ophthalmology Society, 2014. 34(2): p. 130-6.	A
78	Sibony, P., M.J. Kupersmith, and F.J. Rohlf, Shape analysis of the peripapillary RPE layer in papilledema and ischemic optic neuropathy. Investigative ophthalmology & visual science, 2011. 52(11): p. 7987-95.	A
79	Sibony, P.A., et al., Retinal and Choroidal Folds in Papilledema. Investigative ophthalmology & visual science, 2015. 56(10): p. 5670-80.	A
80	Sibony, P.A., Gaze Evoked Deformations of the Peripapillary Retina in Papilledema and Ischemic Optic Neuropathy. Investigative ophthalmology & visual science, 2016. 57(11): p. 4979-4987.	A
81	Sinclair, A.J., et al., Low energy diet and intracranial pressure in women with idiopathic intracranial hypertension: prospective cohort study. BMJ (Clinical research ed.), 2010. 341: p. c2701.	A
82	Skau, M., et al., Disease activity in idiopathic intracranial hypertension: A 3-month follow-up study. Journal of Neurology, 2011. 258(2): p. 277-283.	A

For peer review only - http://bmjopen.bmj.com/site/about/guidelines.xhtml

83	Skau, M., et al., OCT for optic disc evaluation in idiopathic intracranial hypertension. Graefe's Archive for Clinical and Experimental Ophthalmology, 2011. 249(5): p. 723-730.	A
84	Smith, K.A., et al., A case series of dural venous sinus stenting in idiopathic intracranial hypertension: association of outcomes with optical coherence tomography¹. International Journal of Neuroscience, 2017. 127(2): p. 145-153.	A
85	Smith, S.V. and D.I. Friedman, The Idiopathic Intracranial Hypertension Treatment Trial: a Review of the Outcomes. Headache, 2017. (no pagination).	A
86	Starks, V., et al., Effect of optic nerve sheath fenestration for idiopathic intracranial hypertension on retinal nerve fiber layer thickness. Orbit (Amsterdam, Netherlands), 2016. 35(2): p. 87-90.	A
87	Steinegger, K., C. Bergin, and Y. Guex-Crosier, Malignant hypertension: clinical manifestations of 7 cases. Klinische Monatsblatter fur Augenheilkunde, 2015. 232(4): p. 590-2.	A
88	Tatar, I.T., et al., Morphological assessment of lamina cribrosa in idiopathic intracranial hypertension. Indian journal of ophthalmology, 2020. 68(1): p. 164-167.	A
89	Teismann, N., et al., Point-of-care ocular ultrasound to detect optic disc swelling. Academic Emergency Medicine, 2013. 20(9): p. 920-925.	A
90	Tirakotai, W., et al., Endoscopic Optic Nerve Sheath Fenestration for Treatment of Papilledema Secondary to Intracranial Venous Hypertension: Report of Two Cases. Journal of the Medical Association of Thailand = Chotmaihet thangphaet, 2016. 99 Suppl 3: p. S141-6.	A
91	Tsikata, E., et al., Volumetric Measurement of Optic Nerve Head Drusen Using Swept-Source Optical Coherence Tomography. Journal of Glaucoma, 2017. 26(9): p. 798-804.	A
92	Verma, R., et al., Ophthalmological manifestation in patients of tuberculous meningitis. QJM : monthly journal of the Association of Physicians, 2019. 112(6): p. 409-419.	A
93	Waisbourd, M., et al., OCT assessment of morphological changes of the optic nerve head and macula in idiopathic intracranial hypertension. Clinical neurology and neurosurgery, 2011. 113(10): p. 839-43.	A
94	Wall, M., et al., Threshold Static Automated Perimetry of the Full Visual Field in Idiopathic Intracranial Hypertension. Investigative Ophthalmology & Visual Science, 2019. 60(6): p. 1898-1905.	A
95	Wang, J.K., et al., Peripapillary retinal pigment epithelium layer shape changes from acetazolamide treatment in the idiopathic intracranial hypertension treatment trial. Investigative Ophthalmology and Visual Science, 2017. 58(5): p. 2554-2565.	A
96	Wang, J.K., et al., Three-dimensional bruch's membrane shape change over time with acetazolamide treatment in the idiopathic intracranial hypertension treatment trial (IIHTT). Investigative Ophthalmology and Visual Science, 2017. 58(8).	A
97	Wartak, A., et al., Investigating spontaneous retinal venous pulsation using Doppler optical coherence tomography. Scientific Reports, 2019. 9: p. 11.	A
98	Yri, H.M., et al., The course of headache in idiopathic intracranial hypertension: a 12-month prospective follow-up study. Eur J Neurol, 2014. 21(12): p. 1458-64.	A

For peer review only

For peer review only - http://bmjopen.bmj.com/site/about/guidelines.xhtml

99	Bahnasy, W.S., et al., Neuro-ophthalmological biomarkers of visual outcome in newly diagnosed idiopathic intracranial hypertension. Egyptian Journal of Neurology Psychiatry and Neurosurgery, 2019. 55(1): p. 8.	B
100	Casas, P., et al., Retinal nerve fibre layer evaluation using optical coherence tomography in patients with obstructive sleep apnea syndrome (OSAS). Neuro-Ophthalmology, 2011. 35.	B
101	Chang, Y.C., et al., Relationship Between Optic Nerve Protrusion Measured by OCT and MRI and Papilledema Severity. Invest Ophthalmol Vis Sci, 2015. 56(4): p. 2297-302.	B
102	Chen, J.J., et al., Causes and Prognosis of Visual Acuity Loss at the Time of Initial Presentation in Idiopathic Intracranial Hypertension. Investigative Ophthalmology & Visual Science, 2015. 56(6): p. 3850-3859.	B
103	Chen, Q., et al., Pseudotumour Cerebri Syndrome in China: A Cohort Study. Scientific reports, 2020. 10(1): p. 1222.	B
104	Figus, M., et al., Optical Coherence Tomography in Patients with Chiari I Malformation. Biomed Research International, 2015: p. 7.	B
105	Ghasemi Falavarjani K, Tian JJ, Akil H, Garcia GA, Sadda SR, Sadun AA. SWEPT-SOURCE OPTICAL COHERENCE TOMOGRAPHY ANGIOGRAPHY OF THE OPTIC DISK IN OPTIC NEUROPATHY. Retina. 2016;36 Suppl 1:S168-S177. doi:10.1097/IAE.0000000000001259	B
106	Goldhagen BE, Bhatti MT, Srinivasan PP, Chiu SJ, Farsiu S, El-Dairi MA. Retinal atrophy in eyes with resolved papilledema detected by optical coherence tomography. J Neuroophthalmol. 2015;35(2):122-126. doi:10.1097/WNO.0000000000000210	B
107	Goldhagen, B.E., et al., Retinal atrophy in eyes with resolved papilledema detected by optical coherence tomography. Journal of Neuro-Ophthalmology, 2015. 35(2): p. 122-123.	B
108	Ju, D.G., et al., Clinical Significance of Tumor-Related Edema of Optic Tract Affecting Visual Function in Patients with Sellar and Suprasellar Tumors. World Neurosurg, 2019. 132: p. e862-e868.	B
109	Karam, E.Z. and T.R. Hedges, Optical coherence tomography of the retinal nerve fibre layer in mild papilloedema and pseudopapilloedema. The British journal of ophthalmology, 2005. 89(3): p. 294-8.	B
110	Kasl, Z., et al., [The Current Diagnostic Possibilities and Cooperation of Oftalmologist and Neurologist Concerning in Patients with Idiopatic Intracranial Hypertension]. Soucasne moznosti oftalmologicke diagnostiky a spoluprace oftalmologa s neurologem u pacientu s idiopatickou intrakranialni hypertenzi., 2016. 72(2): p. 32-8.	B
111	Kim, M.S., et al., Morphologic Features of Buried Optic Disc Drusen on En Face Optical Coherence Tomography and Optical Coherence Tomography Angiography. American Journal of Ophthalmology, 2020. 213: p. 125-133.	B
112	Koktekir, E., et al., Resolution of papilledema after endoscopic third ventriculostomy versus cerebrospinal fluid shunting in hydrocephalus: A comparative study: Clinical article. Journal of Neurosurgery, 2014. 120(6): p. 1465-1470.	B
113	Kulkarni, K.M., et al., Differentiating mild papilledema and buried optic nerve head drusen using spectral domain optical coherence tomography. Ophthalmology, 2014. 121(4): p. 959-63.	B

For peer review only - http://bmjopen.bmj.com/site/about/guidelines.xhtml

114	Martinez, M.R. and A. Ophir, Optical coherence tomography as an adjunctive tool for diagnosing papilledema in young patients. Journal of pediatric ophthalmology and strabismus, 2011. 48(3): p. 174-81.	B
115	Merticariu, C.I., et al., Optical coherence tomography assessment of structural changes in the optic nerve head and peripapillary retina in idiopathic intracranial hypertension. Archives of the Balkan Medical Union, 2019. 54(2): p. 267-273.	B
116	Rizzo, J.L., et al., Perimetry, retinal nerve fiber layer thickness and papilledema grade after cerebrospinal fluid shunting in patients with idiopathic intracranial hypertension. Journal of neuro-ophthalmology : the official journal of the North American Neuro-Ophthalmology Society, 2015. 35(1): p. 22-5.	B
117	Sajjadi, F., et al., New predictive value of optical coherence tomography analysis in the diagnosis of idiopathic intracranial hypertension. Journal of Contemporary Medical Sciences, 2017. 3(10): p. 197-207.	B
118	Santos, M.A.K., et al., [Optic disc changes by optical coherence tomography in optic disc edema in Lomé]. J Fr Ophtalmol, 2017. 40(4): p. 314-318.	B
119	Sibony, P., et al., Effects of lowering cerebrospinal fluid pressure on the shape of the peripapillary retina in intracranial hypertension. Investigative Ophthalmology and Visual Science, 2014. 55(12): p. 8223-8231.	B
120	Tang, L., et al., Quantitative evaluation of papilledema from stereoscopic color fundus photographs. Investigative Ophthalmology and Visual Science, 2012. 53(8): p. 4490-4497.	B
121	Tang, Y., et al., Assessment of visual function by optical coherence tomography and visual field for craniopharyngioma patients. International Eye Science, 2015. 15(9): p. 1621-1624.	B
122	Tüntaş Bilen, F. and H. Atilla, Peripapillary Vessel Density Measured by Optical Coherence Tomography Angiography in Idiopathic Intracranial Hypertension. J Neuroophthalmol, 2019. 39(3): p. 319-323.	B
123	Vartin C, V., et al., Detection of mild papilloedema using spectral domain optical coherence tomography. The British journal of ophthalmology, 2012. 96(3): p. 375-9.	B
124	Wang, J.K., et al., Automated quantification of volumetric optic disc swelling in papilledema using spectral-domain optical coherence tomography. Investigative Ophthalmology and Visual Science, 2012. 53(7): p. 4069-4075.	B
125	Zhang, L., C. Sun, and X. Sun, [The clinical features and value of macular ganglion cell complex thickness patterns in patients with optic chiasma lesion]. Zhonghua Yan Ke Za Zhi, 2016. 52(5): p. 335-42.	B
126	Afonso, C., et al., Evaluation of inner retinal thicknesses in the macula of patients with chronic papilledema from pseudotumor cerebri syndrome using frequency domain OCT. Investigative Ophthalmology and Visual Science, 2013. 54(15).	C
127	Agne, J., et al., Automatic Detection of Folds and Wrinkles Due to Swelling of the Optic Disc, in Fetal, Infant and Ophthalmic Medical Image Analysis, M.J. Cardoso and T. Arbel, Editors. 2017, Springer International Publishing Ag: Cham. p. 235-242.	C
128	Agne, J., et al., Determining Degree of Optic Nerve Edema from Color Fundus Photography, in Medical Imaging 2015: Computer-Aided	C

For peer review only - http://bmjopen.bmj.com/site/about/guidelines.xhtml

	Diagnosis, L.M. Hadjiiski and G.D. Tourassi, Editors. 2015, Spie-Int Soc Optical Engineering: Bellingham.	
129	Agne, J., et al., Monitoring Intracranial Pressure By a Semi-automated Determination of Bruch's Membrane Deformation From Routine SD-OCT Volume Scans of the Optic Disc. Investigative Ophthalmology & Visual Science, 2016. 57(12): p. 3.	C
130	Chang, L., et al., Optical coherence tomography in the evaluation of neurofibromatosis type 1 subjects with optic pathway gliomas. Journal of AAPOS, 2010. 14(1).	C
131	Chang, L., et al., Optical coherence tomography in the evaluation of neurofibromatosis type 1 subjects with optic pathway gliomas. Journal of AAPOS, 2010. 14(1).	C
132	Chang, M., et al., Accuracy of ultrasonography, fundus photography, autofluorescence (AF), fluorescein angiography (FA), and optical coherence tomography (OCT) in differentiating pseudopapilledema from true optic disk edema (ODE) in children. Journal of AAPOS, 2017. 21(4).	C
133	Christodoulou, L., et al., Review of the pathway for children with '? Swollen' optic discs; A service evaluation and quality improvement study. Developmental Medicine and Child Neurology, 2019. 61: p. 92.	C
134	Cohen, Y., et al., OCT imaging of papilledema in pediatric idiopathic intracranial hypertension. Investigative Ophthalmology and Visual Science, 2016. 57(12): p. 4546.	C
135	Dahlmann-Noor, A.H., et al., Ultrasonography, OCT and OCT-angiography in the diagnostic workup of children with suspected papilledema. Investigative Ophthalmology and Visual Science, 2017. 58(8).	C
136	D'Antona, L., et al., Spontaneous retinal venous pulsation: Towards non-invasive assessment of ICP. Fluids and Barriers of the CNS, 2018. 15.	C
137	D'Antona, L., et al., Spontaneous retinal venous pulsation: Towards noninvasive assessment of intracranial pressure. British Journal of Neurosurgery, 2018. 32(3): p. 336.	C
138	Duman, R., et al., Evaluation of ganglion cell layer and nerve fibre layer changes in patients with idiopathic intracranial hypertension. Neuro-Ophthalmology, 2015. 39.	C
139	Falavarjani, K.G., et al., Swept-source optical coherence tomography angiography of the optic disk in optic neuropathy. Retina, 2016. 36.	C
140	Fell, D., et al., Perfused large vessel and capillary densities in various grades of papilledema using OCTA custom software. Investigative Ophthalmology and Visual Science, 2017. 58(8).	C
141	Gelener, P., et al., Optic coherence tomography for optic disc and clinical evaluation in idiopathic intracranial hypertension. Journal of the Neurological Sciences, 2013. 333.	C
142	Gobuty, M., et al., Optical coherence tomography (OCT) use in assessing hydrocephalus. Investigative Ophthalmology and Visual Science, 2014. 55(13): p. 3362.	C
143	Gronlund, M.A., L. Mybeck, and S. Andersson, Imaging of the retina and optic nerve using optical coherence tomography in adolescents with surgically treated hydrocephalus. European Journal of Ophthalmology, 2017. 27(5).	C
144	Gupta, A., et al., First Peak fractal analysis of optical coherence tomography angiography in eyes with papilledema. Investigative Ophthalmology and Visual Science, 2018. 59(9).	C

For peer review only - http://bmjopen.bmj.com/site/about/guidelines.xhtml

145	Hacker, M., et al., Segmentation of the surfaces of the retinal layer from OCT images. Medical image computing and computer-assisted intervention : MICCAI ... International Conference on Medical Image Computing and Computer-Assisted Intervention, 2006. 9: p. 800-807.	C
146	Hajjar, D., P. Pujol, and G. Julio, Optical coherence tomography (OCT) utility in diagnosis and follow-up of papilloedema. Neuro-Ophthalmology, 2011. 35.	C
147	Homma, T.K., et al., Optical coherence tomography (OCT) as a tool to identify idiopathic intracranial hypertension in pediatric patients under GH therapy. Endocrine Reviews, 2014. 35.	C
148	Ioyleva, E. and E. Kabanova, OCT- Angiography: Perspectives of differential diagnostics of pathology optic nerve. Acta Ophthalmologica, 2018. 96: p. 55.	C
149	Ioyleva, E., E. Kabanova, and M. Krivosheeva, Measurement of macular ganglion cell-innerplexiform layer with spectral-domain optical coherence tomography in patients with optic nerve head drusen and papilledema. Acta Ophthalmologica, 2018. 96: p. 55.	C
150	Islam, M.S., et al., Deep learning based retinal blood vessel segmentation of multiple optical coherence tomography en-face images in cases of optic disc swelling. Investigative Ophthalmology and Visual Science, 2019. 60(9).	C
151	Iwase, T., et al., Serum level of vascular endothelial growth factor and opening lumbar pressure significantly correlated with optic disc edema in patients with POEMS syndrome. Investigative Ophthalmology and Visual Science, 2015. 56(7): p. 3880.	C
152	Jenkins, K.S., C.J. Layton, and M.K.M. Adams, Ophthalmoscopic and video OCT methods to detect spontaneous venous pulsation in individuals with apparently normal intracranial pressure: The rebirth of the SVP? Acta Ophthalmologica, 2017. 95.	C
153	Jensen, R. and M. Skau, Disease activity in idiopathic intracranial hypertension: A 3-month follow-up study. Journal of Headache and Pain, 2010. 11.	C
154	Jiramongkolchai, K., et al., Longitudinal Follow-Up of Papilledema by Optical Coherence Tomography Macular Segmentation. Investigative Ophthalmology & Visual Science, 2016. 57(12): p. 2.	C
155	Kapila, A., V. Lal, and A. Gupta, Clinical and neuro-ophthalmologic predictors of visual outcome in idiopathic intracranial hypertension (IIH). Journal of the Neurological Sciences, 2015. 357.	C
156	Katz, S.E., A.M. Mahmoud, and C.J. Roberts, Objective quantification of papilledema resolution after optic nerve sheath fenestration. Investigative Ophthalmology and Visual Science, 2017. 58(8).	C
157	Kedar, S., et al., Effects of acute intracranial pressure change on human and pig optic nerve head using optical coherence tomography. Investigative Ophthalmology & Visual Science, 2017. 58(8): p. 3.	C
158	Kee, H.J., S.J. Woo, and J.M. Hwang, Longitudinal change of buried optic disc drusen in children. Journal of AAPOS, 2019. 23(4).	C
159	Krauss, H.R., et al., The clinical utility of ocular coherence tomography in evaluation and management of skull base disorders. Journal of Neurological Surgery Part B: Skull Base, 2017. 78.	C

For peer review only - http://bmjopen.bmj.com/site/about/guidelines.xhtml

160	Krivosheeva, M., E. Ioyleva, and E. Kabanova, Measurement of macula thickness and macular ganglion cell with SS-OCT in patients with mild papille. Ophthalmic Research, 2018. 60: p. 14.	C
161	Kupersmith, M., R. Kardon, and M. Durbin, Retinal nerve fiber layer alteration differences between OCT and scanning laser polarimetry with optic nerve head swelling. Neurology, 2012. 78(1).	C
162	Lascaratos, G. and S. Madill, Patients With Idiopathic Intracranial Hypertension Have Significantly Larger Optic Nerve Heads Than Controls. Investigative Ophthalmology & Visual Science, 2010. 51(13): p. 2.	C
163	Lenaghan, P., et al., Point-of-care ocular sonography to detect optic disc swelling. Annals of Emergency Medicine, 2011. 58(4).	C
164	Malem, A., S. West, and G. De Salvo, Multicolor imaging in the assessment and diagnosis of optic disc swelling. Investigative Ophthalmology and Visual Science, 2015. 56(7): p. 2238.	C
165	Martinez-Alvarez, L., et al., Peripapillary choroidal neovascularization in papilloedema: Report of two cases and review of the literature. Neuro-Ophthalmology, 2015. 39.	C
166	McHugh, J., et al., Use of the heidelberg spectralis oct device to assess spontaneous venous pulsation in patients with headaches. Neuro-Ophthalmology, 2017. 41.	C
167	McNabb, R.P., et al., Widefield whole eye ssoct measurement of retinal curvature for screening of elevated intracranial pressure. Investigative Ophthalmology and Visual Science, 2018. 59(9).	C
168	Miller, E.R., C.O. Jordan, and D.L. Rogers, Optic nerve optical coherence tomography versus humphrey visual field analysis in pediatric intracranial hypertension. Journal of AAPOS, 2014. 18(4).	C
169	Miller, J.W., et al., Differentiation between papilledema and nonarteritic anterior ischemic optic neuropathy using retinal layer shape and regional volume features in spectral-domain optical coherence tomography. Investigative Ophthalmology and Visual Science, 2018. 59(9).	C
170	Monroy, J.E.G., et al., Optic nerve volumetric measurements in pediatric papilledema with spectral domain OCT. Journal of AAPOS, 2014. 18(4).	C
171	Morrow, M., F. Villanueva, and F. Abukhalil, Evolution of macular findings on optical coherence tomography (OCT) in papilledema. Neurology, 2014. 82(10).	C
172	Morrow, M., The relationship between optical coherence tomographic (OCT) and perimetric findings in patients with papilledema. Neurology, 2014. 82(10).	C
173	Morrow, M., The relationship between optical coherence tomographic and perimetric findings in patients with papilledema. Journal of the Neurological Sciences, 2013. 333.	C
174	Moss, H., et al., Optic nerve head geometry as a function of chronic intracranial pressure. Investigative Ophthalmology and Visual Science, 2017. 58(8).	C
175	Moss, H., J.J. McAnany, and J.C. Park, Structure function relationships in idiopathic intracranial hypertension. Investigative Ophthalmology and Visual Science, 2015. 56(7): p. 2230.	C
176	Nayfach, J., et al., Integrating funduscopy, optical coherence tomography (OCT), and ultrasound to differentiate optic disc edema (ODE) from pseudo optic disc edema (PODE). Investigative Ophthalmology and Visual Science, 2016. 57(12): p. 4541.	C

For peer review only - http://bmjopen.bmj.com/site/about/guidelines.xhtml

177	Pak, K., et al., Anatomical and functional changes of optic disc and retinal nerve fiber layer after bariatric surgery in morbidly obese Korean patients with lower BMI. Investigative Ophthalmology and Visual Science, 2019. 60(9).	C
178	Parhiz, A.T., P. Sibony, and M. Kupersmith, SD-OCT in monitoring intracranial pressure in patients with CSF shunts. Investigative Ophthalmology and Visual Science, 2013. 54(15).	C
179	Pascoe, E., et al., A case series of intracranial hypertension in patients with Turner syndrome, with and without growth hormone therapy. International Journal of Pediatric Endocrinology, 2015. 2015.	C
180	Pathmanathan, N., et al., Audit of intracranial pressure monitoring in traumatic brain injury. Intensive care medicine, 2013. 39: p. S259-.	C
181	Pemp, B., et al., Changes of choroidal thickness as measured with spectral domain OCT in patients with idiopathic intracranial hypertension after lowering of intracranial pressure. Investigative Ophthalmology and Visual Science, 2013. 54(15).	C
182	Pemp, B., et al., Measurements by spectral domain oct in papilledema are associated with reduced retinal nerve fiber layer thickness after resolution of optic Disc swelling. Investigative Ophthalmology and Visual Science, 2014. 55(13): p. 5779.	C
183	Pemp, B., et al., The course of divergence paresis and papilloedema in patients with increased intracranial pressure. Neuro-Ophthalmology, 2015. 39.	C
184	Permeswaran, Y.M., et al., An en-face OCT registration approach for locating the center of bruch's membrane opening in longitudinal papilledema cases. Investigative Ophthalmology and Visual Science, 2019. 60(9).	C
185	Pilat, A., et al., Macula structure in patients with optic disc oedema and optic nerve head drusen. Investigative Ophthalmology and Visual Science, 2013. 54(15).	C
186	Reitner, A., B. Pemp, and K. Kircher, Retinal nerve fibre layer thickness measurement using spectral domain OCT in various optic nerve lesions. Neuro-Ophthalmology, 2011. 35.	C
187	Sarrafpour, S., et al., Fractal analysis of peripapillary vasculature in eyes with papilledema using optical coherence tomography angiography. Investigative Ophthalmology and Visual Science, 2017. 58(8).	C
188	Scott, L.L., et al., Correlation of opening pressure and Frisen grade of papilledema in pediatric patients with intracranial hypertension. Investigative Ophthalmology and Visual Science, 2015. 56(7): p. 2231.	C
189	Skau, M., et al., Diagnostic value of optical coherence tomography for intracranial pressure in idiopathic intracranial hypertension. Journal of Headache and Pain, 2013. 14(1).	C
190	Stark, R.M., et al., Assessment of intracranial pressure during venous sinus stenting. Journal of Neurological Surgery, Part B: Skull Base, 2016. 77.	C
191	Starke, R.M., et al., A prospective pilot study of intraparenchymal & intravenous cerebral pressure assessment during venous sinus stenting. Journal of Neurosurgery, 2016. 124(4).	C
192	Stroet, A., et al., Optical coherence tomography in idiopathic intracranial hypertension-first experiences. Neuropediatrics, 2012. 43(2).	C
193	Su, Q.Y., et al., Spectral-Domain Optical Coherence Tomography Optic-Nerve-Head and Macular En-Face Image Registration in Cases of	C

For peer review only - http://bmjopen.bmj.com/site/about/guidelines.xhtml

	Papilledema. Investigative Ophthalmology & Visual Science, 2017. 58(8): p. 3.	
194	Swinkin, E., A. Sundaram, and J.M. Pejman, Unilateral papilledema in idiopathic intracranial hypertension: A report of two cases and literature review. Neurology, 2017. 88(16).	C
195	Tang, R., et al., Optical coherence tomography visualization of optic nerve head structure detects acute changes in the intracranial pressure. Neurology, 2014. 82(10).	C
196	Thompson, D.A., et al., Outsized paediatric VEPs. Documenta Ophthalmologica, 2017. 135(1): p. 24.	C
197	Van De Beeten, S.D.C., et al., Increased total retinal thickness on OCT: A precursor for intracranial hypertension? Child's Nervous System, 2018. 34(5): p. 1006-1007.	C
198	Vangipuram, G. and H. Moss, Changes in basement membrane opening displacement within 1 hour following intracranial pressure lowering in subjects with and without idiopathic intracranial hypertension. Investigative Ophthalmology and Visual Science, 2015. 56(7): p. 2232.	C
199	Varma, H., et al., Longitudinal analysis of optic nerve edema, retinal nerve fiber layer thickness, and visual field mean deviation in pediatric patients with intracranial hypertension. Journal of AAPOS, 2019. 23(4).	C
200	Wall, M., et al., Detection of visual loss in IIH with static automated perimetry: Temporal wedge defects. Investigative Ophthalmology and Visual Science, 2019. 60(9).	C
201	Wan, M.J., Correlation between spectral domain optical coherence tomography (SD-OCT) and long-term vision loss in children with papilledema. Journal of AAPOS, 2019. 23(4).	C
202	Wang, J.K., et al., Automated 3-D Region-Based Volumetric Estimation of Optic Disc Swelling in Papilledema Using Spectral-Domain Optical Coherence Tomography, in Medical Imaging 2013: Biomedical Applications in Molecular, Structural, and Functional Imaging, J.B. Weaver and R.C. Molthen, Editors. 2013, Spie-Int Soc Optical Engineering: Bellingham.	C
203	Wang, J.K., et al., Automated Classification of Retinal Folds and Wrinkles in En-Face Optical Coherence Tomography Images with Optic Disc Swelling. Investigative ophthalmology & visual science, 2019. 60(9).	C
204	Wang, J.K., et al., Automated detection of retinal folds in papilledema using en-face spectral-domain optical coherence tomography. Investigative Ophthalmology and Visual Science, 2018. 59(9).	C
205	Wang, J.K., et al., Change in the deflection of the neural canal opening over time with acetazolamide treatment in idiopathic intracranial hypertension. Investigative Ophthalmology and Visual Science, 2015. 56(7): p. 2233.	C
206	Wang, J.K., et al., Regional spectral-domain optical coherence tomography features better predict Frisen scale grades than total volume alone in papilledema. Investigative Ophthalmology and Visual Science, 2013. 54(15).	C
207	Wang, J.K., et al., Semi-Automated 2D Bruch's Membrane Shape Analysis in Papilledema Using Spectral-Domain Optical Coherence Tomography, in Medical Imaging 2015: Biomedical Applications in Molecular, Structural, and Functional Imaging, B. Gimi and R.C. Molthen, Editors. 2015, Spie-Int Soc Optical Engineering: Bellingham.	C

For peer review only - http://bmjopen.bmj.com/site/about/guidelines.xhtml

208	Wang, J.K., R.H. Kardon, and M.K. Garvin, Combined Use of High-Definition and Volumetric Optical Coherence Tomography for the Segmentation of Neural Canal Opening in Cases of Optic Nerve Edema, in Medical Imaging 2015: Image Processing, S. Ourselin and M.A. Styner, Editors. 2015, Spie-Int Soc Optical Engineering: Bellingham.	C
209	Weaver, M.A., et al., Deformation of the peripapillary retinal pigmented epithelium layer on OCT: a useful tool to identify intracranial hypertension in a pediatric population? Journal of AAPOS, 2019. 23(4).	C
210	Yri, H., et al., EHMTI-0155. The course of headache in idiopathic intracranial hypertension: A 12 month prospective follow-up study. Journal of Headache and Pain, 2014. 15.	C
211	Yri, H., et al., Idiopathic intracranial hypertension is not benign; a prospective long-term follow-up study. Journal of Headache and Pain, 2013. 14(1).	C
212	Kleinberg, T.T. and J.R. Bilyk, Evolution of Disc Edema in Thyroid-Related Compressive Optic Neuropathy. Ophthalmic plastic and reconstructive surgery, 2016. 32(4): p. e100.	D
213	Perez-Lopez, M., D.S. Ting, and L. Clarke, Lamina cribrosa displacement after optic nerve sheath fenestration in idiopathic intracranial hypertension: a new tool for monitoring changes in intracranial pressure? Br J Ophthalmol, 2014. 98(11): p. 1603-4.	D
214	Rezkallah, A., et al., Hypertensive choroidopathy: Multimodal imaging and the contribution of wide-field swept-source oct-angiography. American journal of ophthalmology case reports, 2019. 13: p. 131-135.	D
215	Santos-Bueso, E., et al., [Idiopathic intracranial hypertension. Analysis by macular segmentation]. An Pediatr (Barc), 2015. 82(5): p. e230-3.	D
216	Shuaib, M.M., et al., Optical coherence tomography of the optic nerve head before and after optic nerve sheath fenestration for idiopathic intracranial hypertension. Acta neurologica Belgica, 2020.	D
217	Sibony, P.A. and M.J. Kupersmith, Paton's Folds revisited: Wrinkles, folds and creases in papilledema. Investigative Ophthalmology and Visual Science, 2016. 57(12): p. 4553.	D
218	Fard, M.A., Bruch's membrane opening on optical coherence tomography in pediatric papilledema and pseudopapilledema. Journal of AAPOS : the official publication of the American Association for Pediatric Ophthalmology and Strabismus, 2018. 22(3): p. 247.	E
219	García-Montesinos, J., et al., Bruch's membrane opening on optical coherence tomography in pediatric papilledema and pseudopapilledema. J aapos, 2018. 22(3): p. 247-248.	E
220	Ophir, A., Optical coherence tomography as an adjunctive tool for diagnosing pediatric pseudotumor cerebri. Journal of AAPOS, 2008. 12(4): p. 421-422.	E
221	Savini, G., et al., Optical coherence tomography for optic disc edema. Archives of Ophthalmology, 2011. 129(9): p. 1245-1246.	E

For peer review only - http://bmjopen.bmj.com/site/about/guidelines.xhtml

PRISMA 2009 Checklist

Section/topic	#	Checklist item	Reported on page #
**TITLE**			
Title	1	Identify the report as a systematic review, meta-analysis, or both.	1
**ABSTRACT**			
Structured summary	2	Provide a structured summary including, as applicable: background; objectives; data sources; study eligibility criteria, participants, and interventions; study appraisal and synthesis methods; results; limitations; conclusions and implications of key findings; systematic review registration number.	2
**INTRODUCTION**			
Rationale	3	Describe the rationale for the review in the context of what is already known.	3
Objectives	4	Provide an explicit statement of questions being addressed with reference to participants, interventions, comparisons, outcomes, and study design (PICOS).	3-4
**METHODS**			
Protocol and registration	5	Indicate if a review protocol exists, if and where it can be accessed (e.g., Web address), and, if available, provide registration information including registration number.	4
Eligibility criteria	6	Specify study characteristics (e.g., PICOS, length of follow-up) and report characteristics (e.g., years considered, language, publication status) used as criteria for eligibility, giving rationale.	4
Information sources	7	Describe all information sources (e.g., databases with dates of coverage, contact with study authors to identify additional studies) in the search and date last searched.	4
Search	8	Present full electronic search strategy for at least one database, including any limits used, such that it could be repeated.	4
Study selection	9	State the process for selecting studies (i.e., screening, eligibility, included in systematic review, and, if applicable, included in the meta-analysis).	4
Data collection process	10	Describe method of data extraction from reports (e.g., piloted forms, independently, in duplicate) and any processes for obtaining and confirming data from investigators.	4
Data items	11	List and define all variables for which data were sought (e.g., PICOS, funding sources) and any assumptions and simplifications made.	4
Risk of bias in individual studies	12	Describe methods used for assessing risk of bias of individual studies (including specification of whether this was done at the study or outcome level), and how this information is to be used in any data synthesis.	4
Summary measures	13	State the principal summary measures (e.g., risk ratio, difference in means).	4
Synthesis of results	14	Describe the methods of handling data and combining results of studies, if done, including measures of consistency (e.g., I^2) for each meta-analysis.	N/A

For peer review only

For peer review only - http://bmjopen.bmj.com/site/about/guidelines.xhtml

PRISMA 2009 Checklist

Section/topic	#	Checklist item	Reported on page #
Risk of bias across studies	15	Specify any assessment of risk of bias that may affect the cumulative evidence (e.g., publication bias, selective reporting within studies).	4
Additional analyses	16	Describe methods of additional analyses (e.g., sensitivity or subgroup analyses, meta-regression), if done, indicating which were pre-specified.	N/A
**RESULTS**			
Study selection	17	Give numbers of studies screened, assessed for eligibility, and included in the review, with reasons for exclusions at each stage, ideally with a flow diagram.	5
Study characteristics	18	For each study, present characteristics for which data were extracted (e.g., study size, PICOS, follow-up period) and provide the citations.	5-7
Risk of bias within studies	19	Present data on risk of bias of each study and, if available, any outcome level assessment (see item 12).	5-7
Results of individual studies	20	For all outcomes considered (benefits or harms), present, for each study: (a) simple summary data for each intervention group (b) effect estimates and confidence intervals, ideally with a forest plot.	5-7
Synthesis of results	21	Present results of each meta-analysis done, including confidence intervals and measures of consistency.	N/A
Risk of bias across studies	22	Present results of any assessment of risk of bias across studies (see Item 15).	5
Additional analysis	23	Give results of additional analyses, if done (e.g., sensitivity or subgroup analyses, meta-regression [see Item 16]).	N/A
**DISCUSSION**			
Summary of evidence	24	Summarize the main findings including the strength of evidence for each main outcome; consider their relevance to key groups (e.g., healthcare providers, users, and policy makers).	9-10
Limitations	25	Discuss limitations at study and outcome level (e.g., risk of bias), and at review-level (e.g., incomplete retrieval of identified research, reporting bias).	9-10
Conclusions	26	Provide a general interpretation of the results in the context of other evidence, and implications for future research.	11
**FUNDING**			
Funding	27	Describe sources of funding for the systematic review and other support (e.g., supply of data); role of funders for the systematic review.	12

From: Moher D, Liberati A, Tetzlaff J, Altman DG, The PRISMA Group (2009). Preferred Reporting Items for Systematic Reviews and Meta-Analyses: The PRISMA Statement. PLoS Med 6(7): e1000097. doi:10.1371/journal.pmed1000097

For more information, visit: **www.prisma-statement.org**.

For peer review only - http://bmjopen.bmj.com/site/about/guidelines.xhtml

BMJ Open

Detection of intracranial hypertension in children using optical coherence tomography: a systematic review

Journal:	*BMJ Open*
Manuscript ID	bmjopen-2020-046935.R1
Article Type:	Original research
Date Submitted by the Author:	28-Jun-2021
Complete List of Authors:	Rufai, Sohaib; University of Leicester, Ulverscroft Eye Unit; Great Ormond Street Hospital for Children, Craniofacial Unit<br>Hisaund, Michael; University of Leicester, Neuroscience, Psychology and Behaviour<br>Jeelani, Noor ul Owase; Great Ormond Street Hospital for Children, Craniofacial Unit<br>McLean, Rebecca; University of Leicester, Ulverscroft Eye Unit
**Primary Subject Heading**:	Ophthalmology
Secondary Subject Heading:	Paediatrics, Neurology, Surgery, Research methods, Patient-centred medicine
Keywords:	Neuro-ophthalmology < OPHTHALMOLOGY, Paediatric ophthalmology < OPHTHALMOLOGY, Paediatric neurology < NEUROLOGY, Neurological oncology < NEUROLOGY, Paediatric neurosurgery < PAEDIATRIC SURGERY, PLASTIC & RECONSTRUCTIVE SURGERY

SCHOLARONE™
Manuscripts

I, the Submitting Author has the right to grant and does grant on behalf of all authors of the Work (as defined in the below author licence), an exclusive licence and/or a non-exclusive licence for contributions from authors who are: i) UK Crown employees; ii) where BMJ has agreed a CC-BY licence shall apply, and/or iii) in accordance with the terms applicable for US Federal Government officers or employees acting as part of their official duties; on a worldwide, perpetual, irrevocable, royalty-free basis to BMJ Publishing Group Ltd ("BMJ") its licensees and where the relevant Journal is co-owned by BMJ to the co-owners of the Journal, to publish the Work in this journal and any other BMJ products and to exploit all rights, as set out in our licence.

The Submitting Author accepts and understands that any supply made under these terms is made by BMJ to the Submitting Author unless you are acting as an employee on behalf of your employer or a postgraduate student of an affiliated institution which is paying any applicable article publishing charge ("APC") for Open Access articles. Where the Submitting Author wishes to make the Work available on an Open Access basis (and intends to pay the relevant APC), the terms of reuse of such Open Access shall be governed by a Creative Commons licence – details of these licences and which Creative Commons licence will apply to this Work are set out in our licence referred to above.

Other than as permitted in any relevant BMJ Author's Self Archiving Policies, I confirm this Work has not been accepted for publication elsewhere, is not being considered for publication elsewhere and does not duplicate material already published. I confirm all authors consent to publication of this Work and authorise the granting of this licence.

For peer review only

Title Page

Title: Detection of intracranial hypertension in children using optical coherence tomography: a systematic review

Authors:

Dr Sohaib R. Rufai[1-3] (BMBS, MRes), Mr Michael Hisaund[1] (BMedSci), Dr Noor ul Owase Jeelani[3] (MPhil, FRCS), Dr Rebecca J. McLean[1] (MSc, PhD)

Affiliations:

1. The University of Leicester Ulverscroft Eye Unit, Robert Kilpatrick Clinical Sciences Building, Leicester Royal Infirmary, Leicester, UK.
2. Clinical and Academic Department of Ophthalmology, Great Ormond Street Hospital for Children NHS Foundation Trust, London, UK.
3. UCL Great Ormond Street Institute of Child Health, London, UK & Craniofacial Unit, Great Ormond Street Hospital for Children NHS Foundation Trust, London, WC1N 3JH, United Kingdom

Corresponding Author: Dr Rebecca J. McLean (MSc, PhD); Address: The University of Leicester Ulverscroft Eye Unit, Robert Kilpatrick Clinical Sciences Building, PO Box 65, Leicester LE2 7LX, UK; E-mail: rjm19@leicester.ac.uk; Telephone: +44 (0)116 2523152; Fax: +44 (0)116 2231996

Keywords: Intracranial pressure; paediatric intracranial hypertension; optical coherence tomography; early recognition; systematic review.

Funding: The Medical Research Council (London, UK) (grant no.: MR/N004566/1). SRR is funded by a National Institute for Health Research (NIHR) Doctoral Fellowship for this research project. This paper presents independent research funded by the National Institute for Health Research (NIHR) and MRC. The views expressed are those of the author(s) and not necessarily those of the MRC, the NHS, the NIHR or the Department of Health and Social Care.

Competing interests: The authors have no proprietary or commercial interest in any materials discussed in this study.

For peer review only

Abstract

Objectives: To evaluate the diagnostic capability of optical coherence tomography (OCT) in children aged under 18 years old with intracranial hypertension (IH).

Design: Systematic review

Methods: We conducted a systematic review using the following platforms to search the keywords 'optical coherence tomography' and 'intracranial hypertension' from inception until 2nd April 2020: CENTRAL, EMBASE, MEDLINE, PubMed and Web of Science, without language restrictions. Our search returned 2729 records, screened by two independent screeners. Studies were graded according to the Oxford Centre for Evidence-Based Medicine and National Institutes of Health Quality Assessment Tool for observational studies.

Results: 21 studies were included. Conditions included craniosynostosis (n=354 patients), idiopathic intracranial hypertension (IIH; n=102), space occupying lesion (SOL; n=42) and other pathology (n=29). OCT measures included optic nerve parameters, rim parameters (notably retinal nerve fibre layer thickness) and retinal parameters. Levels of evidence included 2b (n=13 studies), 3b (n=4) and 4 (n=4). Quality of 10 studies was fair and 11 poor. There was inconsistency in OCT parameters and gold standard measures studied, albeit OCT did demonstrate good diagnostic capability for IH in craniosynostosis, IIH and SOL.

Conclusions: This systematic review identified various studies involving OCT to assist diagnosis and management of IH in children with craniosynostosis, IIH and SOL, in conjunction with established clinical measures of intracranial pressure. However, no level 1 evidence was identified. Validating prospective studies are therefore required to determine optimal OCT parameters in this role and to develop formal clinical guidelines.

PROSPERO registration number: CRD42019154254

Strengths and limitations of this study

- This is the first systematic review to evaluate the role of optical coherence tomography (OCT) in paediatric intracranial hypertension (IH).
- This systematic review was conducted in accordance with rigorous Cochrane methodology.
- A broad search strategy was employed, without date or language restrictions, to identify all relevant evidence.
- There was inconsistency in evidence levels and quality across the included studies, albeit OCT did demonstrate good diagnostic capability for paediatric IH.
- Validating prospective studies are required to develop formal clinical guidelines for OCT in paediatric IH.

For peer review only - http://bmjopen.bmj.com/site/about/guidelines.xhtml

Text

Introduction

Intracranial hypertension (IH) was first described by Quincke in 1896 and remains a subject of major clinical importance.[1] IH affects between 0.63 and 0.71 per 100,000 children.[2, 3] Unaddressed IH can inflict devastating sequelae including visual impairment, neurocognitive delay, disability and death.[4, 5] Subacute pathology in children can cause insidious IH which may pose deleterious effects on the brain and vision before clinical detection. Thus, prompt detection and timely intervention is key in preventing or limiting the sequelae of IH.

Assessment of intracranial pressure (ICP) in children is notoriously difficult. Direct intraparenchymal measurement represents the gold standard, but carries numerous disadvantages including the need for overnight hospital admission, general anaesthesia and significant risk.[6, 7] An ideal screening method would be sensitive, specific, safe, highly reproducible, rapid, non-invasive and child friendly with the capability to record serial measurements. Existing measures fail to fully satisfy all these criteria and often yield equivocal results in young children, including fundus examination[8], B-scan ocular ultrasound[9, 10], radiology[11] and visual evoked potentials.[12, 13]

IH causes optic nerve and retinal changes, which can be detected and quantified using optical coherence tomography (OCT) – a non-invasive imaging method to acquire ultra-high resolution cross-sectional images of the optic nerve and retina within seconds.[14] OCT has been successfully used to study the normal and abnormal development of the optic nerve[15] and fovea[16] in children, plus various conditions associated with IH.[17-23] Here, we conducted a systematic review to assess the role of OCT in detecting IH in children.

For peer review only - http://bmjopen.bmj.com/site/about/guidelines.xhtml

Methods

This systematic review was conducted in accordance with PRISMA guidelines[24] and the Cochrane Handbook.[25] The protocol is registered on PROSPERO[26] (CRD42019154254) and published in *BMJ Open*[27].

Eligibility criteria for considering studies for this review

Eligibility criteria were established *a priori* and included OCT studies of children (aged under 18 years) with intracranial hypertension. Level 4 evidence and above was included, as per the Oxford Centre for Evidence-based Medicine (CEBM).[28] Exclusion criteria were : i) adult studies aged 18 or over; ii) studies not pertaining to IH; iii) studies not using OCT; iv) case reports and expert opinion without critical appraisal.

Search methods for identifying studies

Medical subject headings (MeSH) terms for 'optical coherence tomography' and 'intracranial hypertension' were entered into search platforms: Cochrane Central Register of Controlled Trials (CENTRAL), MEDLINE, EMBASE, PubMed and Web of Science. Appendix 1 contains full details of our search terms and strategy. EndNote X9 (Thomson Reuters, New York, NY) software was used to manage data. No date/language restrictions were stipulated.

Study selection

A three-stage, independent screening process was employed by two screeners (SRR and RJM), involving eligibility screening of titles, abstracts and full papers. Screening questions are listed in Appendix 2.

Data collection and quality assessment

The main outcome measure was the diagnostic capability for OCT in detecting IH, expressed as diagnostic accuracy or by appropriate statistical testing.

Secondary outcome measures were:

- Condition(s) associated with IH per study;
- OCT device(s) used;
- OCT success rate;
- Other surrogate estimates of ICP;
- ICP range determined as normal.

Our data extraction tool was adapted from the Cochrane Collaboration (Appendix 3).[29] Evidence levels were graded by two independent graders (SRR and MH) as per the Oxford CEBM[28] and the National Institutes of Health (NIH) Quality Assessment Tool for Observational Studies[30] was applied for individual study quality grading (Appendix 4).

For peer review only

Patient and public involvement

There was no patient and public involvement specific to this systematic review. However, this group has commenced prospective research using handheld OCT in paediatric IH featuring substantial patient and public involvement, which will be reported separately.

For peer review only

For peer review only - http://bmjopen.bmj.com/site/about/guidelines.xhtml

Results

Our search was executed on April 2nd 2020 and data extraction completed on 12th July 2020. Our search returned 2729 records in total, 1665 following de-duplication. Following full-text screening, 21 studies were eligible for inclusion in our review (Figure 1). Supplementary Table 1 contains the list of excluded articles with reasons. Table 1 summarises the study characteristics and quality assessment of the 21 included studies. One study[31] was written in Polish and translated by an interpreter, whilst the remaining 20 were written in English. Nine studies were prospective[22, 23, 32-38] while twelve were retrospective.[31, 39-49] No randomised controlled trials (RCTs) or systematic reviews were eligible for inclusion. Following review of the included studies, it was deemed inappropriate to perform quantitative synthesis due to inconsistency in study design and methodology, particularly OCT parameters and gold standard measures of ICP. Therefore, qualitative synthesis was performed.

This review identified studies of craniosynostosis (n=354 patients), idiopathic intracranial hypertension (IIH; n=102), space occupying lesion (SOL; n=42) and other pathologies (n=29) associated with risk of IH. OCT measures in these studies included optic nerve parameters, rim parameters and retinal parameters. Main outcome measures are displayed in Table 2. Secondary outcome measures are reported per condition.

Evidence summary: craniosynostosis

Craniosynostosis is characterised by the premature, pathological fusion of one or more cranial sutures. This restriction in skull growth can cause IH. Six studies in this review utilised OCT in patients with craniosynostosis.[22, 23, 33, 34, 40, 49] These studies included a total of 393 participants, of which 354 were diagnosed with craniosynostosis[22, 23, 33, 34, 40, 49]. In one comparative study[22], 5 positive controls had hydrocephalus and suspected IH[22], while 34 were normal controls.[22] OCT devices used include the iVue[22, 23] (Optovue, Fremont, CA, US; software version 3.2) and Spectralis[33, 34, 40, 49] (Heidelberg Engineering, GmbH, Heidelberg, Germany).

OCT parameters evaluated include retinal nerve fibre layer (RNFL) thickness[22, 23, 40], maximal retinal thickness[22, 23, 33, 34, 49] and anterior retinal projection.[22, 23] OCT parameters demonstrated good diagnostic capability for IH in craniosynostosis (Table 2). Increased RNFL thickness, maximal retinal thickness and anterior retinal projection were associated with papilloedema, while RNFL thinning was associated with optic atrophy. Using single, on-table ICP measures, Swanson *et al*[22] demonstrated good sensitivity (89%) and specificity (62%) of combined RNFL thickness and maximal anterior retinal projection in detecting IH (Figure 2). They found that maximal RNFL thickness exceeding 207µm or maximal anterior retinal projection exceeding 159µm in either eye corresponded to the 97.5th percentile of healthy control patients, thereby representing IH; these figures did not vary significantly based on age.[22]

Reported OCT imaging success rates were high. Driessen *et al*[34] reported overall OCT success in 85% of eyes. Dagi *et al*[40] did not specify success rate, but reported that 16.9% were excluded due to limited cooperation, severe nystagmus, poor scan quality or retinal degeneration. Swanson *et al* implied 100% success rate in both studies[22, 23], but acquired OCT images under general anaesthesia and therefore were non-reliant on patient cooperation. Other studies of craniosynostosis did not report OCT success rates.

For peer review only - http://bmjopen.bmj.com/site/about/guidelines.xhtml

Apart from OCT, other surrogate measures of ICP displayed poor sensitivity, limiting their potential as screening tools for IH when used in isolation. These included fundoscopy[22, 23, 33, 40], visual acuity[40], visual fields[40], radiological signs.[22, 23, 40] and clinical history including complaints of headache.[22, 23]

With respect to defining IH, there is no universally agreed clinical consensus on timing, frequency and duration for accurate ICP measurement, or indeed what figure constitutes raised ICP.[50] In the three studies using ICP measurements[22, 33, 49], ICP <10 mmHg was determined as normal, while 10-15 mmHg was determined as borderline and >15 mmHg was determined as raised.

Evidence summary: idiopathic intracranial hypertension

IIH, or primary IH, is characterised by raised ICP in the context of normal CSF composition and no evidence of SOL or ventriculomegaly on neuroimaging.[14] Principles from the modified Dandy criteria[51] and the revised criteria by Friedman *et al*[52] can assist in making diagnosis without ambiguity. Eight studies in this review utilised OCT in patients with IIH.[35, 36, 42, 43, 45-48] These studies included a total of 237 participants, of which 102 were diagnosed with IIH[35, 36, 42, 43, 45-48]. Of the other included participants in these studies, 74 had pseudopapilledema,[43, 46, 48] 3 had SOL[43], 6 had other pathology[43] covered below and 52 were normal controls.[35, 46, 48] OCT devices used include the Cirrus HD-OCT[14] (Carl Zeiss Meditec, Dublin, CA, US), Spectralis[42, 48] and Stratus[35, 47] (Carl Zeiss Meditec, Dublin, CA, US). Three studies did not specify which OCT device was used.[43, 45, 46]

OCT parameters included RNFL thickness[35, 36, 42, 47, 48], macular volume[35], disruption of the ellipsoid zone[42] and Bruch's membrane opening.[48] Two studies did not specify which OCT parameters were used.[45, 46] OCT demonstrated good diagnostic capability in IIH (Table 2). Increased RNFL thickness[35, 36, 43, 48] and macular volume[35] were associated with IH, while RNFL thinning and disruption of the ellipsoid zone were associated with optic atrophy and vision loss.[42] In addition, Thompson *et al*[48] found that the transverse diameter of Bruch's membrane opening was enlarged in mild papilloedema and could be used together with RNFL thickness to distinguish mild papilloedema from psuedopapilloedema (Figure 3).

Lee *et al*[36] reported 86.7% OCT imaging success rate, while Sanches-Tocino *et al*[47] reported 100% OCT success. Other studies of IIH did not report success rates.

With regards to other surrogate measures of ICP, Ozturk et al[46] found that optic nerve sheath diameter associated moderately with CSF opening pressure ($r=0.661$; $p<0.005$). Lee et al[36] found that body mass index (BMI) associated moderately with lumbar puncture opening pressure ($r=0.607$; $p=0.028$). Headache characteristics did not reliably detect children with IIH.[35, 36, 45-47]

With respect to ICP measurements, only Krishnakumar *et al*[45] provided definitions for normal and raised ICP, using lumbar CSF opening and steady state pressures: <15 mmHg = normal CSF pressure; >20 mmHg = high pressure.

Evidence summary: space occupying lesion

Intracranial SOL include tumours or abscesses within the cranial cavity, which are associated with IH. Five studies in this review utilized OCT in patients with SOL.[37, 39, 41, 43, 44] These studies included a total of 121 participants, of which 42 had SOL[37, 39, 41, 43, 44]. Of the remaining participants, 2 had IIH[43], 9 had psuedopapilloedema[43], 10 had other pathology[37, 41, 43] covered below and 58 had optic disc drusen

For peer review only - http://bmjopen.bmj.com/site/about/guidelines.xhtml

with no intracranial pathology.[41] The following OCT devices were used: Cirrus HD-OCT[39, 44], DRI-OCT-1 Atlantis[37], Spectralis[41] and Stratus[39]. Malem *et al*[43] did not specify which device was used.

OCT parameters included RNFL thickness[39, 41, 43, 44], ganglion cell layer thickness[44], anterior bowing of Bruch's membrane[41], neural canal diameter[37], papillary vertical height[37] and anterior lamina cribrosa depth.[37] OCT demonstrated value as part of the clinical work-up of these children (Table 2). RNFL thinning was associated with optic atrophy[39] and visual field loss[39, 44], as was ganglion cell loss.[44] Lee *et al*[37] highlighted the reversibility of papilloedema in patients with SOL following ICP reducing surgery, whereby mean neural canal diameter and papillary vertical height decreased whilst mean anterior lamina cribrosa depth increased (Figure 4). Dahlmann-Noor *et al*[41] reported one patient with intraventricular tumour with increased temporal RNFL thickness, but no bowing of Bruch's membrane.

Dahlmann-Noor *et al*[41] reported OCT imaging success rate of 100%, while other SOL studies did not report success rate.

Clinical history including headache characteristics did not reliably detect children with SOL.[39, 41] Humphrey visual field testing is not designed for children and was difficult or not feasible in many cases.[39, 44]

Lee *et al*[37] reported mean preoperative and postoperative ICP values of 24.0±5.0 and 13.2±6.3 mmHg, but no SOL study defined a normal range for ICP.[39, 41]

Evidence summary: other pathology

Seven studies[22, 31, 32, 37, 38, 41, 43] included children with other pathology associated with IH, including hydrocephalus[22, 37, 38] (n=16), papilloedema of unspecified aetiology[32] (n=5), acute lymphoblastic leukaemia[41] (n=1), aseptic meningitis[43] (n=1), growth hormone replacement therapy[31] (n=1), head injury[43] (n=1), recurrent nephrotic syndrome[31] (n=1), rickets[41] (n=1), sagittal sinus thrombosis[43] (n=3) and sigmoid sinus thrombosis[43] (n=1). Of note, Tran-Viet *et al*[38] used the Envisu C2300 (Bioptigen, Inc, Research Triangle Park, NC) – a handheld OCT device, with which they successfully scanned 25/26 (96%) undilated eyes of conscious infants without sedation. Other OCT devices used were the Spectralis and DRI-OCT-1 Atlantis; Malem *et al*[43] did not specify which device was used.

OCT parameters used in these studies include RNFL thickness[31, 32, 41, 43], volumetric spectral domain (SD) OCT of optic nerve head[32], volumetric enhanced depth imaging (EDI) OCT of optic nerve head [32], neural canal diameter[37], papillary vertical height[37], anterior lamina cribrosa depth[37], and anterior bowing of Bruch's membrane[41] (Table 2). Tran-Viet *et al*[38] did not specify which OCT parameters were used. Chang *et al*[32] found a positive association between optic nerve head volume on SD-OCT and EDI-OCT with papilloedema, but did not specify aetiology. Other associations between OCT parameters and IH have been described above.

OCT imaging success rates were high in these studies: Tran-Viet *et al*[38] reported 96% success, Chang *et al*[32] reported 98% and Dahlmann-Noor *et al*[41] reported 100%. Mrugacz *et al*[31] and Swanson *et al*[22, 23] implied 100% success rates. Other studies did not report OCT success rates.

For peer review only - http://bmjopen.bmj.com/site/about/guidelines.xhtml

Discussion

This systematic review collated a body of evidence evaluating the role of OCT to detect IH, specifically the structural changes associated with papilloedema and optic atrophy. This review could not recommend the widespread use of OCT in all children at risk of IH as standard clinical practice, as no level 1 studies were identified for these conditions. However, this review recognises the value of OCT in paediatric IH, particularly in cases where ICP status is uncertain or borderline, for a number of reasons: i) OCT enables rapid, non-invasive, quantitative cross-sectional measurements of the optic nerve head and retina, not offered by conventional fundoscopy; ii) OCT permits serial measurements to help appreciate evolution in optic nerve head and retinal changes over time, where applicable; iii) OCT could guide and support the overall clinical work-up of affected children. Further prospective validating studies are required to develop formal clinical guidelines for OCT in this role.

Quality of Evidence

Two independent graders (SRR and MH) identified varying levels of evidence among the 21 included studies, as per the Oxford CEBM.[28] There were no systematic reviews, RCTs or validating cohort studies measuring OCT parameters against established reference standards, hence no study was graded as level 1 evidence. The NIH Quality Assessment Tool for individual studies identified 10 studies of fair quality and 11 studies of poor quality (Table 1). The major limiting factor to quality was use of other surrogate clinical measures as the gold standard for IH, as opposed to direct intraparenchymal ICP monitoring.

Research in context

There is a paucity of clinical guidelines for the management of pediatric IH in general. Searches for relevant clinical guidelines were performed via PubMed using the search terms "guidelines" AND "optical coherence tomography" AND "idiopathic intracranial hypertension" OR "craniosynostosis" OR "space occupying lesion", which returned two relevant guidelines. Mollan *et al*[53] recently published the first consensus guidelines on management of IIH, which state: "Where visual function is found to be threatened, regular ophthalmic examination must occur because this will influence timely management… Formal documentation of the optic nerve head appearance, such as serial photographs or OCT imaging, is useful." This is consistent with our review's findings. The guidelines by Mollan *et al*[53] apply to all patients with IIH and are not specific to children. The Working Group on Craniosynostosis[54] published clinical guidelines for craniosynostosis in 2015, but these did not feature OCT. No relevant guidelines were identified for the use of OCT in SOL. The same search strategy was used for nice.org.uk - the UK's National Institute for Health and Care Excellence, which returned no relevant guidelines.

The NORDIC Idiopathic Intracranial Hypertension Treatment Trial (IIHTT) is a landmark RCT with a dedicated OCT sub-study committee.[55] Although the resulting papers appeared in our systematic search, they were excluded from our review because they excluded children aged under 18. On further reading, a positive association between ICP and RNFL thickness, total retinal thickness and optic nerve head volume was also found, consistent with our review findings.[55]

This review identified studies using a range of OCT devices. Conventional, table-mounted OCT devices such as the Spectralis may be suitable in school-age children, but are not designed for young infants. Whilst high OCT imaging success rates were reported, many studies were limited to school

For peer review only - http://bmjopen.bmj.com/site/about/guidelines.xhtml

aged children rather than young infants. By contrast, this review found that the portable iVue device was successfully used for on-table OCTs in young infants under general anaesthesia,[22, 23] while the Envisu C2300 handheld OCT was used in conscious newborns without the need for general anaesthesia or pupil dilation in one feasibility study.[38]

Of note, handheld OCT has been recently used to describe the normal development of the optic nerve[27] and fovea[15, 16] in infants and children, with excellent feasibility. Handheld OCT has also been utilised in a wide range of other pedatric conditions including retinopathy of prematurity,[56] nystagmus[57], albinism[58], achromatopsia[59], foveal hypoplasia,[60] optic nerve hypoplasia,[61] primary congenital glaucoma[62], microcephaly[63] and others. Handheld OCT may be better tolerated in young children, particularly those with craniosynostosis associated with cognitive delay. However, further research is required to validate this. Therefore, our group has recently commenced prospective research using handheld OCT in craniosynostosis.

Strengths and limitations

This systematic review has a number of strengths. To the best of our knowledge, this is the first systematic review assessing the role of OCT in pediatric IH. Indeed, no such other systematic review appeared in our systematic search. PRISMA Guidelines[24] and rigorous Cochrane methods were followed.[25] Our protocol was registered on PROSPERO[26] and published[27] prior to this study, to promote transparency and avoid duplication. Two independent screeners (SRR and RJM) conducted the systematic search and two independent graders (SRR and MH) completed the quality assessment. A broad search strategy was developed with support from an experienced research librarian. Notably, our search terms did not restrict to certain conditions or age-related keywords – rather, the records were manually searched to avoid missing any key evidence. There were no time or language restrictions, yielding a broad range of eligible studies for inclusion including one article translated from Polish.[31]

We also acknowledge the limitations of this review. Meta-analysis was not possible due to the absence of eligible RCTs and inconsistency in OCT measures and gold standard measures of IH per study. The maximum grade of recommendations from this review was grade B, based on consistent level 2 or 3 studies or extrapolations from level 1 studies.[28] Notably, while some studies demonstrated OCT detection of papilloedema (e.g. RNFL thickening), other studies demonstrated OCT detection of optic atrophy (e.g. RNFL thinning), therefore caution must be exercised in interpreting OCT changes within the full clinical context and in serial examinations as far as possible. No level 1 evidence was returned by this review, which would be required to identify optimal OCT parameters and develop formal clinical guidelines. The broad search strategy resulted in a large number of records to screen, albeit this reduced the risk of missing key evidence.

Further research

This review highlighted the lack of standardisation in OCT parameters used to detect IH in children. Further research is required to clarify the most appropriate OCT parameters for this purpose, using gold standard ICP measures. This could be achieved by a validating prospective study using existing OCT reference standards. This should qualify as level 1b evidence as per the Oxford CEBM.[28] Handheld OCT could enable serial imaging in young infants without sedation, which would be particularly valuable as many pathologies associated with pediatric ICP can begin from birth, however current evidence is lacking. Serial OCT imaging could enable appreciation of evolving optic

For peer review only - http://bmjopen.bmj.com/site/about/guidelines.xhtml

nerve head and retinal changes over time, where applicable. Further high quality prospective studies could help to integrate OCT into formal clinical guidelines and clinical decision-making algorithms.

Other interesting research questions that were not answered in this systematic review include the following. First, how should OCT parameters be interpreted following IH where optic atrophy has occurred? The full clinical context, including visual function, may help indicate whether the patient has optic atrophy, rather than drawing conclusions based on OCT findings in isolation. Second, how should OCT parameters be interpreted in patients with gliosis of the optic nerve in chronic IH? This could prevent the typical ONH changes expected on OCT. Third, how should OCT be interpreted in recurrent IH which can occur in patients with ventriculoperitoneal or lumbar–peritoneal shunt obstruction? Again, gliosis may prevent the typical ONH changes expected on OCT. It is likely that OCT must be interpreted in the full clinical context, including visual function, to optimise diagnosis and management of these complex cases.

Conclusions

This systematic review has recognised the diagnostic potential of OCT in paediatric IH in craniosynostosis, IIH and SOL, in conjunction with established clinical measures of ICP, to guide diagnosis and management. However, this review could not recommend the development of formal guidelines, nor the widespread use of OCT in all children at risk of IH as standard clinical practice at this stage. Further validating prospective studies are required to improve our understanding of the clinical utility of OCT in this role (including handheld devices), to establish optimal OCT parameters for paediatric IH and to inform formal clinical guidelines.

For peer review only

Acknowledgements

We thank the authors of the included studies for providing further data where required for our systematic review. We thank Selina Lock, Research Services Consultant at the University of Leicester David Wilson Library, for providing expert guidance in our systematic search strategy. We thank Krystyna Ulanicka for providing the English translation of the included Polish study. SRR is funded by a National Institute for Health Research (NIHR) Doctoral Fellowship for this research project. This publication presents independent research funded by the MRC and National Institute for Health Research (NIHR). The views expressed are those of the author(s) and not necessarily those of the MRC, the NHS, the NIHR or the Department of Health and Social Care.

Authorship and contribution

Sohaib R. Rufai: Design and conceptualisation of the study; major role in the acquisition of data; analysis and interpretation of the data; drafted the manuscript for intellectual content.

Michael Hisaund: Analysis and interpretation of the data; revised the manuscript for intellectual content.

Noor ul Owase Jeelani: Design and conceptualisation of the study; revised the manuscript for intellectual content; supervision.

Rebecca J. McLean: Guarantor; design and conceptualisation of the study; major role in the acquisition of data; analysis and interpretation of the data; revised the manuscript for intellectual content; supervision.

Data sharing statement

No additional data available.

Ethical approval

No ethical approval was required for conducting this systematic review.

For peer review only

References

1. Quincke, H., Ueber Meningitis serosa und verwandte Zustände. *Deutsche Zeitschrift für Nervenheilkunde*. 1896. 9(3-4): p. 149-168.
2. Matthews YY, Dean F, Lim MJ, et al. Pseudotumor cerebri syndrome in childhood: incidence, clinical profile and risk factors in a national prospective population-based cohort study. *Arch Dis Child*. 2017;102(8):715-721.
3. Gillson N, Jones C, Reem RE, Rogers DL, Zumberge N, Aylward SC. Incidence and Demographics of Pediatric Intracranial Hypertension. *Pediatr Neurol*. 2017;73:42-47.
4. Derderian C, Seaward J. Syndromic craniosynostosis. *Semin Plast Surg*. 2012;26(2):64-75.
5. Jordan CO, Aylward SC. Intracranial hypertension: a current review. *Curr Opin Pediatr*. 2018;30(6):764-774.
6. Zhang X, Medow JE, Iskandar BJ, et al. Invasive and noninvasive means of measuring intracranial pressure: a review. *Physiol Meas*. 2017;38(8):R143-R182. Published 2017 Jul 24.
7. Tamburrini G, Caldarelli M, Massimi L, Santini P, Di Rocco C. Intracranial pressure monitoring in children with single suture and complex craniosynostosis: a review. *Childs Nerv Syst*. 2005;21(10):913-921.
8. Tuite GF, Chong WK, Evanson J, et al. The effectiveness of papilledema as an indicator of raised intracranial pressure in children with craniosynostosis. *Neurosurgery*. 1996;38(2):272-278.
9. Dubourg J, Javouhey E, Geeraerts T, Messerer M, Kassai B. Ultrasonography of optic nerve sheath diameter for detection of raised intracranial pressure: a systematic review and meta-analysis. *Intensive Care Med*. 2011;37(7):1059-1068.
10. Driessen C, Bannink N, Lequin M, et al. Are ultrasonography measurements of optic nerve sheath diameter an alternative to funduscopy in children with syndromic craniosynostosis?. *J Neurosurg Pediatr*. 2011;8(3):329-334.
11. Tuite GF, Evanson J, Chong WK, et al. The beaten copper cranium: a correlation between intracranial pressure, cranial radiographs, and computed tomographic scans in children with craniosynostosis. *Neurosurgery*. 1996;39(4):691-699.
12. Thompson DA, Liasis A, Hardy S, et al. Prevalence of abnormal pattern reversal visual evoked potentials in craniosynostosis. *Plast Reconstr Surg*. 2006;118(1):184-192.
13. Andersson L, Sjölund J, Nilsson J. Flash visual evoked potentials are unreliable as markers of ICP due to high variability in normal subjects. *Acta Neurochir (Wien)*. 2012;154(1):121-127.
14. Lee H, Proudlock FA, Gottlob I. Pediatric Optical Coherence Tomography in Clinical Practice-Recent Progress. *Invest Ophthalmol Vis Sci*. 2016;57(9):OCT69-OCT79.
15. Patel A, Purohit R, Lee H, et al. Optic Nerve Head Development in Healthy Infants and Children Using Handheld Spectral-Domain Optical Coherence Tomography. *Ophthalmology*. 2016;123(10):2147-2157.
16. Lee H, Purohit R, Patel A, et al. In Vivo Foveal Development Using Optical Coherence Tomography. *Invest Ophthalmol Vis Sci*. 2015;56(8):4537-4545.
17. Optical Coherence Tomography Substudy Committee; NORDIC Idiopathic Intracranial Hypertension Study Group. Papilledema Outcomes from the Optical Coherence Tomography Substudy of the Idiopathic Intracranial Hypertension Treatment Trial. *Ophthalmology*. 2015;122(9):1939-45.e2.

For peer review only - http://bmjopen.bmj.com/site/about/guidelines.xhtml

18. Scott CJ, Kardon RH, Lee AG, Frisén L, Wall M. Diagnosis and grading of papilledema in patients with raised intracranial pressure using optical coherence tomography vs clinical expert assessment using a clinical staging scale. *Arch Ophthalmol*. 2010;128(6):705-711.
19. Skau M, Yri H, Sander B, Gerds TA, Milea D, Jensen R. Diagnostic value of optical coherence tomography for intracranial pressure in idiopathic intracranial hypertension. *Graefes Arch Clin Exp Ophthalmol*. 2013;251(2):567-574.
20. Anand A, Pass A, Urfy MZ, et al. Optical coherence tomography of the optic nerve head detects acute changes in intracranial pressure. *J Clin Neurosci*. 2016;29:73-76.
21. Patel MD, Malhotra K, Shirazi Z, Moss HE. Methods for Quantifying Optic Disc Volume and Peripapillary Deflection Volume Using Radial Optical Coherence Tomography Scans and Association With Intracranial Pressure. *Front Neurol*. 2019;10:798.
22. Swanson JW, Aleman TS, Xu W, et al. Evaluation of Optical Coherence Tomography to Detect Elevated Intracranial Pressure in Children. *JAMA Ophthalmol*. 2017;135(4):320-328.
23. Swanson JW, Xu W, Ying GS, et al. Intracranial pressure patterns in children with craniosynostosis utilizing optical coherence tomography. *Childs Nerv Syst*. 2020;36(3):535-544.
24. Moher D, Liberati A, Tetzlaff J, Altman DG; PRISMA Group. Preferred reporting items for systematic reviews and meta-analyses: the PRISMA statement. *Ann Intern Med*. 2009;151(4):264-W64.
25. Cochrane Training. *Cochrane Handbook for Systematic Reviews of* Interventions. 2019. https://training.cochrane.org/handbook [Accessed 10 August 2020]
26. Sohaib Rufai. Detection of intracranial hypertension in children using optical coherence tomography: a systematic review. PROSPERO 2019 CRD42019154254 Available from: https://www.crd.york.ac.uk/prospero/display_record.php?ID=CRD42019154254 [Accessed 10 August 2020]
27. Rufai SR, Jeelani NUO, McLean RJ. Detection of intracranial hypertension in children using optical coherence tomography: a systematic review protocol. BMJ Open. 2020;10(7):e037833. Published 2020 Jul 6.
28. Oxford Centre for Evidence-based Medicine. *Levels of Evidence*. March 2009. https://www.cebm.net/2009/06/oxford-centre-evidence-based-medicine-levels-evidence-march-2009/ [Accessed 10 August 2020]
29. Cochrane. *Data extraction forms.* 2019. https://dplp.cochrane.org/data-extraction-forms [Accessed 10 August 2020]
30. NIH. *Study Quality Assessment Tools: Quality Assessment Tool for Observational Cohort and Cross-Sectional Studies*. https://www.nhlbi.nih.gov/health-topics/study-quality-assessment-tools [Accessed 10 August 2020]
31. Mrugacz M, Szumiński M, Bakunowicz-Łazarczyk A. Optyczna koherentna tomografia w diagnostyce nadciśnienia wewnatrzczaszkowego u dzieci i młodziezy [Optical coherence tomography in diagnosis of intracranial hypertension in children]. *Klin Oczna*. 2011;113(7-9):258-262.
32. Chang MY, Velez FG, Demer JL, et al. Accuracy of Diagnostic Imaging Modalities for Classifying Pediatric Eyes as Papilledema Versus Pseudopapilledema. *Ophthalmology*. 2017;124(12):1839-1848.

For peer review only - http://bmjopen.bmj.com/site/about/guidelines.xhtml

33. den Ottelander BK, de Goederen R, van Veelen MC, et al. Muenke syndrome: long-term outcome of a syndrome-specific treatment protocol [published online ahead of print, 2019 Jul 19]. *J Neurosurg Pediatr*. 2019;1-8.
34. Driessen C, Eveleens J, Bleyen I, van Veelen ML, Joosten K, Mathijssen I. Optical coherence tomography: a quantitative tool to screen for papilledema in craniosynostosis. *Childs Nerv Syst*. 2014;30(6):1067-1073.
35. El-Dairi MA, Holgado S, O'Donnell T, Buckley EG, Asrani S, Freedman SF. Optical coherence tomography as a tool for monitoring pediatric pseudotumor cerebri. *J AAPOS*. 2007;11(6):564-570.
36. Lee YA, Tomsak RL, Sadikovic Z, Bahl R, Sivaswamy L. Use of Ocular Coherence Tomography in Children With Idiopathic Intracranial Hypertension-A Single-Center Experience. *Pediatr Neurol*. 2016;58:101-106.e1.
37. Lee WJ, Kim HJ, Park KH, et al. Change in Optic Nerve After Intracranial Pressure Reduction in Children. *Ophthalmology*. 2017;124(11):1713-1715.
38. Tran-Viet D, Wong BM, Mangalesh S, Maldonado R, Cotten CM, Toth CA. HANDHELD SPECTRAL DOMAIN OPTICAL COHERENCE TOMOGRAPHY IMAGING THROUGH THE UNDILATED PUPIL IN INFANTS BORN PRETERM OR WITH HYPOXIC INJURY OR HYDROCEPHALUS. *Retina*. 2018;38(8):1588-1594.
39. Bialer OY, Goldenberg-Cohen N, Toledano H, Snir M, Michowiz S. Retinal NFL thinning on OCT correlates with visual field loss in pediatric craniopharyngioma. *Can J Ophthalmol*. 2013;48(6):494-499.
40. Dagi LR, Tiedemann LM, Heidary G, Robson CD, Hall AM, Zurakowski D. Using spectral-domain optical coherence tomography to detect optic neuropathy in patients with craniosynostosis. *J AAPOS*. 2014;18(6):543-549.
41. Dahlmann-Noor AH, Adams GW, Daniel MC, et al. Detecting optic nerve head swelling on ultrasound and optical coherence tomography in children and young people: an observational study. *Br J Ophthalmol*. 2018;102(3):318-322.
42. Gospe SM 3rd, Bhatti MT, El-Dairi MA. Anatomic and visual function outcomes in paediatric idiopathic intracranial hypertension. *Br J Ophthalmol*. 2016;100(4):505-509.
43. Malem A, De Salvo G, West S. Use of MultiColor imaging in the assessment of suspected papilledema in 20 consecutive children. *J AAPOS*. 2016;20(6):532-536.
44. Mediero S, Noval S, Bravo-Ljubetic L, Contreras I, Carceller F. Visual Outcomes, Visual Fields, and Optical Coherence Tomography in Paediatric Craniopharyngioma. *Neuroophthalmology*. 2015;39(3):132-139.
45. Krishnakumar D, Pickard JD, Czosnyka Z, Allen L, Parker A. Idiopathic intracranial hypertension in childhood: pitfalls in diagnosis. *Dev Med Child Neurol*. 2014;56(8):749-755.
46. Ozturk Z, Atalay T, Arhan E, et al. The efficacy of orbital ultrasonography and magnetic resonance imaging findings with direct measurement of intracranial pressure in distinguishing papilledema from pseudopapilledema. *Childs Nerv Syst*. 2017;33(9):1501-1507.
47. Sánchez-Tocino H, Bringas R, Iglesias D, González-Pérez A, Del Villar-Galán R. Utilidad del tomógrafo de coherencia óptica (OCT) en el seguimiento de la hipertensión intracraneal idiopática en la infancia [Utility of optic coherence tomography (OCT) in the follow-up of idiopathic intracranial hypertension in childhood]. *Arch Soc Esp Oftalmol*. 2006;81(7):383-389.

For peer review only - http://bmjopen.bmj.com/site/about/guidelines.xhtml

48. Thompson AC, Bhatti MT, El-Dairi MA. Bruch's membrane opening on optical coherence tomography in pediatric papilledema and pseudopapilledema. *J AAPOS*. 2018;22(1):38-43.e3.
49. van de Beeten SDC, Cornelissen MJ, van Seeters RM, et al. Papilledema in unicoronal synostosis: a rare finding [published online ahead of print, 2019 May 17]. *J Neurosurg Pediatr*. 2019;1-6.
50. Hayward R, Britto J, Dunaway D, Jeelani O. Connecting raised intracranial pressure and cognitive delay in craniosynostosis: many assumptions, little evidence. *J Neurosurg Pediatr*. 2016;18(2):242-250.
51. Dandy WE. INTRACRANIAL PRESSURE WITHOUT BRAIN TUMOR: DIAGNOSIS AND TREATMENT. *Ann Surg*. 1937;106(4):492-513.
52. Friedman DI, Liu GT, Digre KB. Revised diagnostic criteria for the pseudotumor cerebri syndrome in adults and children. *Neurology*. 2013;81(13):1159-1165.
53. Mollan SP, Davies B, Silver NC, et al. Idiopathic intracranial hypertension: consensus guidelines on management. *J Neurol Neurosurg Psychiatry*. 2018;89(10):1088-1100.
54. Mathijssen IM. Guideline for Care of Patients With the Diagnoses of Craniosynostosis: Working Group on Craniosynostosis. *J Craniofac Surg*. 2015;26(6):1735-1807.
55. Optical Coherence Tomography Substudy Committee; NORDIC Idiopathic Intracranial Hypertension Study Group. Papilledema Outcomes from the Optical Coherence Tomography Substudy of the Idiopathic Intracranial Hypertension Treatment Trial. *Ophthalmology*. 2015;122(9):1939-45.e2.
56. Anwar S, Nath M, Patel A, et al. POTENTIAL UTILITY OF FOVEAL MORPHOLOGY IN PRETERM INFANTS MEASURED USING HAND-HELD OPTICAL COHERENCE TOMOGRAPHY IN RETINOPATHY OF PREMATURITY SCREENING. *Retina*. 2020;40(8):1592-1602.
57. Lee H, Proudlock F, Gottlob I. Is handheld optical coherence tomography reliable in infants and young children with and without nystagmus?. *Invest Ophthalmol Vis Sci*. 2013;54(13):8152-8159.
58. Mohammad S, Gottlob I, Sheth V, et al. Characterization of Abnormal Optic Nerve Head Morphology in Albinism Using Optical Coherence Tomography. *Invest Ophthalmol Vis Sci*. 2015;56(8):4611-4618.
59. Thomas MG, Kumar A, Kohl S, Proudlock FA, Gottlob I. High-resolution in vivo imaging in achromatopsia. *Ophthalmology*. 2011;118(5):882-887.
60. Rufai SR, Thomas MG, Purohit R, et al. Can Structural Grading of Foveal Hypoplasia Predict Future Vision in Infantile Nystagmus?: A Longitudinal Study. *Ophthalmology*. 2020;127(4):492-500.
61. Pilat A, Sibley D, McLean RJ, Proudlock FA, Gottlob I. High-Resolution Imaging of the Optic Nerve and Retina in Optic Nerve Hypoplasia. *Ophthalmology*. 2015;122(7):1330-1339.
62. Pilat AV, Proudlock FA, Shah S, et al. Assessment of the anterior segment of patients with primary congenital glaucoma using handheld optical coherence tomography. *Eye (Lond)*. 2019;33(8):1232-1239.
63. Papageorgiou E, Pilat A, Proudlock F, et al. Retinal and optic nerve changes in microcephaly: An optical coherence tomography study. *Neurology*. 2018;91(6):e571-e585.

For peer review only - http://bmjopen.bmj.com/site/about/guidelines.xhtml

Figure Legends

Figure 1: PRISMA study inclusion flow diagram. Key: *Reasons for exclusion were as follows: adult studies (n=98); mixed studies of adults and children without breakdown (n=27); conference abstracts (n=86); case reports (n=6); correspondence (n=4). Supplemental Table 1 contains the full list of excluded studies with reasons.

Figure 2. Optical Coherence Tomographic (OCT) Parameters, Variation With Intracranial Pressure (ICP), and Utility as a Screening Test. (A) 1. RNFL thickness; 2. retinal thickness; the asterisk indicates cascular elements causing posterior shadowing; (B) 3. Anterior retinal projection, where the dotted white line is a vector connecting the posterior-most ILM adjacent to either side of the optic disc. (C) OCT images of patients with normal (Patient 1; ICP, 6 mm Hg) and elevated (Patient 2; ICP, 18 mm Hg and Patient 3; ICP, 20 mm Hg) ICP. (D) OCT retinal parameters (maximal retinal thickness, anterior projection, and maximal RNFL thickness) plotted as a function of ICP measured intraoperatively. (E) ROC curves for each of the 3 OCT parameters, combined RNFL and maximal retinal thickness parameters, and a model combining all parameters. GCL = ganglion cell layer; ILM = inner limiting membrane; INL = inner nuclear layer; ONL = outer nuclear layer; RNFL = retinal nerve fibre layer; ROC = receiver operating characteristic; RPE = retinal pigment epithelium.

Reprinted with permission from: Swanson JW, Aleman TS, Xu W, et al. Evaluation of Optical Coherence Tomography to Detect Elevated Intracranial Pressure in Children. *JAMA Ophthalmol*. 2017;135(4):320-328, Copyright © 2018 American Medical Association

Figure 3. Measuring the transverse horizontal diameter of Bruch's membrane opening (BMO) and the papillary height on SD-OCT. (A) when the ONH is swollen from mild papilledema; (B) when the papilledema has resolved.

Reprinted with permission from: Thompson AC, Bhatti MT, El-Dairi MA. Bruch's membrane opening on optical coherence tomography in pediatric papilledema and pseudopapilledema. *J AAPOS*. 2018;22(1):38-43, Copyright © 2018 Elsevier.

Figure 4: Swept-source optical coherence tomography (SS-OCT) images (horizontal scan) of optic nerve head structures in the right eye of an 8-year-old boy with pilocytic astrocytoma. Disc photographs showing preoperative papilledema (A) and postoperative resolution of the same (B) are presented with the indicating OCT section (small squares). A row SS-OCT image of preoperative (C) and postoperative (D) states can be observed. Measurements are obtained using the preoperative (E) and postoperative (F) images. After surgical decompression, the neural canal diameter (red line) and papillary vertical height (green line) have decreased and the lamina cribrosa (LC) shows posterior displacement (yellow dotted line; anterior surface of the LC contour). IICP = increased intracranial pressure.

Reprinted with permission from: Lee WJ, Kim HJ, Park KH, et al. Change in Optic Nerve After Intracranial Pressure Reduction in Children. *Ophthalmology*. 2017;124(11):1713-1715, Copyright © 2017 Elsevier.

For peer review only - http://bmjopen.bmj.com/site/about/guidelines.xhtml

For peer review only

For peer review only - http://bmjopen.bmj.com/site/about/guidelines.xhtml

Source	Country	Study design	Conditions	Total number of study participants	Age	Level of Evidence (1-5)*	Quality rating†
Prospective studies							
**Chang 2017[32]**	US	Prospective observational study	Other	19	M: 11; Ra: 5-17	2b	⊕⊕⊖
**Den Ottelander 2019[33]**	The Netherlands	Prospective cohort study	Craniosynostosis	38	M: 0.7; Ra: 0.2-1.5	2b	⊕⊖⊖
**Driessen 2014[34]**	The Netherlands	Prospective cohort study	Craniosynostosis	38	M: 6.2; Ra: 3.0-11.0	2b	⊕⊖⊖
**El-Dairi 2007[35]**	US	Prospective observational study	IIH	48	Mdn: 11; Ra: 4-14‡	2b	⊕⊕⊖
**Lee 2016[36]**	US	Prospective observational study	IIH	13	M: 14.8; SD 3.1‡	2b	⊕⊕⊖
**Lee 2017[37]**	South Korea	Prospective observational study	SOL, other	10	M: 12.7; SD: 2.7	2b	⊕⊕⊖
**Tran-Viet 2018[38]**	US	Prospective feasibility study	Other	11	Ra: 0.7-2	3b	⊕⊖⊖
**Swanson 2017[22]**	US	Prospective observational study	Craniosynostosis, other	79	M: 2.9; SD: 3.8; Ra: 0.3-15.0‡	2b	⊕⊕⊖
**Swanson 2020[23]**	US	Prospective observational study	Craniosynostosis	80	M: 3.4; SD: 4.7; Ra: 0.2-18	2b	⊕⊖⊖
Retrospective studies							
**Bialer 2013[39]**	Israel	Retrospective chart review	SOL	20	M: 6.5; SD: 3.9	2b	⊕⊖⊖
**Dagi 2014[40]**	US	Retrospective chart review	Craniosynostosis	54	Mdn: 9.3; SD: 4.6	2b	⊕⊖⊖
**Dahlman-Noor 2018[41]**	UK	Retrospective cohort study	SOL, other	61	Mdn: 10.9; IQR: 7.9-13	2b	⊕⊕⊖
**Gospe 2016[42]**	US	Retrospective chart review	IIH	31	7.8; SD: 3.4	2b	⊕⊕⊖
**Malem 2016[43]**	UK	Case series	SOL, IIH, other	20	M: 11; Ra: 5-16	4	⊕⊕⊖
**Mediero 2015[44]**	Spain	Case series	SOL	10	Mdn: 5; Ra: 3-16	4	⊕⊖⊖
**Mrugacz 2011[31]**	Poland§	Case series	IIH, other	4	Mdn: 9.5; Ra: 3-12	4	⊕⊖⊖
**Krishnakumar 2014[45]**	UK	Retrospective chart review	IIH	16	Mdn: 12; Ra: 3-15	3b	⊕⊕⊖
**Ozturk 2017[46]**	Turkey	Retrospective observational study	IIH	16	M: 11.4; SD: 4.1; Ra: 3-17	3b	⊕⊖⊖
**Sanchez-Tocino 2006[47]**	Spain	Case series	IIH	3	M: 8.3; SD: 3.8; Ra: 4-11	4	⊕⊖⊖
**Thompson 2018[48]**	US	Retrospective cohort study	IIH	90	M: 12.2; SD: 3.7‡	2b	⊕⊕⊖
**Van de Beeten 2019[49]**	The Netherlands	Retrospective chart review	Craniosynostosis	104	M: 0.9; Ra: 0.5-1.7	3b	⊕⊖⊖

Table 1: Study characteristics and quality ratings of included studies. Key: * Levels of evidence as per the Oxford Centre for Evidence-Based Medicine; † The National Institutes of Health (NIH) Quality Assessment Tool was used: ⊕⊕⊕, good; ⊕⊕⊖, fair; ⊕⊖⊖, poor; ‡ Where separate age ranges were reported for patients and controls, the age range for patients is displayed here; § Article written in Polish. IIH = idiopathic intracranial hypertension; M = mean; Mdn = median; Ra = range; SD = standard deviation

For peer review only - http://bmjopen.bmj.com/site/about/guidelines.xhtml

Source	OCT Parameter(s)	Gold standard	Diagnostic capability of OCT
Prospective studies			
**Chang 2017[32]**	i) RNFL thickness; ii) volumetric SD-OCT of ONH; iii) volumetric EDI-OCT of ONH	Clinical diagnosis	i) 69% accuracy; ii) 71% accuracy; iii) 67% accuracy
**Den Ottelander 2019[33]**	TRT	Fundoscopy and/or 24 hour ICP	No patients had abnormal TRT
**Driessen 2014[34]**	TRT	Fundoscopy	TRT increased in abnormal fundoscopy (TRT p<0.001)
**El-Dairi 2007[35]**	i) RNFL thickness; ii) macular volume	Modified Dandy IIH criteria	i) Thicker RNFL in IIH vs controls (p<0.0001); ii) thicker macular volume in IIH vs controls (p=0.0008)
**Lee 2016[36]**	RNFL thickness	Fundoscopy	RE: r=0.633, p=0.02; LE: r=0.868, P=0.001
**Lee 2017[37]**	i) Neural canal diameter; ii) papillary vertical height; iii) anterior LC depth	Single ICP measures	i) Post-op reduction (p=0.027); ii) post-op reduction (p<0.001); iii) post-op increase (p=0.001)
**Tran-Viet 2018[38]**	Not specified	Clinical diagnosis	Not assessed
**Swanson 2017[22]**	i) Maximal RNFL thickness; ii) maximal retinal thickness iii) maximal anterior retinal projection; iv) Combination of i) and iii)	Single ICP measures	i) Sens: 79%; Spec: 81%; ii) Sens: 63%; Spec: 86%; iii) Sens: 84%; Spec: 67%; iv) Sens: 89%; Spec: 62%
**Swanson 2020[23]**	Combination of RNFL thickness, maximal retinal thickness and maximal anterior retinal projection	OCT	Not assessed
Retrospective studies			
**Bialer 2013[39]**	RNFL thickness	Clinical diagnosis	Significantly lower in optic atrophy vs. controls (p<0.001)
**Dagi 2014[40]**	RNFL thickness	Fundoscopy	Optic atrophy: Sens: 88%; spec: 94%; papilledema: Sens: 60%; spec: 90%; all: Sens: 77%; spec: 83%
**Dahlman-Noor 2018[41]**	i) RNFL thickness; ii) anterior bowing of BM	Clinical diagnosis	i) Present in 2 in 3 children; ii) present in 1 in 3 children.
**Gospe 2016[42]**	i) Optic atrophy: RNFL thickness <80µm; ii) disruption of the ellipsoid zone (photoreceptor loss)	Revised Friedman IIH criteria	i) OR for any vision loss: infinite (p<0.0001); ii) OR for any vision loss: 120 (p<0.0001)
**Malem 2016[43]**	RNFL thickness	Neuroimaging +/- LP	Not assessed
**Mediero 2015[44]**	i) GCL thickness; ii) RNFL thickness	HVF	i) kappa = 1; p < 0.001; ii) kappa = 0.632; p = 0.011
**Mrugacz 2011[31]**	RNFL thickness	Clinical diagnosis	Not assessed
**Krishnakumar 2014[45]**	Not specified	LP	Not assessed
**Ozturk 2017[46]**	Not specified	LP	Not assessed
**Sanchez-Tocino 2006[47]**	RNFL thickness	LP	Not assessed
**Thompson 2018[48]**	i) BMO; ii) RNFL	Revised Friedman IIH criteria	i) AUC=0.81; ii) AUC=0.96. Combined cut-offs: Sens: 91.7%; Spec: 92.2%
**Van de Beeten 2019[49]**	TRT	Clinical diagnosis	Not assessed

Table 2: Main outcome measures. Key: BM = Bruch's membrane; EDI = enhanced depth imaging; GCL = ganglion cell layer; HVF = Humphrey visual fields; LC = lamina cribrosa; LE = left eye; LP = lumbar puncture; OR = odds ratio; RE = right eye; RNFL = retinal nerve fibre layer; SD = spectral domain; TRT = total retinal thickness; op = operation

For peer review only - http://bmjopen.bmj.com/site/about/guidelines.xhtml

This page has been left blank intentionally

For peer review only

For peer review only - http://bmjopen.bmj.com/site/about/guidelines.xhtml

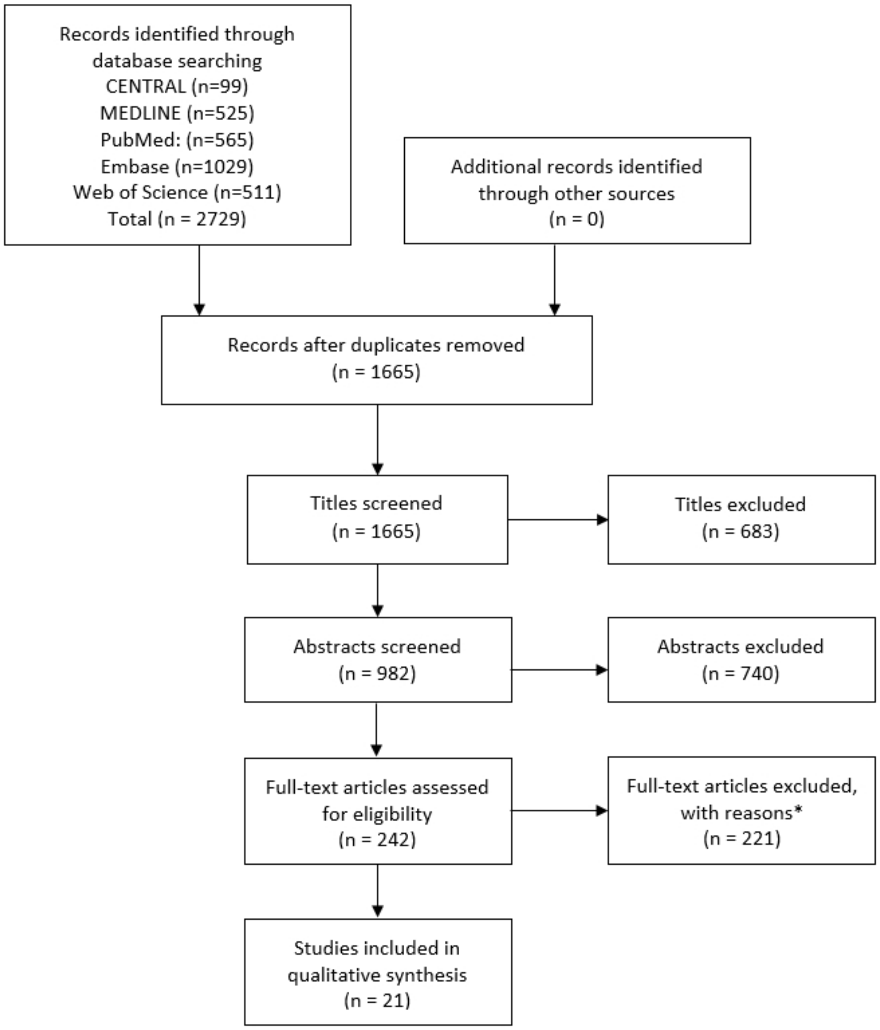

197x218mm (96 x 96 DPI)

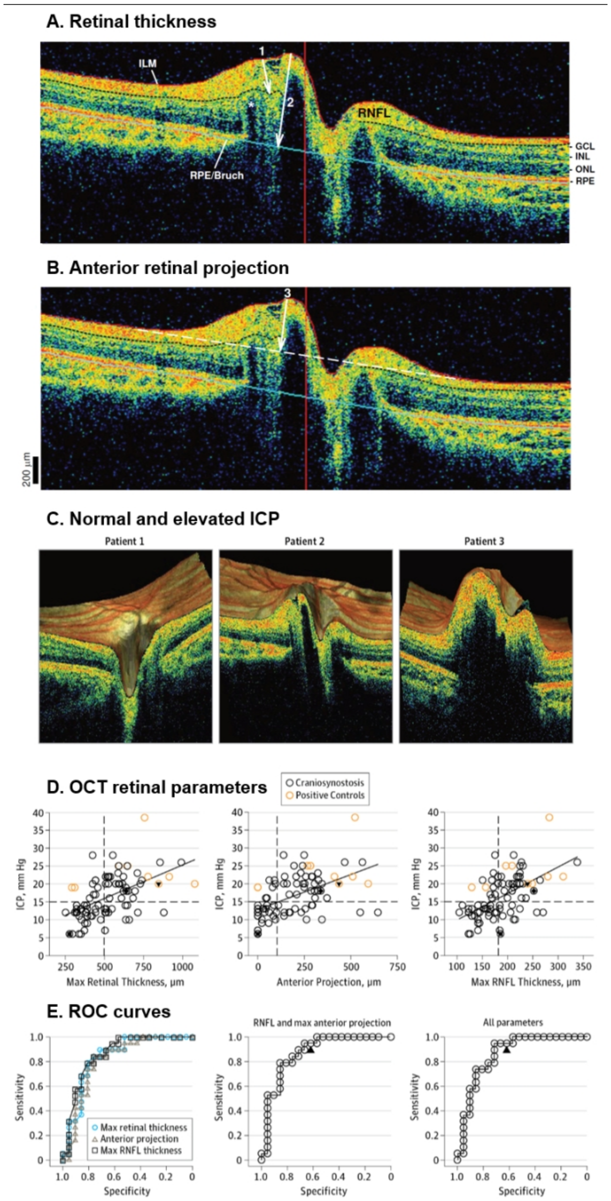

130x258mm (150 x 150 DPI)

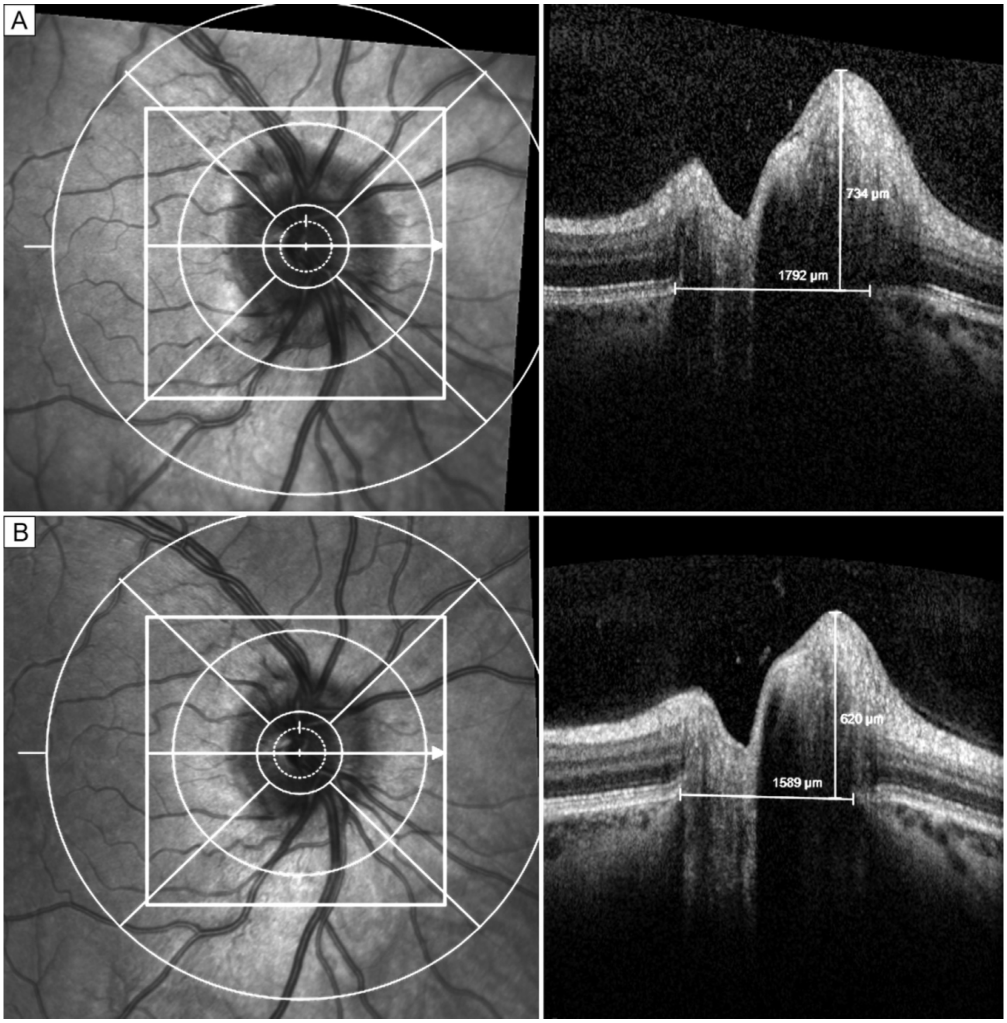

263x267mm (144 x 144 DPI)

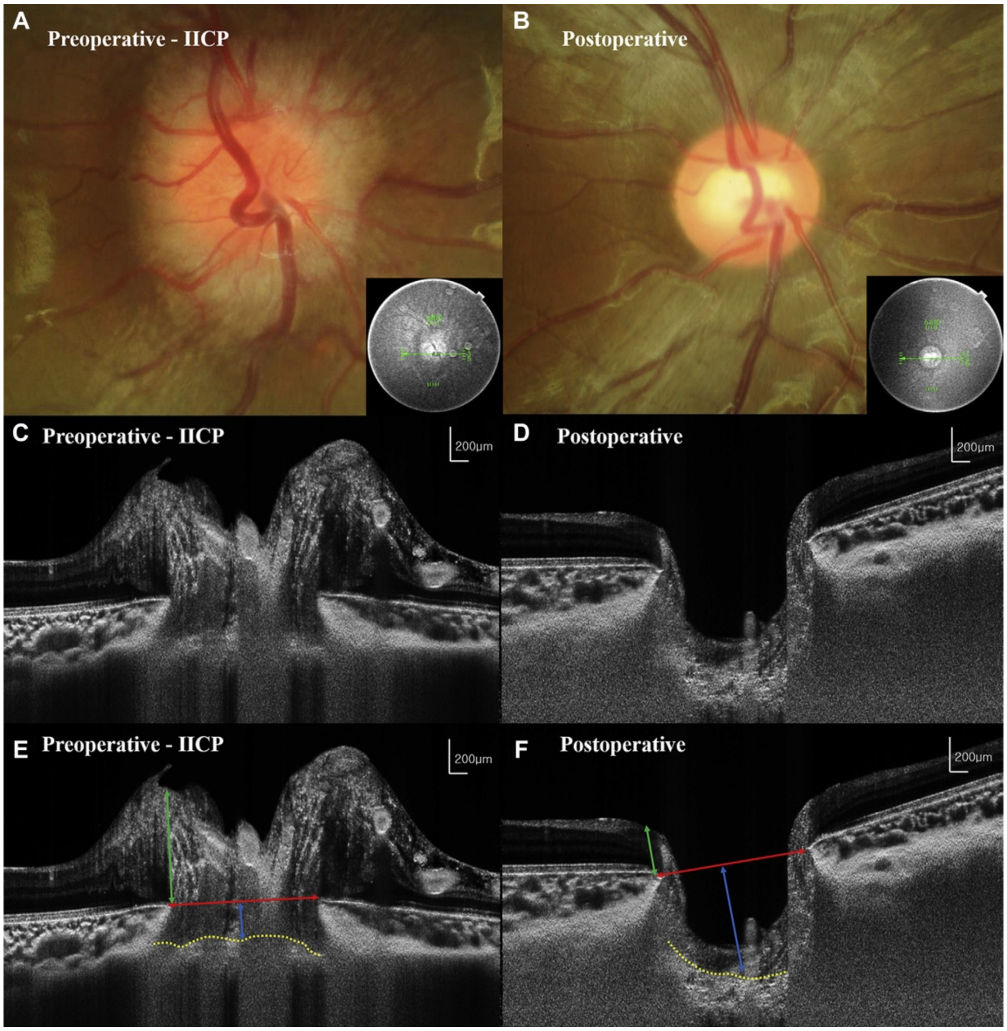

364x372mm (144 x 144 DPI)

Supplemental Data

Contents

Appendix 1: Search terms....2

Appendix 2: Screening questions....3

Appendix 3: Data extraction tool, adapted from the Cochrane Collaboration....4

Appendix 4: NIH Quality Assessment Tool for Observational Cohort and Cross-Sectional Studies ...7

Supplemental Table 1: Excluded studies with reasons....8

For peer review only

For peer review only - http://bmjopen.bmj.com/site/about/guidelines.xhtml

Appendix 1: Search terms

Intracranial pressure, intracranial hypertension, papilloedema, optical coherence tomography

1. Intracranial pressure
2. ICP
3. Intracerebral pressure
4. Intracranial hypertension
5. Cerebral swelling
6. Cerebral oedema
7. Cerebral edema
8. Brain swelling
9. Brain oedema
10. Brain edema
11. Papilloedema
12. Papilledema
13. Optical coherence tomography
14. OCT

Search combination:

1 OR 2 OR 3 OR 4 OR 5 OR 6 OR 7 OR 8 OR 9 OR 10 OR 11 OR 12

AND

13 or 14

For peer review only

For peer review only - http://bmjopen.bmj.com/site/about/guidelines.xhtml

Appendix 2: Screening questions

Instructions for screeners: Tick the appropriate box per screening question. If "yes" at Stage 1, proceed to Stage 2; if "yes" at Stage 2, include. If "no" at any stage, exclude. If "unclear", proceed to next stage. If still "unclear" after Stage 2, then submit to third arbitrator for verdict.

Stage 1: Title Screening

Does the study represent Level 4 evidence or above, i.e. case series, case-control studies, cohort studies, randomised controlled trials (RCTs) and systematic reviews?

Yes	
No	
Unclear	

Does the study pertain to the use of optical coherence tomography (OCT) to detect raised intracranial pressure (ICP) in children, defined as 0 to 18 years of age?

Yes	
No	
Unclear	

Stage 2: Abstract screening

Does the study represent Level IV evidence or above, i.e. case series, cohort studies, case-control studies, randomised controlled trials (RCTs) and systematic reviews?

Yes	
No	
Unclear	

Does the study pertain to the use of optical coherence tomography (OCT) to detect raised intracranial pressure (ICP) in children, defined as 0 to 18 years of age?

Yes	
No	
Unclear	

Stage 3: Full paper screening

Does the study pertain to the use of optical coherence tomography (OCT) to detect raised intracranial pressure (ICP) in children, defined as 0 to 18 years of age?

Yes	
No	
Unclear	

For peer review only - http://bmjopen.bmj.com/site/about/guidelines.xhtml

For peer review only

Appendix 3: Data extraction tool, adapted from the Cochrane Collaboration

General Information

Study title:
Author(s):
Source:
Date of Extraction:

Methods

Aim of study	
Study design	
Inclusion criteria	
Exclusion criteria	
Method of recruitment	
Method of randomisation (if applicable)	
Blinding (if applicable)	
Study duration	
Ethics approval	

Participants

Population description	
Setting	
Total number randomised	
Mean age	
Male/Female %	

Intervention 1

Group name	
Number in group	
Description	
Intervention parameter 1	
Intervention parameter 2 (if applicable)	
Intervention parameter 3 (if applicable)	
Intervention parameter 4 (if applicable)	
Intervention parameter 5 (if applicable)	
Intervention parameter 6 (if applicable)	
Intervention parameter 7 (if applicable)	
Intervention parameter 8 (if applicable)	
Drop-outs from group	

Outcome 1

Outcome group name	
Time points measured	

For peer review only - http://bmjopen.bmj.com/site/about/guidelines.xhtml

Outcome definition	
Outcome parameter 1	
Outcome parameter 2 (if applicable)	
Outcome parameter 3 (if applicable)	
Outcome parameter 4 (if applicable)	
Outcome parameter 5 (if applicable)	
Outcome parameter 6 (if applicable)	
Outcome parameter 7 (if applicable)	
Outcome parameter 8 (if applicable)	
Person measuring	
Imputation of missing data	

Intervention 2 (if applicable)

Intervention group name	
Number in group	
Description	
Intervention parameter 1	
Intervention parameter 2 (if applicable)	
Intervention parameter 3 (if applicable)	
Intervention parameter 4 (if applicable)	
Intervention parameter 5 (if applicable)	
Intervention parameter 6 (if applicable)	
Intervention parameter 7 (if applicable)	
Intervention parameter 8 (if applicable)	
Drop-outs from group	

Outcome 2 (if applicable)

Outcome group name	
Time points measured	
Outcome definition	
Outcome parameter 1	
Outcome parameter 2 (if applicable)	
Outcome parameter 3 (if applicable)	
Outcome parameter 4 (if applicable)	
Outcome parameter 5 (if applicable)	
Outcome parameter 6 (if applicable)	
Outcome parameter 7 (if applicable)	
Outcome parameter 8 (if applicable)	
Person measuring	
Imputation of missing data	

For peer review only - http://bmjopen.bmj.com/site/about/guidelines.xhtml

Intervention 3 (if applicable)

Intervention group name	
Number in group	
Description	
Intervention parameter 1	
Intervention parameter 2 (if applicable)	
Intervention parameter 3 (if applicable)	
Intervention parameter 4 (if applicable)	
Intervention parameter 5 (if applicable)	
Intervention parameter 6 (if applicable)	
Intervention parameter 7 (if applicable)	
Intervention parameter 8 (if applicable)	
Drop-outs from group	

Outcome 3 (if applicable)

Outcome group name	
Time points measured	
Outcome definition	
Outcome parameter 1	
Outcome parameter 2 (if applicable)	
Outcome parameter 3 (if applicable)	
Outcome parameter 4 (if applicable)	
Outcome parameter 5 (if applicable)	
Outcome parameter 6 (if applicable)	
Outcome parameter 7 (if applicable)	
Outcome parameter 8 (if applicable)	
Person measuring	
Imputation of missing data	

For peer review only

For peer review only - http://bmjopen.bmj.com/site/about/guidelines.xhtml

Appendix 4: NIH Quality Assessment Tool for Observational Cohort and Cross-Sectional Studies

**Criteria**	**Yes**	**No**	**Other (CD, NR, NA)***
1. Was the research question or objective in this paper clearly stated?			
2. Was the study population clearly specified and defined?			
3. Was the participation rate of eligible persons at least 50%?			
4. Were all the subjects selected or recruited from the same or similar populations (including the same time period)? Were inclusion and exclusion criteria for being in the study prespecified and applied uniformly to all participants?			
5. Was a sample size justification, power description, or variance and effect estimates provided?			
6. For the analyses in this paper, were the exposure(s) of interest measured prior to the outcome(s) being measured?			
7. Was the timeframe sufficient so that one could reasonably expect to see an association between exposure and outcome if it existed?			
8. For exposures that can vary in amount or level, did the study examine different levels of the exposure as related to the outcome (e.g., categories of exposure, or exposure measured as continuous variable)?			
9. Were the exposure measures (independent variables) clearly defined, valid, reliable, and implemented consistently across all study participants?			
10. Was the exposure(s) assessed more than once over time?			
11. Were the outcome measures (dependent variables) clearly defined, valid, reliable, and implemented consistently across all study participants?			
12. Were the outcome assessors blinded to the exposure status of participants?			
13. Was loss to follow-up after baseline 20% or less?			
14. Were key potential confounding variables measured and adjusted statistically for their impact on the relationship between exposure(s) and outcome(s)?			

*CD, cannot determine; NA, not applicable; NR, not reported

**Quality Rating (Good, Fair, or Poor)**	
**Rater #1 initials:**	
**Rater #2 initials:**	
**Additional Comments (If POOR, please state why):**	

For peer review only

Supplemental Table 1: Excluded studies with reasons

Key: *Reasons for exclusion were as follows: A: adult studies (n=98); B: mixed adults and children without breakdown (n=27); C: conference abstracts (n=86); D: case reports (n=6); E: correspondence (n=4).

Number	Reference	Reason for exclusion*
1	Afonso, C.L., et al., Relationship between pattern electroretinogram, frequency-domain OCT, and automated perimetry in chronic papilledema from pseudotumor cerebri syndrome. Investigative Ophthalmology and Visual Science, 2015. 56(6): p. 3656-3665.	A
2	Afonso, J.M., et al., Spectral Domain-Optical Coherence Tomography As a New Diagnostic Marker for Idiopathic Normal Pressure Hydrocephalus. Frontiers in Neurology, 2017. 8: p. 10.	A
3	Aghsaei Fard, M., et al., Optic Nerve Head and Macular Optical Coherence Tomography Measurements in Papilledema Compared With Pseudopapilledema. Journal of neuro-ophthalmology : the official journal of the North American Neuro-Ophthalmology Society, 2019. 39(1): p. 28-34.	A
4	Albrecht, P., et al., Optical coherence tomography for the diagnosis and monitoring of idiopathic intracranial hypertension. Journal of neurology, 2017. 264(7): p. 1370-1380.	A
5	Alessi, G., et al., Evolution en tomographie par coherence optique de l'oedme papillaire dans l'hypertension intracranienne idiopathique traitee par mise en place de stent endosinusien du sinus lateralOptical coherence tomography in following up papilledema in idiopathic intracranial hypertension treated with lateral sinus stent placement. Journal Francais d'Ophtalmologie, 2010. 33(9): p. 637-648.	A
6	Anand, A., et al., Optical coherence tomography of the optic nerve head detects acute changes in intracranial pressure. Journal of Clinical Neuroscience, 2016. 29: p. 73-76.	A
7	Aojula, A., et al., Segmentation error in spectral domain optical coherence tomography measures of the retinal nerve fibre layer thickness in idiopathic intracranial hypertension. BMC ophthalmology, 2018. 17(1): p. 257.	A
8	Asian, F. and B. Ozkal, Optical coherence tomography measurements of the optic nerve head and retina in newly diagnosed idiopathic intracranial hypertension without loss of vision. Ceska a Slovenska Neurologie a Neurochirurgie, 2019. 82(3): p. 309-315.	A
9	Athappilly, G., et al., Ganglion Cell Complex Analysis as a Potential Indicator of Early Neuronal Loss in Idiopathic Intracranial Hypertension. Neuro-ophthalmology (Aeolus Press), 2019. 43(1): p. 10-17.	A
10	Auinger, P., et al., Baseline OCT Measurements in the Idiopathic Intracranial Hypertension Treatment Trial, Part II: Correlations and Relationship to Clinical Features. Investigative Ophthalmology & Visual Science, 2014. 55(12): p. 8173-8179.	A

For peer review only - http://bmjopen.bmj.com/site/about/guidelines.xhtml

11	Auinger, P., et al., Papilledema Outcomes from the Optical Coherence Tomography Substudy of the Idiopathic Intracranial Hypertension Treatment Trial. Ophthalmology, 2015. 122(9): p. 1939-1945.	A
12	Bassi, S.T. and K.P. Mohana, Optical coherence tomography in papilledema and pseudopapilledema with and without optic nerve head drusen. Indian journal of ophthalmology, 2014. 62(12): p. 1146-51.	A
13	Bilen, F.T. and H. Atilla, Peripapillary Vessel Density Measured by Optical Coherence Tomography Angiography in Idiopathic Intracranial Hypertension. Journal of Neuro-Ophthalmology, 2019. 39(3): p. 319-323.	A
14	Carey AR, Bosley TM, Miller NR, McCulley TJ, Henderson AD. Use of En Face Optical Coherence Tomography to Monitor Papilledema in Idiopathic Intracranial Hypertension: A Pilot Study [published online ahead of print, 2020 Mar 24]. J Neuroophthalmol. 2020;10.1097/WNO.0000000000000940. doi:10.1097/WNO.0000000000000940	A
15	Carta, A., et al., Optical coherence tomography is a useful tool in the differentiation between true edema and pseudoedema of the optic disc. PLoS ONE, 2018. 13(11).	A
16	Chen, J.J., et al., Optic disc edema in glial fibrillary acidic protein autoantibody-positive meningoencephalitis. Journal of Neuro-Ophthalmology, 2018. 38(3): p. 276-281.	A
17	Choi, S.S., et al., Changes in cellular structures revealed by ultra-high resolution retinal imaging in optic neuropathies. Investigative ophthalmology & visual science, 2008. 49(5): p. 2103-19.	A
18	Dreesbach, M., et al., Optic nerve head volumetry by optical coherence tomography in papilledema related to idiopathic intracranial hypertension. Translational Vision Science and Technology, 2020. 9(3).	A
19	Eren, Y., et al., Evaluation of optic nerve head changes with optic coherence tomography in patients with idiopathic intracranial hypertension. Acta Neurologica Belgica, 2019. 119(3): p. 351-357.	A
20	Fard, M.A., et al., Optic Nerve Head and Macular Optical Coherence Tomography Measurements in Papilledema Compared With Pseudopapilledema. Journal of Neuro-Ophthalmology, 2019. 39(1): p. 28-34.	A
21	Fard, M.A., et al., Optical Coherence Tomography Angiography in Optic Disc Swelling. American journal of ophthalmology, 2018. 191: p. 116-123.	A
22	Fard, M.A., et al., Optical Coherence Tomography Angiography in Papilledema Compared With Pseudopapilledema. Investigative ophthalmology & visual science, 2019. 60(1): p. 168-175.	A
23	Fard, M.A., et al., Quantification of peripapillary total retinal volume in pseudopapilledema and mild papilledema using spectral-domain optical coherence tomography. American journal of ophthalmology, 2014. 158(1): p. 136-43.	A
24	Flores-Rodríguez, P., P. Gili, and M.D. Martín-Ríos, Sensitivity and specificity of time-domain and spectral-domain optical coherence tomography in differentiating optic nerve head drusen and optic disc oedema. Ophthalmic Physiol Opt, 2012. 32(3): p. 213-21.	A
25	Gampa, A., et al., Quantitative Association Between Peripapillary Bruch's Membrane Shape and Intracranial Pressure. Investigative ophthalmology & visual science, 2017. 58(5): p. 2739-2745.	A

For peer review only - http://bmjopen.bmj.com/site/about/guidelines.xhtml

26	García-Montesinos, J., et al., Relationship between lamina cribrosa displacement and trans-laminar pressure difference in papilledema. Graefes Arch Clin Exp Ophthalmol, 2017. 255(6): p. 1237-1243.	A
27	Geddie, B.E., U.E. Altiparmak, and E.R. Eggenberger, Cup-to-disc ratio in patients with idiopathic intracranial hypertension is smaller than that in normal subjects. Journal of neuro-ophthalmology : the official journal of the North American Neuro-Ophthalmology Society, 2010. 30(3): p. 231-4.	A
28	Gozzi, F., et al., Optical coherence tomography in the differential diagnosis of true edema versus pseudoedema of the optic disc. Acta Ophthalmologica, 2017. 95.	A
29	Group, O.C.T.S.-S.C.f.N.I.I.H.S., et al., Baseline OCT measurements in the idiopathic intracranial hypertension treatment trial, part II: correlations and relationship to clinical features. Investigative ophthalmology & visual science, 2014. 55(12): p. 8173-9.	A
30	Hamill, E., et al., Cup-to-Disc Ratio in Idiopathic Intracranial Hypertension without Papilloedema. Neuro-ophthalmology (Aeolus Press), 2014. 38(2): p. 69-73.	A
31	Hata, M. and K. Miyamoto, Causes and Prognosis of Unilateral and Bilateral Optic Disc Swelling. Neuro-Ophthalmology, 2017. 41(4): p. 187-191.	A
32	Hata, M., et al., Measurement of retinal nerve fiber layer thickness in eyes with optic disc swelling by using scanning laser polarimetry and optical coherence tomography. Clinical ophthalmology (Auckland, N.Z.), 2014. 8: p. 105-11.	A
33	Hoye, V.J., 3rd, et al., Optical coherence tomography demonstrates subretinal macular edema from papilledema. Archives of ophthalmology (Chicago, Ill. : 1960), 2001. 119(9): p. 1287-90.	A
34	Huang-Link, Y., et al., Optical coherence tomography represents a sensitive and reliable tool for routine monitoring of idiopathic intracranial hypertension with and without papilledema. European journal of neurology, 2019. 26(5): p. 808-e57.	A
35	Huang-Link, Y.M., et al., OCT measurements of optic nerve head changes in idiopathic intracranial hypertension. Clinical Neurology and Neurosurgery, 2015. 130: p. 122-127.	A
36	Igarashi, N., et al., Optic disc cupping characteristics of normal pressure hydrocephalus patients with normal-tension glaucoma. Scientific reports, 2019. 9(1): p. 3108.	A
37	Intracranial, N.I., Baseline OCT Measurements in the Idiopathic Intracranial Hypertension Treatment Trial, Part I: Quality Control, Comparisons, and Variability (vol 55, pg 8180, 2014). Investigative Ophthalmology & Visual Science, 2016. 57(15): p. 6909-6909.	A
38	Johnson, L.N., et al., Differentiating optic disc edema from optic nerve head drusen on optical coherence tomography. Archives of ophthalmology (Chicago, Ill. : 1960), 2009. 127(1): p. 45-9.	A
39	Kamil, M.A.S., et al., Association between papilledema and guillian - Barre syndrome. Indian Journal of Public Health Research and Development, 2018. 9(12): p. 549-555.	A
40	Kaufhold, F., et al., Optic nerve head quantification in idiopathic intracranial hypertension by spectral domain OCT. PloS one, 2012. 7(5): p. e36965.	A

For peer review only - http://bmjopen.bmj.com/site/about/guidelines.xhtml

41	Kim, B.H. and E.J. Lee, Optic Disc Swelling After Intraocular Pressure Lowering Treatment in Acute Primary Angle Closure. Journal of glaucoma, 2017. 26(2): p. e87-e89.	A
42	Kim, M.J. and S.P. Kelly, Optical Coherence Tomography Imaging of Paton Folds in Papilledema With Retinopathy From Systemic Hypertension. JAMA ophthalmology, 2016. 134(10): p. e162121.	A
43	Koraysha, N.A., et al., Evaluating optic nerve diameter as a possible biomarker for disability in patients with multiple sclerosis. Neuropsychiatric Disease and Treatment, 2019. 15: p. 2571-2578.	A
44	Kupersmith, M., Baseline optical coherence tomography (OCT) measurements in the idiopathic intracranial hypertension treatment trial: Correlations and relationship to clinical features. Neurology, 2014. 82(10).	A
45	Kupersmith, M., Effects of intervention on optical imaging of papilledema in the idiopathic intracranial hypertension treatment trial. Neurology, 2015. 84.	A
46	Kupersmith, M., et al., Papilledema Outcomes from the Optical Coherence Tomography Substudy of the Idiopathic Intracranial Hypertension Treatment Trial. Ophthalmology, 2015. 122(9): p. 1939-1945.	A
47	Kupersmith, M.J. and N.I.S. Grp, Baseline Optical Coherence Tomography (OCT) of Participants in the Idiopathic Intracranial Hypertension Treatment Trial: Correlations and Relationships to Clinical Features. Investigative Ophthalmology & Visual Science, 2014. 55(13): p. 2.	A
48	Kupersmith, M.J., Baseline OCT measurements in the idiopathic intracranial hypertension treatment trial, part I: Quality control, comparisons, and variability. Investigative Ophthalmology and Visual Science, 2014. 55(12): p. 8180-8188.	A
49	Kupersmith, M.J., et al., Optical coherence tomography of the swollen optic nerve head: Deformation of the peripapillary retinal pigment epithelium layer in papilledema. Investigative Ophthalmology and Visual Science, 2011. 52(9): p. 6558-6564.	A
50	Kupersmith, M.J., et al., Papilledema Outcomes from the Optical Coherence Tomography Substudy of the Idiopathic Intracranial Hypertension Treatment Trial. Ophthalmology, 2015. 122(9): p. 1939-+.	A
51	Kupersmith, M.J., et al., Scanning laser polarimetry reveals status of RNFL integrity in eyes with optic nerve head swelling by OCT. Investigative ophthalmology & visual science, 2012. 53(4): p. 1962-70.	A
52	Kupersmith, M.J., Papilledema outcome evaluation in the OCT substudy of the idiopathic intracranial hypertension treatment trial. Investigative Ophthalmology and Visual Science, 2015. 56(7): p. 2235.	A
53	Labib, D.M. and D.H.A. Raouf, Diagnostic value of optical coherence tomography in patients with idiopathic intracranial hypertension. Egyptian Journal of Neurology Psychiatry and Neurosurgery, 2015. 52(4): p. 249-253.	A
54	Laemmer, R., et al., Detection of nerve fiber atrophy in apparently effectively treated papilledema in idiopathic intracranial hypertension. Graefes Archive for Clinical and Experimental Ophthalmology, 2010. 248(12): p. 1787-1793.	A
55	Lee, K.M., S.J. Woo, and J.-M. Hwang, Differentiation of optic nerve head drusen and optic disc edema with spectral-domain optical coherence tomography. Ophthalmology, 2011. 118(5): p. 971-7.	A

For peer review only - http://bmjopen.bmj.com/site/about/guidelines.xhtml

56	Liu, K.C., et al., Venous sinus stenting for reduction of intracranial pressure in IIH: A prospective pilot study. Journal of Neurosurgery, 2017. 127(5): p. 1126-1133.	A
57	Malhotra, K., et al., Association Between Peripapillary Bruch's Membrane Shape and Intracranial Pressure: Effect of Image Acquisition Pattern and Image Analysis Method, a Preliminary Study. Frontiers in neurology, 2018. 9: p. 1137.	A
58	Margolin, E., Diagnosis and grading of papilledema in patients with raised intracranial pressure using optical coherence tomography versus clinical expert assessment using a clinical staging scale. Evidence-Based Ophthalmology, 2011. 12(1): p. 42-43.	A
59	Marzoli, S.B., et al., Quantitative analysis of optic nerve damage in idiopathic intracranial hypertension (IIH) at diagnosis. Neurological sciences : official journal of the Italian Neurological Society and of the Italian Society of Clinical Neurophysiology, 2013. 34 Suppl 1: p. S143-5.	A
60	Monteiro, M.L. and C.L. Afonso, Macular thickness measurements with frequency domain-OCT for quantification of axonal loss in chronic papilledema from pseudotumor cerebri syndrome. Eye (Lond), 2014. 28(4): p. 390-8.	A
61	Oner, A., A. Agadayi, and N. Sinim, Optik Disk Odemi ve Optik Disk Druzeni Ayriminda Spektral-Domain Optik Kohorens Tomografi KullanilmasiDifferentiating optic disc edema from optic nerve head drusen with spectral domain optical coherence tomography. Retina-Vitreus, 2015. 23(2): p. 141-144.	A
62	Optical Coherence Tomography Substudy Committee; NORDIC Idiopathic Intracranial Hypertension Study Group. Papilledema Outcomes from the Optical Coherence Tomography Substudy of the Idiopathic Intracranial Hypertension Treatment Trial. Ophthalmology, 2015. 122(9): p. 1939-45.e2.	A
63	Optical Coherence Tomography Substudy, C. and N.I.I.H.S. Group, Papilledema Outcomes from the Optical Coherence Tomography Substudy of the Idiopathic Intracranial Hypertension Treatment Trial. Ophthalmology, 2015. 122(9): p. 1939-45.e2.	A
64	Pardon LP, Cheng H, Tang RA, Saenz R, Frishman LJ, Patel NB. Custom Optical Coherence Tomography Parameters for Distinguishing Papilledema from Pseudopapilledema. Optom Vis Sci. 2019;96(8):599-608. doi:10.1097/OPX.0000000000001408	A
65	Pardon, L.P., et al., Custom Optical Coherence Tomography Parameters for Distinguishing Papilledema from Pseudopapilledema. Optometry and vision science : official publication of the American Academy of Optometry, 2019. 96(8): p. 599-608.	A
66	Pasaoglu, I., et al., Lamina cribrosa surface position in idiopathic intracranial hypertension with swept-source optical coherence tomography. Indian journal of ophthalmology, 2019. 67(7): p. 1085-1088.	A
67	Patel, M.D., et al., Methods for Quantifying Optic Disc Volume and Peripapillary Deflection Volume Using Radial Optical Coherence Tomography Scans and Association With Intracranial Pressure. Frontiers in neurology, 2019. 10: p. 798.	A
68	Pilat, A.V., et al., Macular morphology in patients with optic nerve head drusen and optic disc edema. Ophthalmology, 2014. 121(2): p. 552-7.	A

For peer review only - http://bmjopen.bmj.com/site/about/guidelines.xhtml

69	Pilat, A.V., et al., Morphology of retinal vessels in patients with optic nerve head drusen and optic disc edema. Investigative ophthalmology & visual science, 2014. 55(6): p. 3484-90.	A
70	Rebolleda, G. and F.J. Muñoz-Negrete, Follow-up of mild papilledema in idiopathic intracranial hypertension with optical coherence tomography. Invest Ophthalmol Vis Sci, 2009. 50(11): p. 5197-200.	A
71	Rodriguez Torres Y, Lee P, Mihlstin M, Tomsak RL. Correlation Between Optic Disc Peripapillary Capillary Network and Papilledema Grading in Patients With Idiopathic Intracranial Hypertension: A Study of Optical Coherence Tomography Angiography [published online ahead of print, 2020 Jan 16]. J Neuroophthalmol. 2020;10.1097/WNO.0000000000000877. doi:10.1097/WNO.0000000000000877	A
72	Saenz, R., et al., Use of A-scan Ultrasound and Optical Coherence Tomography to Differentiate Papilledema From Pseudopapilledema. Optometry and vision science : official publication of the American Academy of Optometry, 2017. 94(12): p. 1081-1089.	A
73	Sarac, O., et al., Differentiation of optic disc edema from optic nerve head drusen with spectral-domain optical coherence tomography. Journal of neuro-ophthalmology : the official journal of the North American Neuro-Ophthalmology Society, 2012. 32(3): p. 207-11.	A
74	Savini, G., et al., Detection and quantification of retinal nerve fiber layer thickness in optic disc edema using stratus OCT. Archives of Ophthalmology, 2006. 124(8): p. 1111-1117.	A
75	Scott, C.J., et al., Diagnosis and grading of papilledema in patients with raised intracranial pressure using optical coherence tomography vs clinical expert assessment using a clinical staging scale. Archives of Ophthalmology, 2010. 128(6): p. 705-711.	A
76	Sheils, C.R., et al., The Relationship Between Optic Disc Volume, Area, and Frisen Score in Patients With Idiopathic Intracranial Hypertension. American journal of ophthalmology, 2018. 195: p. 101-109.	A
77	Sibony, P., et al., Optical coherence tomography shape analysis of the peripapillary retinal pigment epithelium layer in presumed optic nerve sheath meningiomas. Journal of neuro-ophthalmology : the official journal of the North American Neuro-Ophthalmology Society, 2014. 34(2): p. 130-6.	A
78	Sibony, P., M.J. Kupersmith, and F.J. Rohlf, Shape analysis of the peripapillary RPE layer in papilledema and ischemic optic neuropathy. Investigative ophthalmology & visual science, 2011. 52(11): p. 7987-95.	A
79	Sibony, P.A., et al., Retinal and Choroidal Folds in Papilledema. Investigative ophthalmology & visual science, 2015. 56(10): p. 5670-80.	A
80	Sibony, P.A., Gaze Evoked Deformations of the Peripapillary Retina in Papilledema and Ischemic Optic Neuropathy. Investigative ophthalmology & visual science, 2016. 57(11): p. 4979-4987.	A
81	Sinclair, A.J., et al., Low energy diet and intracranial pressure in women with idiopathic intracranial hypertension: prospective cohort study. BMJ (Clinical research ed.), 2010. 341: p. c2701.	A
82	Skau, M., et al., Disease activity in idiopathic intracranial hypertension: A 3-month follow-up study. Journal of Neurology, 2011. 258(2): p. 277-283.	A

For peer review only - http://bmjopen.bmj.com/site/about/guidelines.xhtml

83	Skau, M., et al., OCT for optic disc evaluation in idiopathic intracranial hypertension. Graefe's Archive for Clinical and Experimental Ophthalmology, 2011. 249(5): p. 723-730.	A
84	Smith, K.A., et al., A case series of dural venous sinus stenting in idiopathic intracranial hypertension: association of outcomes with optical coherence tomography¹. International Journal of Neuroscience, 2017. 127(2): p. 145-153.	A
85	Smith, S.V. and D.I. Friedman, The Idiopathic Intracranial Hypertension Treatment Trial: a Review of the Outcomes. Headache, 2017. (no pagination).	A
86	Starks, V., et al., Effect of optic nerve sheath fenestration for idiopathic intracranial hypertension on retinal nerve fiber layer thickness. Orbit (Amsterdam, Netherlands), 2016. 35(2): p. 87-90.	A
87	Steinegger, K., C. Bergin, and Y. Guex-Crosier, Malignant hypertension: clinical manifestations of 7 cases. Klinische Monatsblatter fur Augenheilkunde, 2015. 232(4): p. 590-2.	A
88	Tatar, I.T., et al., Morphological assessment of lamina cribrosa in idiopathic intracranial hypertension. Indian journal of ophthalmology, 2020. 68(1): p. 164-167.	A
89	Teismann, N., et al., Point-of-care ocular ultrasound to detect optic disc swelling. Academic Emergency Medicine, 2013. 20(9): p. 920-925.	A
90	Tirakotai, W., et al., Endoscopic Optic Nerve Sheath Fenestration for Treatment of Papilledema Secondary to Intracranial Venous Hypertension: Report of Two Cases. Journal of the Medical Association of Thailand = Chotmaihet thangphaet, 2016. 99 Suppl 3: p. S141-6.	A
91	Tsikata, E., et al., Volumetric Measurement of Optic Nerve Head Drusen Using Swept-Source Optical Coherence Tomography. Journal of Glaucoma, 2017. 26(9): p. 798-804.	A
92	Verma, R., et al., Ophthalmological manifestation in patients of tuberculous meningitis. QJM : monthly journal of the Association of Physicians, 2019. 112(6): p. 409-419.	A
93	Waisbourd, M., et al., OCT assessment of morphological changes of the optic nerve head and macula in idiopathic intracranial hypertension. Clinical neurology and neurosurgery, 2011. 113(10): p. 839-43.	A
94	Wall, M., et al., Threshold Static Automated Perimetry of the Full Visual Field in Idiopathic Intracranial Hypertension. Investigative Ophthalmology & Visual Science, 2019. 60(6): p. 1898-1905.	A
95	Wang, J.K., et al., Peripapillary retinal pigment epithelium layer shape changes from acetazolamide treatment in the idiopathic intracranial hypertension treatment trial. Investigative Ophthalmology and Visual Science, 2017. 58(5): p. 2554-2565.	A
96	Wang, J.K., et al., Three-dimensional bruch's membrane shape change over time with acetazolamide treatment in the idiopathic intracranial hypertension treatment trial (IIHTT). Investigative Ophthalmology and Visual Science, 2017. 58(8).	A
97	Wartak, A., et al., Investigating spontaneous retinal venous pulsation using Doppler optical coherence tomography. Scientific Reports, 2019. 9: p. 11.	A
98	Yri, H.M., et al., The course of headache in idiopathic intracranial hypertension: a 12-month prospective follow-up study. Eur J Neurol, 2014. 21(12): p. 1458-64.	A

For peer review only - http://bmjopen.bmj.com/site/about/guidelines.xhtml

99	Bahnasy, W.S., et al., Neuro-ophthalmological biomarkers of visual outcome in newly diagnosed idiopathic intracranial hypertension. Egyptian Journal of Neurology Psychiatry and Neurosurgery, 2019. 55(1): p. 8.	B
100	Casas, P., et al., Retinal nerve fibre layer evaluation using optical coherence tomography in patients with obstructive sleep apnea syndrome (OSAS). Neuro-Ophthalmology, 2011. 35.	B
101	Chang, Y.C., et al., Relationship Between Optic Nerve Protrusion Measured by OCT and MRI and Papilledema Severity. Invest Ophthalmol Vis Sci, 2015. 56(4): p. 2297-302.	B
102	Chen, J.J., et al., Causes and Prognosis of Visual Acuity Loss at the Time of Initial Presentation in Idiopathic Intracranial Hypertension. Investigative Ophthalmology & Visual Science, 2015. 56(6): p. 3850-3859.	B
103	Chen, Q., et al., Pseudotumour Cerebri Syndrome in China: A Cohort Study. Scientific reports, 2020. 10(1): p. 1222.	B
104	Figus, M., et al., Optical Coherence Tomography in Patients with Chiari I Malformation. Biomed Research International, 2015: p. 7.	B
105	Ghasemi Falavarjani K, Tian JJ, Akil H, Garcia GA, Sadda SR, Sadun AA. SWEPT-SOURCE OPTICAL COHERENCE TOMOGRAPHY ANGIOGRAPHY OF THE OPTIC DISK IN OPTIC NEUROPATHY. Retina. 2016;36 Suppl 1:S168-S177. doi:10.1097/IAE.0000000000001259	B
106	Goldhagen BE, Bhatti MT, Srinivasan PP, Chiu SJ, Farsiu S, El-Dairi MA. Retinal atrophy in eyes with resolved papilledema detected by optical coherence tomography. J Neuroophthalmol. 2015;35(2):122-126. doi:10.1097/WNO.0000000000000210	B
107	Goldhagen, B.E., et al., Retinal atrophy in eyes with resolved papilledema detected by optical coherence tomography. Journal of Neuro-Ophthalmology, 2015. 35(2): p. 122-123.	B
108	Ju, D.G., et al., Clinical Significance of Tumor-Related Edema of Optic Tract Affecting Visual Function in Patients with Sellar and Suprasellar Tumors. World Neurosurg, 2019. 132: p. e862-e868.	B
109	Karam, E.Z. and T.R. Hedges, Optical coherence tomography of the retinal nerve fibre layer in mild papilloedema and pseudopapilloedema. The British journal of ophthalmology, 2005. 89(3): p. 294-8.	B
110	Kasl, Z., et al., [The Current Diagnostic Possibilities and Cooperation of Oftalmologist and Neurologist Concerning in Patients with Idiopatic Intracranial Hypertension]. Soucasne moznosti oftalmologicke diagnostiky a spoluprace oftalmologa s neurologem u pacientu s idiopatickou intrakranialni hypertenzi., 2016. 72(2): p. 32-8.	B
111	Kim, M.S., et al., Morphologic Features of Buried Optic Disc Drusen on En Face Optical Coherence Tomography and Optical Coherence Tomography Angiography. American Journal of Ophthalmology, 2020. 213: p. 125-133.	B
112	Koktekir, E., et al., Resolution of papilledema after endoscopic third ventriculostomy versus cerebrospinal fluid shunting in hydrocephalus: A comparative study: Clinical article. Journal of Neurosurgery, 2014. 120(6): p. 1465-1470.	B
113	Kulkarni, K.M., et al., Differentiating mild papilledema and buried optic nerve head drusen using spectral domain optical coherence tomography. Ophthalmology, 2014. 121(4): p. 959-63.	B

For peer review only - http://bmjopen.bmj.com/site/about/guidelines.xhtml

114	Martinez, M.R. and A. Ophir, Optical coherence tomography as an adjunctive tool for diagnosing papilledema in young patients. Journal of pediatric ophthalmology and strabismus, 2011. 48(3): p. 174-81.	B
115	Merticariu, C.I., et al., Optical coherence tomography assessment of structural changes in the optic nerve head and peripapillary retina in idiopathic intracranial hypertension. Archives of the Balkan Medical Union, 2019. 54(2): p. 267-273.	B
116	Rizzo, J.L., et al., Perimetry, retinal nerve fiber layer thickness and papilledema grade after cerebrospinal fluid shunting in patients with idiopathic intracranial hypertension. Journal of neuro-ophthalmology : the official journal of the North American Neuro-Ophthalmology Society, 2015. 35(1): p. 22-5.	B
117	Sajjadi, F., et al., New predictive value of optical coherence tomography analysis in the diagnosis of idiopathic intracranial hypertension. Journal of Contemporary Medical Sciences, 2017. 3(10): p. 197-207.	B
118	Santos, M.A.K., et al., [Optic disc changes by optical coherence tomography in optic disc edema in Lomé]. J Fr Ophtalmol, 2017. 40(4): p. 314-318.	B
119	Sibony, P., et al., Effects of lowering cerebrospinal fluid pressure on the shape of the peripapillary retina in intracranial hypertension. Investigative Ophthalmology and Visual Science, 2014. 55(12): p. 8223-8231.	B
120	Tang, L., et al., Quantitative evaluation of papilledema from stereoscopic color fundus photographs. Investigative Ophthalmology and Visual Science, 2012. 53(8): p. 4490-4497.	B
121	Tang, Y., et al., Assessment of visual function by optical coherence tomography and visual field for craniopharyngioma patients. International Eye Science, 2015. 15(9): p. 1621-1624.	B
122	Tüntaş Bilen, F. and H. Atilla, Peripapillary Vessel Density Measured by Optical Coherence Tomography Angiography in Idiopathic Intracranial Hypertension. J Neuroophthalmol, 2019. 39(3): p. 319-323.	B
123	Vartin C, V., et al., Detection of mild papilloedema using spectral domain optical coherence tomography. The British journal of ophthalmology, 2012. 96(3): p. 375-9.	B
124	Wang, J.K., et al., Automated quantification of volumetric optic disc swelling in papilledema using spectral-domain optical coherence tomography. Investigative Ophthalmology and Visual Science, 2012. 53(7): p. 4069-4075.	B
125	Zhang, L., C. Sun, and X. Sun, [The clinical features and value of macular ganglion cell complex thickness patterns in patients with optic chiasma lesion]. Zhonghua Yan Ke Za Zhi, 2016. 52(5): p. 335-42.	B
126	Afonso, C., et al., Evaluation of inner retinal thicknesses in the macula of patients with chronic papilledema from pseudotumor cerebri syndrome using frequency domain OCT. Investigative Ophthalmology and Visual Science, 2013. 54(15).	C
127	Agne, J., et al., Automatic Detection of Folds and Wrinkles Due to Swelling of the Optic Disc, in Fetal, Infant and Ophthalmic Medical Image Analysis, M.J. Cardoso and T. Arbel, Editors. 2017, Springer International Publishing Ag: Cham. p. 235-242.	C
128	Agne, J., et al., Determining Degree of Optic Nerve Edema from Color Fundus Photography, in Medical Imaging 2015: Computer-Aided	C

For peer review only - http://bmjopen.bmj.com/site/about/guidelines.xhtml

	Diagnosis, L.M. Hadjiiski and G.D. Tourassi, Editors. 2015, Spie-Int Soc Optical Engineering: Bellingham.	
129	Agne, J., et al., Monitoring Intracranial Pressure By a Semi-automated Determination of Bruch's Membrane Deformation From Routine SD-OCT Volume Scans of the Optic Disc. Investigative Ophthalmology & Visual Science, 2016. 57(12): p. 3.	C
130	Chang, L., et al., Optical coherence tomography in the evaluation of neurofibromatosis type 1 subjects with optic pathway gliomas. Journal of AAPOS, 2010. 14(1).	C
131	Chang, L., et al., Optical coherence tomography in the evaluation of neurofibromatosis type 1 subjects with optic pathway gliomas. Journal of AAPOS, 2010. 14(1).	C
132	Chang, M., et al., Accuracy of ultrasonography, fundus photography, autofluorescence (AF), fluorescein angiography (FA), and optical coherence tomography (OCT) in differentiating pseudopapilledema from true optic disk edema (ODE) in children. Journal of AAPOS, 2017. 21(4).	C
133	Christodoulou, L., et al., Review of the pathway for children with '? Swollen' optic discs; A service evaluation and quality improvement study. Developmental Medicine and Child Neurology, 2019. 61: p. 92.	C
134	Cohen, Y., et al., OCT imaging of papilledema in pediatric idiopathic intracranial hypertension. Investigative Ophthalmology and Visual Science, 2016. 57(12): p. 4546.	C
135	Dahlmann-Noor, A.H., et al., Ultrasonography, OCT and OCT-angiography in the diagnostic workup of children with suspected papilledema. Investigative Ophthalmology and Visual Science, 2017. 58(8).	C
136	D'Antona, L., et al., Spontaneous retinal venous pulsation: Towards non-invasive assessment of ICP. Fluids and Barriers of the CNS, 2018. 15.	C
137	D'Antona, L., et al., Spontaneous retinal venous pulsation: Towards noninvasive assessment of intracranial pressure. British Journal of Neurosurgery, 2018. 32(3): p. 336.	C
138	Duman, R., et al., Evaluation of ganglion cell layer and nerve fibre layer changes in patients with idiopathic intracranial hypertension. Neuro-Ophthalmology, 2015. 39.	C
139	Falavarjani, K.G., et al., Swept-source optical coherence tomography angiography of the optic disk in optic neuropathy. Retina, 2016. 36.	C
140	Fell, D., et al., Perfused large vessel and capillary densities in various grades of papilledema using OCTA custom software. Investigative Ophthalmology and Visual Science, 2017. 58(8).	C
141	Gelener, P., et al., Optic coherence tomography for optic disc and clinical evaluation in idiopathic intracranial hypertension. Journal of the Neurological Sciences, 2013. 333.	C
142	Gobuty, M., et al., Optical coherence tomography (OCT) use in assessing hydrocephalus. Investigative Ophthalmology and Visual Science, 2014. 55(13): p. 3362.	C
143	Gronlund, M.A., L. Mybeck, and S. Andersson, Imaging of the retina and optic nerve using optical coherence tomography in adolescents with surgically treated hydrocephalus. European Journal of Ophthalmology, 2017. 27(5).	C
144	Gupta, A., et al., First Peak fractal analysis of optical coherence tomography angiography in eyes with papilledema. Investigative Ophthalmology and Visual Science, 2018. 59(9).	C

For peer review only - http://bmjopen.bmj.com/site/about/guidelines.xhtml

145	Hacker, M., et al., Segmentation of the surfaces of the retinal layer from OCT images. Medical image computing and computer-assisted intervention : MICCAI ... International Conference on Medical Image Computing and Computer-Assisted Intervention, 2006. 9: p. 800-807.	C
146	Hajjar, D., P. Pujol, and G. Julio, Optical coherence tomography (OCT) utility in diagnosis and follow-up of papilloedema. Neuro-Ophthalmology, 2011. 35.	C
147	Homma, T.K., et al., Optical coherence tomography (OCT) as a tool to identify idiopathic intracranial hypertension in pediatric patients under GH therapy. Endocrine Reviews, 2014. 35.	C
148	Ioyleva, E. and E. Kabanova, OCT- Angiography: Perspectives of differential diagnostics of pathology optic nerve. Acta Ophthalmologica, 2018. 96: p. 55.	C
149	Ioyleva, E., E. Kabanova, and M. Krivosheeva, Measurement of macular ganglion cell-innerplexiform layer with spectral-domain optical coherence tomography in patients with optic nerve head drusen and papilledema. Acta Ophthalmologica, 2018. 96: p. 55.	C
150	Islam, M.S., et al., Deep learning based retinal blood vessel segmentation of multiple optical coherence tomography en-face images in cases of optic disc swelling. Investigative Ophthalmology and Visual Science, 2019. 60(9).	C
151	Iwase, T., et al., Serum level of vascular endothelial growth factor and opening lumbar pressure significantly correlated with optic disc edema in patients with POEMS syndrome. Investigative Ophthalmology and Visual Science, 2015. 56(7): p. 3880.	C
152	Jenkins, K.S., C.J. Layton, and M.K.M. Adams, Ophthalmoscopic and video OCT methods to detect spontaneous venous pulsation in individuals with apparently normal intracranial pressure: The rebirth of the SVP? Acta Ophthalmologica, 2017. 95.	C
153	Jensen, R. and M. Skau, Disease activity in idiopathic intracranial hypertension: A 3-month follow-up study. Journal of Headache and Pain, 2010. 11.	C
154	Jiramongkolchai, K., et al., Longitudinal Follow-Up of Papilledema by Optical Coherence Tomography Macular Segmentation. Investigative Ophthalmology & Visual Science, 2016. 57(12): p. 2.	C
155	Kapila, A., V. Lal, and A. Gupta, Clinical and neuro-ophthalmologic predictors of visual outcome in idiopathic intracranial hypertension (IIH). Journal of the Neurological Sciences, 2015. 357.	C
156	Katz, S.E., A.M. Mahmoud, and C.J. Roberts, Objective quantification of papilledema resolution after optic nerve sheath fenestration. Investigative Ophthalmology and Visual Science, 2017. 58(8).	C
157	Kedar, S., et al., Effects of acute intracranial pressure change on human and pig optic nerve head using optical coherence tomography. Investigative Ophthalmology & Visual Science, 2017. 58(8): p. 3.	C
158	Kee, H.J., S.J. Woo, and J.M. Hwang, Longitudinal change of buried optic disc drusen in children. Journal of AAPOS, 2019. 23(4).	C
159	Krauss, H.R., et al., The clinical utility of ocular coherence tomography in evaluation and management of skull base disorders. Journal of Neurological Surgery Part B: Skull Base, 2017. 78.	C

For peer review only - http://bmjopen.bmj.com/site/about/guidelines.xhtml

160	Krivosheeva, M., E. Ioyleva, and E. Kabanova, Measurement of macula thickness and macular ganglion cell with SS-OCT in patients with mild papille. Ophthalmic Research, 2018. 60: p. 14.	C
161	Kupersmith, M., R. Kardon, and M. Durbin, Retinal nerve fiber layer alteration differences between OCT and scanning laser polarimetry with optic nerve head swelling. Neurology, 2012. 78(1).	C
162	Lascaratos, G. and S. Madill, Patients With Idiopathic Intracranial Hypertension Have Significantly Larger Optic Nerve Heads Than Controls. Investigative Ophthalmology & Visual Science, 2010. 51(13): p. 2.	C
163	Lenaghan, P., et al., Point-of-care ocular sonography to detect optic disc swelling. Annals of Emergency Medicine, 2011. 58(4).	C
164	Malem, A., S. West, and G. De Salvo, Multicolor imaging in the assessment and diagnosis of optic disc swelling. Investigative Ophthalmology and Visual Science, 2015. 56(7): p. 2238.	C
165	Martinez-Alvarez, L., et al., Peripapillary choroidal neovascularization in papilloedema: Report of two cases and review of the literature. Neuro-Ophthalmology, 2015. 39.	C
166	McHugh, J., et al., Use of the heidelberg spectralis oct device to assess spontaneous venous pulsation in patients with headaches. Neuro-Ophthalmology, 2017. 41.	C
167	McNabb, R.P., et al., Widefield whole eye ssoct measurement of retinal curvature for screening of elevated intracranial pressure. Investigative Ophthalmology and Visual Science, 2018. 59(9).	C
168	Miller, E.R., C.O. Jordan, and D.L. Rogers, Optic nerve optical coherence tomography versus humphrey visual field analysis in pediatric intracranial hypertension. Journal of AAPOS, 2014. 18(4).	C
169	Miller, J.W., et al., Differentiation between papilledema and nonarteritic anterior ischemic optic neuropathy using retinal layer shape and regional volume features in spectral-domain optical coherence tomography. Investigative Ophthalmology and Visual Science, 2018. 59(9).	C
170	Monroy, J.E.G., et al., Optic nerve volumetric measurements in pediatric papilledema with spectral domain OCT. Journal of AAPOS, 2014. 18(4).	C
171	Morrow, M., F. Villanueva, and F. Abukhalil, Evolution of macular findings on optical coherence tomography (OCT) in papilledema. Neurology, 2014. 82(10).	C
172	Morrow, M., The relationship between optical coherence tomographic (OCT) and perimetric findings in patients with papilledema. Neurology, 2014. 82(10).	C
173	Morrow, M., The relationship between optical coherence tomographic and perimetric findings in patients with papilledema. Journal of the Neurological Sciences, 2013. 333.	C
174	Moss, H., et al., Optic nerve head geometry as a function of chronic intracranial pressure. Investigative Ophthalmology and Visual Science, 2017. 58(8).	C
175	Moss, H., J.J. McAnany, and J.C. Park, Structure function relationships in idiopathic intracranial hypertension. Investigative Ophthalmology and Visual Science, 2015. 56(7): p. 2230.	C
176	Nayfach, J., et al., Integrating funduscopy, optical coherence tomography (OCT), and ultrasound to differentiate optic disc edema (ODE) from pseudo optic disc edema (PODE). Investigative Ophthalmology and Visual Science, 2016. 57(12): p. 4541.	C

For peer review only - http://bmjopen.bmj.com/site/about/guidelines.xhtml

177	Pak, K., et al., Anatomical and functional changes of optic disc and retinal nerve fiber layer after bariatric surgery in morbidly obese Korean patients with lower BMI. Investigative Ophthalmology and Visual Science, 2019. 60(9).	C
178	Parhiz, A.T., P. Sibony, and M. Kupersmith, SD-OCT in monitoring intracranial pressure in patients with CSF shunts. Investigative Ophthalmology and Visual Science, 2013. 54(15).	C
179	Pascoe, E., et al., A case series of intracranial hypertension in patients with Turner syndrome, with and without growth hormone therapy. International Journal of Pediatric Endocrinology, 2015. 2015.	C
180	Pathmanathan, N., et al., Audit of intracranial pressure monitoring in traumatic brain injury. Intensive care medicine, 2013. 39: p. S259-.	C
181	Pemp, B., et al., Changes of choroidal thickness as measured with spectral domain OCT in patients with idiopathic intracranial hypertension after lowering of intracranial pressure. Investigative Ophthalmology and Visual Science, 2013. 54(15).	C
182	Pemp, B., et al., Measurements by spectral domain oct in papilledema are associated with reduced retinal nerve fiber layer thickness after resolution of optic Disc swelling. Investigative Ophthalmology and Visual Science, 2014. 55(13): p. 5779.	C
183	Pemp, B., et al., The course of divergence paresis and papilloedema in patients with increased intracranial pressure. Neuro-Ophthalmology, 2015. 39.	C
184	Permeswaran, Y.M., et al., An en-face OCT registration approach for locating the center of bruch's membrane opening in longitudinal papilledema cases. Investigative Ophthalmology and Visual Science, 2019. 60(9).	C
185	Pilat, A., et al., Macula structure in patients with optic disc oedema and optic nerve head drusen. Investigative Ophthalmology and Visual Science, 2013. 54(15).	C
186	Reitner, A., B. Pemp, and K. Kircher, Retinal nerve fibre layer thickness measurement using spectral domain OCT in various optic nerve lesions. Neuro-Ophthalmology, 2011. 35.	C
187	Sarrafpour, S., et al., Fractal analysis of peripapillary vasculature in eyes with papilledema using optical coherence tomography angiography. Investigative Ophthalmology and Visual Science, 2017. 58(8).	C
188	Scott, L.L., et al., Correlation of opening pressure and Frisen grade of papilledema in pediatric patients with intracranial hypertension. Investigative Ophthalmology and Visual Science, 2015. 56(7): p. 2231.	C
189	Skau, M., et al., Diagnostic value of optical coherence tomography for intracranial pressure in idiopathic intracranial hypertension. Journal of Headache and Pain, 2013. 14(1).	C
190	Stark, R.M., et al., Assessment of intracranial pressure during venous sinus stenting. Journal of Neurological Surgery, Part B: Skull Base, 2016. 77.	C
191	Starke, R.M., et al., A prospective pilot study of intraparenchymal & intravenous cerebral pressure assessment during venous sinus stenting. Journal of Neurosurgery, 2016. 124(4).	C
192	Stroet, A., et al., Optical coherence tomography in idiopathic intracranial hypertension-first experiences. Neuropediatrics, 2012. 43(2).	C
193	Su, Q.Y., et al., Spectral-Domain Optical Coherence Tomography Optic-Nerve-Head and Macular En-Face Image Registration in Cases of	C

For peer review only - http://bmjopen.bmj.com/site/about/guidelines.xhtml

	Papilledema. Investigative Ophthalmology & Visual Science, 2017. 58(8): p. 3.	
194	Swinkin, E., A. Sundaram, and J.M. Pejman, Unilateral papilledema in idiopathic intracranial hypertension: A report of two cases and literature review. Neurology, 2017. 88(16).	C
195	Tang, R., et al., Optical coherence tomography visualization of optic nerve head structure detects acute changes in the intracranial pressure. Neurology, 2014. 82(10).	C
196	Thompson, D.A., et al., Outsized paediatric VEPs. Documenta Ophthalmologica, 2017. 135(1): p. 24.	C
197	Van De Beeten, S.D.C., et al., Increased total retinal thickness on OCT: A precursor for intracranial hypertension? Child's Nervous System, 2018. 34(5): p. 1006-1007.	C
198	Vangipuram, G. and H. Moss, Changes in basement membrane opening displacement within 1 hour following intracranial pressure lowering in subjects with and without idiopathic intracranial hypertension. Investigative Ophthalmology and Visual Science, 2015. 56(7): p. 2232.	C
199	Varma, H., et al., Longitudinal analysis of optic nerve edema, retinal nerve fiber layer thickness, and visual field mean deviation in pediatric patients with intracranial hypertension. Journal of AAPOS, 2019. 23(4).	C
200	Wall, M., et al., Detection of visual loss in IIH with static automated perimetry: Temporal wedge defects. Investigative Ophthalmology and Visual Science, 2019. 60(9).	C
201	Wan, M.J., Correlation between spectral domain optical coherence tomography (SD-OCT) and long-term vision loss in children with papilledema. Journal of AAPOS, 2019. 23(4).	C
202	Wang, J.K., et al., Automated 3-D Region-Based Volumetric Estimation of Optic Disc Swelling in Papilledema Using Spectral-Domain Optical Coherence Tomography, in Medical Imaging 2013: Biomedical Applications in Molecular, Structural, and Functional Imaging, J.B. Weaver and R.C. Molthen, Editors. 2013, Spie-Int Soc Optical Engineering: Bellingham.	C
203	Wang, J.K., et al., Automated Classification of Retinal Folds and Wrinkles in En-Face Optical Coherence Tomography Images with Optic Disc Swelling. Investigative ophthalmology & visual science, 2019. 60(9).	C
204	Wang, J.K., et al., Automated detection of retinal folds in papilledema using en-face spectral-domain optical coherence tomography. Investigative Ophthalmology and Visual Science, 2018. 59(9).	C
205	Wang, J.K., et al., Change in the deflection of the neural canal opening over time with acetazolamide treatment in idiopathic intracranial hypertension. Investigative Ophthalmology and Visual Science, 2015. 56(7): p. 2233.	C
206	Wang, J.K., et al., Regional spectral-domain optical coherence tomography features better predict Frisen scale grades than total volume alone in papilledema. Investigative Ophthalmology and Visual Science, 2013. 54(15).	C
207	Wang, J.K., et al., Semi-Automated 2D Bruch's Membrane Shape Analysis in Papilledema Using Spectral-Domain Optical Coherence Tomography, in Medical Imaging 2015: Biomedical Applications in Molecular, Structural, and Functional Imaging, B. Gimi and R.C. Molthen, Editors. 2015, Spie-Int Soc Optical Engineering: Bellingham.	C

For peer review only - http://bmjopen.bmj.com/site/about/guidelines.xhtml

208	Wang, J.K., R.H. Kardon, and M.K. Garvin, Combined Use of High-Definition and Volumetric Optical Coherence Tomography for the Segmentation of Neural Canal Opening in Cases of Optic Nerve Edema, in Medical Imaging 2015: Image Processing, S. Ourselin and M.A. Styner, Editors. 2015, Spie-Int Soc Optical Engineering: Bellingham.	C
209	Weaver, M.A., et al., Deformation of the peripapillary retinal pigmented epithelium layer on OCT: a useful tool to identify intracranial hypertension in a pediatric population? Journal of AAPOS, 2019. 23(4).	C
210	Yri, H., et al., EHMTI-0155. The course of headache in idiopathic intracranial hypertension: A 12 month prospective follow-up study. Journal of Headache and Pain, 2014. 15.	C
211	Yri, H., et al., Idiopathic intracranial hypertension is not benign; a prospective long-term follow-up study. Journal of Headache and Pain, 2013. 14(1).	C
212	Kleinberg, T.T. and J.R. Bilyk, Evolution of Disc Edema in Thyroid-Related Compressive Optic Neuropathy. Ophthalmic plastic and reconstructive surgery, 2016. 32(4): p. e100.	D
213	Perez-Lopez, M., D.S. Ting, and L. Clarke, Lamina cribrosa displacement after optic nerve sheath fenestration in idiopathic intracranial hypertension: a new tool for monitoring changes in intracranial pressure? Br J Ophthalmol, 2014. 98(11): p. 1603-4.	D
214	Rezkallah, A., et al., Hypertensive choroidopathy: Multimodal imaging and the contribution of wide-field swept-source oct-angiography. American journal of ophthalmology case reports, 2019. 13: p. 131-135.	D
215	Santos-Bueso, E., et al., [Idiopathic intracranial hypertension. Analysis by macular segmentation]. An Pediatr (Barc), 2015. 82(5): p. e230-3.	D
216	Shuaib, M.M., et al., Optical coherence tomography of the optic nerve head before and after optic nerve sheath fenestration for idiopathic intracranial hypertension. Acta neurologica Belgica, 2020.	D
217	Sibony, P.A. and M.J. Kupersmith, Paton's Folds revisited: Wrinkles, folds and creases in papilledema. Investigative Ophthalmology and Visual Science, 2016. 57(12): p. 4553.	D
218	Fard, M.A., Bruch's membrane opening on optical coherence tomography in pediatric papilledema and pseudopapilledema. Journal of AAPOS : the official publication of the American Association for Pediatric Ophthalmology and Strabismus, 2018. 22(3): p. 247.	E
219	García-Montesinos, J., et al., Bruch's membrane opening on optical coherence tomography in pediatric papilledema and pseudopapilledema. J aapos, 2018. 22(3): p. 247-248.	E
220	Ophir, A., Optical coherence tomography as an adjunctive tool for diagnosing pediatric pseudotumor cerebri. Journal of AAPOS, 2008. 12(4): p. 421-422.	E
221	Savini, G., et al., Optical coherence tomography for optic disc edema. Archives of Ophthalmology, 2011. 129(9): p. 1245-1246.	E

For peer review only - http://bmjopen.bmj.com/site/about/guidelines.xhtml

PRISMA 2009 Checklist

Section/topic	#	Checklist item	Reported on page #
**TITLE**			
Title	1	Identify the report as a systematic review, meta-analysis, or both.	1
**ABSTRACT**			
Structured summary	2	Provide a structured summary including, as applicable: background; objectives; data sources; study eligibility criteria, participants, and interventions; study appraisal and synthesis methods; results; limitations; conclusions and implications of key findings; systematic review registration number.	2
**INTRODUCTION**			
Rationale	3	Describe the rationale for the review in the context of what is already known.	3
Objectives	4	Provide an explicit statement of questions being addressed with reference to participants, interventions, comparisons, outcomes, and study design (PICOS).	3-4
**METHODS**			
Protocol and registration	5	Indicate if a review protocol exists, if and where it can be accessed (e.g., Web address), and, if available, provide registration information including registration number.	4
Eligibility criteria	6	Specify study characteristics (e.g., PICOS, length of follow-up) and report characteristics (e.g., years considered, language, publication status) used as criteria for eligibility, giving rationale.	4
Information sources	7	Describe all information sources (e.g., databases with dates of coverage, contact with study authors to identify additional studies) in the search and date last searched.	4
Search	8	Present full electronic search strategy for at least one database, including any limits used, such that it could be repeated.	4
Study selection	9	State the process for selecting studies (i.e., screening, eligibility, included in systematic review, and, if applicable, included in the meta-analysis).	4
Data collection process	10	Describe method of data extraction from reports (e.g., piloted forms, independently, in duplicate) and any processes for obtaining and confirming data from investigators.	4
Data items	11	List and define all variables for which data were sought (e.g., PICOS, funding sources) and any assumptions and simplifications made.	4
Risk of bias in individual studies	12	Describe methods used for assessing risk of bias of individual studies (including specification of whether this was done at the study or outcome level), and how this information is to be used in any data synthesis.	4
Summary measures	13	State the principal summary measures (e.g., risk ratio, difference in means).	4
Synthesis of results	14	Describe the methods of handling data and combining results of studies, if done, including measures of consistency (e.g., I^2) for each meta-analysis.	N/A

For peer review only - http://bmjopen.bmj.com/site/about/guidelines.xhtml

PRISMA 2009 Checklist

Section/topic	#	Checklist item	Reported on page #
Risk of bias across studies	15	Specify any assessment of risk of bias that may affect the cumulative evidence (e.g., publication bias, selective reporting within studies).	4
Additional analyses	16	Describe methods of additional analyses (e.g., sensitivity or subgroup analyses, meta-regression), if done, indicating which were pre-specified.	N/A
**RESULTS**			
Study selection	17	Give numbers of studies screened, assessed for eligibility, and included in the review, with reasons for exclusions at each stage, ideally with a flow diagram.	5
Study characteristics	18	For each study, present characteristics for which data were extracted (e.g., study size, PICOS, follow-up period) and provide the citations.	5-7
Risk of bias within studies	19	Present data on risk of bias of each study and, if available, any outcome level assessment (see item 12).	5-7
Results of individual studies	20	For all outcomes considered (benefits or harms), present, for each study: (a) simple summary data for each intervention group (b) effect estimates and confidence intervals, ideally with a forest plot.	5-7
Synthesis of results	21	Present results of each meta-analysis done, including confidence intervals and measures of consistency.	N/A
Risk of bias across studies	22	Present results of any assessment of risk of bias across studies (see Item 15).	5
Additional analysis	23	Give results of additional analyses, if done (e.g., sensitivity or subgroup analyses, meta-regression [see Item 16]).	N/A
**DISCUSSION**			
Summary of evidence	24	Summarize the main findings including the strength of evidence for each main outcome; consider their relevance to key groups (e.g., healthcare providers, users, and policy makers).	9-10
Limitations	25	Discuss limitations at study and outcome level (e.g., risk of bias), and at review-level (e.g., incomplete retrieval of identified research, reporting bias).	9-10
Conclusions	26	Provide a general interpretation of the results in the context of other evidence, and implications for future research.	11
**FUNDING**			
Funding	27	Describe sources of funding for the systematic review and other support (e.g., supply of data); role of funders for the systematic review.	12

From: Moher D, Liberati A, Tetzlaff J, Altman DG, The PRISMA Group (2009). Preferred Reporting Items for Systematic Reviews and Meta-Analyses: The PRISMA Statement. PLoS Med 6(7): e1000097. doi:10.1371/journal.pmed1000097

For more information, visit: **www.prisma-statement.org**.

For peer review only - http://bmjopen.bmj.com/site/about/guidelines.xhtml
